# Mono- and multimeric PSMA-targeting small molecule-thorium-227 conjugates for optimized efficacy and biodistribution in preclinical models

**DOI:** 10.1007/s00259-023-06474-z

**Published:** 2023-10-26

**Authors:** Niels Böhnke, Bård Indrevoll, Stefanie Hammer, Alex Papple, Alexander Kristian, Hans Briem, Arif Celik, Dominik Mumberg, Alan Cuthbertson, Sabine Zitzmann-Kolbe

**Affiliations:** 1 Pharmaceuticals, Bayer AG, 13342 Berlin, Germany; 2 Bayer AS, Oslo, Norway; 3 Present Address: Adcendo ApS, Copenhagen, Denmark

**Keywords:** Prostate specific membrane antigen, Targeted alpha therapy, Small molecule conjugates, Thorium-227, Prostate cancer

## Abstract

**Purpose:**

PSMA (prostate-specific membrane antigen) is highly expressed on prostate cancer (PrCa) cells and extensively used as a homing target for PrCa treatment. Most prominently, PSMA-targeting conjugate PSMA-617, carrying a DOTA chelator and labeled with therapeutic radionuclides like beta-emitting lutetium-177 or alpha-emitting actinium-225, has shown clinical activity in PrCa patients. We sought to develop PSMA-targeting small molecule (SMOL) conjugates that show high uptake in PSMA-expressing tumors and fast clearance, and can easily be labeled with the alpha emitter thorium-227 (half-life 18.7 days).

**Methods:**

A novel linker motif with improved competition against ^3^H-PSMA-617 on PSMA-expressing LNCaP cells was identified. A 2,3-hydroxypyridinone chelator modified with carboxyl groups (carboxy-HOPO) with increased hydrophilicity and robust labeling with thorium-227 was developed and allowed the synthesis of mono-, di-, tri-, and tetrameric conjugates. The resulting monomeric and multimeric PSMA SMOL-TTCs (targeted thorium conjugate) were evaluated for cellular binding, internalization, and antiproliferative activity. The in vivo antitumor efficacy of the PSMA SMOL-TTCs was determined in ST1273 and KUCaP-1 PrCa models in mice, and their biodistribution was assessed in cynomolgus monkeys, minipigs, and mice.

**Results:**

The monomeric and multimeric PSMA SMOL conjugates were readily labeled with thorium-227 at room temperature and possessed high stability and good binding, internalization, and antiproliferative activity in vitro. In vivo, the monomeric, dimeric, and trimeric PSMA SMOL-TTCs showed fast clearance, potent antitumor efficacy, and high uptake and retention in prostate tumors in mice. No major uptake or retention in other organs was observed beyond kidneys. Low uptake of free thorium-227 into bone confirmed high complex stability in vivo. Salivary gland uptake remained inconclusive as mini pigs were devalidated as a relevant model and imaging controls failed in cynomolgus monkeys.

**Conclusion:**

Monomeric and multimeric PSMA SMOL-TTCs show high tumor uptake and fast clearance in preclinical models and warrant further therapeutic exploration.

**Supplementary Information:**

The online version contains supplementary material available at 10.1007/s00259-023-06474-z.

## Introduction

Prostate cancer (PrCa) is the second most frequently diagnosed cancer in men in developed countries [1]. Novel, efficient therapies are needed particularly for advanced PrCa. PrCa cells often specifically overexpress membrane-bound glutamate carboxypeptidase PSMA (prostate-specific membrane antigen) [2] which has become a well-established target antigen in PrCa pharmacology [3, 4]. Peptidomimetic inhibitors of the enzymatic functionality of PSMA can deliver conjugated cargo into PrCa cells [5]. PSMA-617 (vipivotide tetraxetan), which carries a DOTA (1,4,7,10-tetraazacyclododecane-1,4,7,10-tetraacetic acid) chelator and is labeled with the beta-emitting radionuclide lutetium-177 ([^177^Lu]PSMA-617, PLUVICTO^TM^), has shown clinical proof-of-concept in PrCa patients [6], and is approved by the United States Food and Drug Administration (FDA) for the treatment of patients with PSMA-positive metastatic castration-resistant PrCa who have been treated with androgen receptor pathway inhibition and taxane-based chemotherapy. There is, however, evidence that PSMA-targeted radionuclide therapy using alpha emitters that induce difficult-to-repair, clustered DNA double-strand breaks [7] may surpass the clinical efficacy of beta emitters. Indeed, the PSMA-targeting small molecule (SMOL) [^225^Ac]PSMA-617 has shown promising activity, including in patients refractory to [^177^Lu]PSMA-617 [8–10]. Furthermore, an alpha emitter radium-223 dichloride (Xofigo®) is approved by the FDA and European Medicines Agency (EMA) for patients with castration-resistant PrCa [11]. Unfortunately, the clinical use of [^225^Ac]PSMA-617 is hindered by xerostomia, which occurs in 63% of patients [12].

Besides actinium-225 and radium-223, thorium-227 is another attractive alpha-emitting radionuclide that is available in quantity and quality for clinical use. Thorium-227 decays by alpha-particle emission with an energy of 5.9 MeV and a half-life of 18.7 days to radium-223, with further decay releasing four alpha particles with a mean energy of 6.6 MeV and two beta particles, ending the cascade with the formation of stable lead-207 (Online Resource, Suppl. Fig. 1). Using thorium-227 as a fast-clearing small molecule will unfold therapeutic efficacy only if the molecule will remain in the tumor long enough to allow for the decay of thorium-227 and its daughters. Multivalency is one promising option for increasing tumor retention [13, 14] and adjusting biodistribution. Antibody-based targeted thorium-227 conjugates (TTC) have demonstrated promising preclinical antitumor activity [3, 15–18] and have entered early clinical testing (PSMA-TTC, BAY 2315497, [^227^Th]pelgifatamab corixetan: NCT03724747; HER2-TTC, BAY 2701439: NCT04147819).

Here, we developed PSMA SMOL-TTC, an optimized PSMA-targeting SMOL conjugate which shows robust labeling with the alpha emitter thorium-227, high uptake into PSMA-expressing tumors, and fast clearance from the circulation. The binding properties and antitumor activity of PSMA SMOL-TTC were evaluated in vitro and in vivo, including the effects of the multimerization of the conjugates. Moreover, biodistribution studies were performed in rodents, minipigs, and cynomolgus monkeys.

## Material and methods

All protocols are detailed in the Online Resource, Supplementary Methods.

### Compounds, cell lines, and tumor models

The PSMA SMOL conjugates were synthesized as described in Online Resource, Supplementary Methods. Thorium-227 was manufactured by IFE (Oslo, Norway). PSMA-617 was provided by ABX GmbH and 2-(phosphonomethyl)-pentanedioic acid (2-PMPA) by Sigma-Aldrich.

C4-2, 22Rv1, MDA-PCa-2b, and VCaP human PrCa cells were obtained from ATCC and LNCaP and PC-3 cells from DSMZ (Germany) between 2015 and 2018 and authenticated using short tandem repeat DNA fingerprinting at the Leibniz Institute (DSMZ).

The androgen-sensitive ST1273 [19] and the androgen-independent KUCaP-1 [20] patient-derived xenograft (PDX) PrCa models were obtained from START (San Antonio, TX, USA) and O. Ogawa (University of Kyoto, Kyoto, Japan), respectively.

### Generation of PSMA SMOL conjugates

**Fig. 1:**
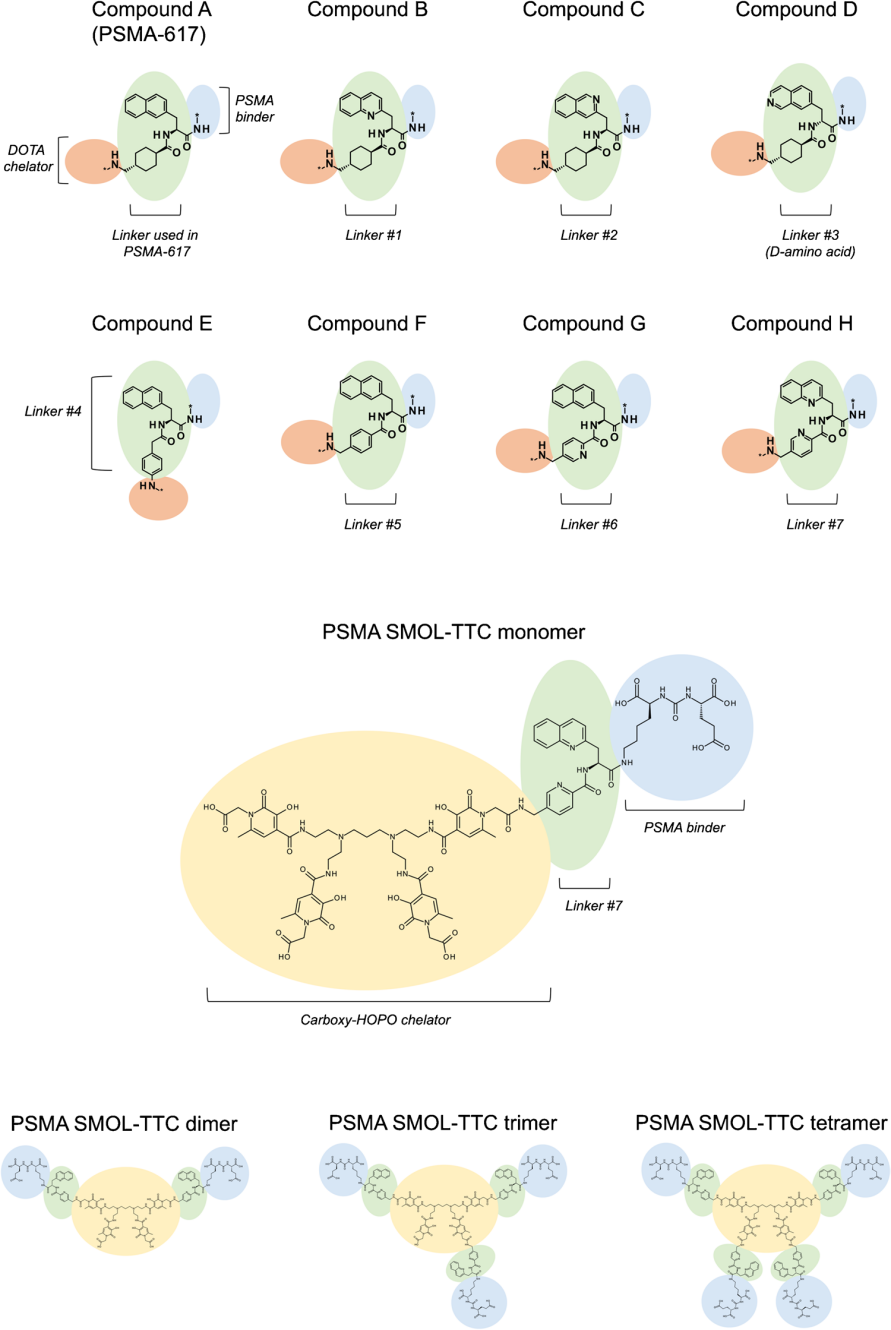
Chemical structures of PSMA-617 and the developed compounds

The PSMA SMOL conjugates were synthesized via amide-coupling of the carboxy-HOPO chelator moiety to the PSMA binder-linker motif (Online Resource, Suppl. Fig. 2). Seven linker candidates were synthesized with the aim of improving binding to PSMA (Fig. 1) as evaluated in a competition assay against [^3^H]PSMA-617 on PSMA-expressing LNCaP PrCa cells. In parallel, the HOPO chelator motif that allows for labeling with thorium-227 at room temperature and that has been used in antibody conjugate settings [3] was further modified. The resulting hydrophilic 3,2-hydroxypyridinone chelator bearing carboxyl groups (carboxy-HOPO) was employed in the synthesis of the PSMA SMOL conjugate.

The optimized PSMA SMOL conjugates were synthesized as monomeric, dimeric, trimeric, and tetrameric variants and labeled with thorium-227 to produce the final PSMA-targeting SMOL thorium-227 conjugates (PSMA SMOL-TTC)**.** The radiochemical purity of PSMA SMOL-TTCs was assessed by radio-HPLC. The specific activity of PSMA SMOL-TTCs was 375 kBq/nmol in all studies.

### In vitro characterization of PSMA SMOL-TTCs

In vitro characterization of PSMA SMOL-TTCs was performed by analyzing cytotoxicity, binding, internalization, and induction of apoptosis in PrCa cell lines. Stability of the compounds was investigated by incubating the compounds in citrate buffer, PBS (both at RT) or serum (at 37 °C), and aliquots were taken at indicated time points and analyzed by iTLC and HPLC.

### Antitumor efficacy and biodistribution of PSMA SMOL conjugates in vivo

The in vivo antitumor efficacy of PSMA SMOL-TTCs was assessed in the ST1273 and KUCaP-1 PDX models of human PrCa in mice. In the ST1273 model, female NMRI nude mice (*n*=10/group) were implanted with ST1273 tumor fragments and treated with a single dose (SD) of vehicle (0.9% NaCl) or monomer, dimer, trimer, or tetramer format of PSMA SMOL-TTC (1.5 MBq/kg, SD, i.v.). In the KUCaP-1 model, male SCID mice (*n*=10/group) were implanted with KUCaP-1 tumor fragments and treated with vehicle (0.9% NaCl; Q4W, i.v.), or monomer or dimer format of PSMA SMOL-TTC (1 or 2 MBq/kg, Q4Wx2, i.v.).

For evaluation of biodistribution by PET imaging, [^89^Zr]-labeled PSMA SMOLs were produced, and their purity and stability were investigated. The biodistribution of various PSMA SMOL conjugates was studied in ST1273 and LNCaP tumor-bearing mice, healthy minipigs, and healthy cynomolgus monkeys.

### Statistical analyses

The statistical analysis of tumor volumes was performed with SAS (version 9.2) using one-way and two-way ANOVA models. When analyzing the difference of groups over a defined time frame, a two-way repeated measure ANOVA was used. The model included the group effect and day effect as continuous variables, and the interaction term (group*day). When analyzing the difference of groups at a certain day, a one-way ANOVA was used. The model included the group effect. *p*-values for pairwise comparisons were adjusted using the Tukey method and considered statistically significant if *p*<0.05.

## Results

### Conjugate development

The conjugate resulting in PSMA SMOL-TTC was developed via the optimization of the linker and chelator motifs, using PSMA-617 as both a starting point and a reference compound (Fig. 1). First, the linker between the chelator and the PSMA binder was optimized by studying the PSMA binding of several non-radiolabeled newly developed DOTA-bearing PSMA SMOL conjugates with different linkers in a competition assay against ^3^H-PSMA-617 in LNCaP PrCa cells (Table 1). Exchanging the lipophilic naphthyl substituent in PSMA-617 (compound A) with a more hydrophilic quinolinyl analogue (compound B) increased the binding to PSMA. Furthermore, the position of the nitrogen atom in the quinolinyl moiety had an influence on binding. Compound B with the 2-quinolinyl moiety in the linker proved to be a better PSMA binder than compound C that had the nitrogen in the 3-position of the quinolinyl ring. Interestingly, compound D with a *D*-configuration in the linker amino acid also showed good binding to PSMA (12% at 10 nM; 66% at 100 nM; Table 1), although less than that of compounds A and B (9% and 45% at 10 nM; 78% and 88% at 100 nM, respectively). Exchange of the cyclohexyl moiety to an aromatic ring increased binding as seen with compound F. The position of the aromatic ring had a strong influence with the benzylamine configuration being better (see compound E vs. compound F). The hydrophilicity of the aromatic ring was increased via the introduction of a nitrogen atom, and the combination with the 2-quinolinyl moiety resulted in compound H with considerably improved PSMA binding against PSMA-617 (57 vs. 9% competition at 10 nM, and 94 vs. 78% at 100 nM) (Table 1). Thus, compound H was chosen for further development with optimization of the chelator moiety.

**Table 1:** Binding of PSMA SMOL conjugates to PSMA, presented as percentage of competition against 10 nM [^3^H]PSMA-617

		Conjugate concentration	
Compound	Chelator	10 nM	100 nM
PSMA-617	DOTA	9%	78%
PSMA-hydroxyethyl-HOPO	Hydroxyethyl-HOPO	29%	79%
*D*-Glu-PSMA-617	DOTA	20%	22%
Compound B	DOTA	45%	88%
Compound C	DOTA	8%	71%
Compound D	DOTA	12%	66%
Compound E	DOTA	34%	82%
Compound F	DOTA	41%	94%
Compound G	DOTA	46%	95%
Compound H	DOTA	57%	94%
PSMA SMOL conjugate, monomer	Carboxy-HOPO	42%	90%
PSMA SMOL conjugate, dimer	Carboxy-HOPO	70%	95%
PSMA SMOL conjugate, trimer	Carboxy-HOPO	67%	95%
PSMA SMOL conjugate, tetramer	Carboxy-HOPO	27%	86%

Since the DOTA chelator complexes with thorium-227 only at elevated temperatures [21], the hydroxyethyl-HOPO chelator which has been previously conjugated with antibodies and readily complexes with thorium-227 at room temperature [22] was chosen instead (Online Resource, Suppl. Fig. 2). A PSMA-targeting SMOL using this chelator showed similar in vitro binding properties as PSMA-617 (Table 1) and robust thorium-227 labeling with 100 % labeling efficiency by iTLC. However, when tested in vivo, the tumor uptake was lower than [^227^Th]PSMA-617 and unspecific uptake remained high (data not shown). Therefore, to improve the hydrophilicity of the chelator, it was modified with carboxyl groups resulting in a new 3,2-hydroxypyridinone chelator (carboxy-HOPO). Combining the carboxy-HOPO chelator with the optimized linker from compound H, a PSMA SMOL conjugate monomer showing comparable binding to compound H bearing a DOTA-chelator was obtained (Table 1). The newly developed carboxy-HOPO contained four attachment points via its carboxyl groups, enabling the synthesis of multimeric variants (dimer, trimer, tetramer) of the PSMA SMOL conjugate in addition to the monomeric variant (Fig. 1 and Online Resource, Suppl. Fig. 3) with PSMA-binding properties comparable to compound H (Table 1). The radiochemical purity of all PSMA SMOL-TTC conjugates labeled with thorium-227 was at least 95%, confirming that the carboxy-HOPO is highly stable at room temperature (Online Resource, Suppl. Fig. 4a–c). For the dimer, two constitutional isomers (1,1 and 1,2) could theoretically exist due to the synthesis of statistical conjugation of the carboxy-HOPO with the linker amine. As we were not able to analytically discriminate nor separate these two theoretical isomers, the dimer should be considered a potential mixture of the 1,1- and 1,2-isomers (Online Resource, Suppl. Fig. 5).

The structures of PSMA-617, compounds B–H, and the PSMA SMOL conjugates are presented in Fig. 1 and Online Resource, Suppl. Fig. 3, and the structure of PSMA-hydroxyethyl-HOPO is presented in Online Resource, Suppl. Fig. 2*Carboxy-HOPO*, 2,3-hydroxypyridinone; *DOTA*, dodecane tetraacetic acid.

The seven newly developed linker alternatives were investigated as part of conjugates containing the PSMA binder and a DOTA chelate (compounds B–H). The mono- and multimeric PSMA SMOL-TTCs consist of 1–4 PSMA SMOL conjugates coupled with a carboxy-HOPO chelate via the optimized linker from compound H. The solid circles indicate the PSMA binder moiety (blue), the variable linker moiety (green), the carboxy-HOPO chelate (yellow), and the DOTA chelate (orange).

### The cytotoxicity of PSMA SMOL-TTC results from binding and internalization into PSMA-positive cells

**Fig. 2:**
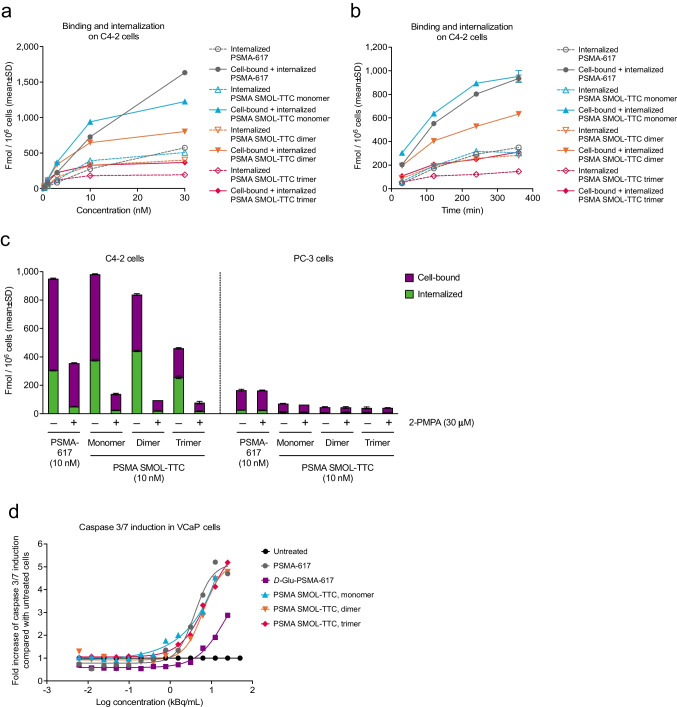
Binding and internalization of [^227^Th]-labeled PSMA SMOL conjugates (PSMA SMOL-TTCs) in vitro. **a** Binding and internalization on C4-2 cells at various concentrations of the conjugates after a 2-h incubation. **b** Binding and internalization of the conjugates (10 nM) on C4-2 cells over time. **c** Binding and internalization of the conjugates on PSMA-expressing C4-2 cells and PSMA-negative PC-3 cells in the presence of 2-PMPA (30 nM) after a 2-h incubation. **d** Induction of caspase 3/7 by PSMA SMOL conjugates in VCaP cells after a 3-day incubation. The conjugates were used at a specific radioactivity of 375 kBq/nmol. The dose-response curves were plotted using a four-parameter logistic equation

Radiolabeled PSMA SMOL-TTC variants were shown to bind and internalize into PSMA-positive PrCa cells (Fig. 2a–b and Online Resource, Suppl. Fig. 6). All tested variants showed concentration- and time-dependent binding and internalization into C4-2 cells with the saturation point being reached after 4 h regardless of molecule size (Fig. 2a–b). After a 4-h incubation with PSMA SMOL-TTC variants at 10 nM, 14, 16, 8, and 4 % cell-bound radioactivity (of total activity) and 6, 7, 7, and 4 % of internalized radioactivity were detected for PSMA-617 and the PSMA SMOL-TTC monomer, dimer, and trimer, respectively (Fig. 2b). Thus, only 8–23% of the total radioactivity was cell-bound or internalized and 77–92% of the total radioactivity remained still in the supernatant. This suggested saturation of the cellular binding and internalization capacities for the tested variants after a 4-h of incubation at 10 nM. The monomer showed the fastest and strongest cellular binding out of the studied PSMA SMOL-TTC variants. The dimer showed lower cell binding, but similar internalization compared with the monomer, whereas total binding and internalization of the trimer was found to be lowest in the PSMA-positive cell lines. Binding and internalization of the tetramer was similar to the dimer in LNCaP cells.

The simultaneous use of the competitor compound 2-PMPA strongly reduced binding and internalization of the investigated PSMA SMOL-TTC variants up to 90 %. Binding and internalization into PSMA-negative PC-3 cells were low (Fig. 2c). All tested PSMA SMOL-TTC variants induced apoptosis as evidenced by increased caspase 3/7 levels in VCaP PrCa cells (Fig. 2d). The cytotoxic effect of PSMA SMOL-TTCs occurred in relation to PSMA expression and was similar to [^227^Th]PSMA-617 (Table 2). The low-binding [^227^Th]*D*-Glu-PSMA-617 showed lower cytotoxicity than [^227^Th]PSMA-617. PSMA-negative PC-3 cells were largely unaffected by all tested PSMA-targeting compounds.

**Table 2:** Cytotoxicity of the PSMA SMOL-TTC multimers and PSMA expression levels in various prostate cancer cell lines

Cell line	LNCaP	C4-2	MDA-PCa-2b	VCaP	22Rv1	PC-3
PSMA antibodies bound/cell^a^	160,000	171,000	77,000	22.000	2000	0
PSMA SMOL conjugate	Cytotoxicity(IC_50_,kBq/mL)					
[^227^Th]PSMA-617	0.04	0.19	0.013	0.42	0.71	4.5
[^227^Th]PSMA-hydroxyethyl-HOPO	0.67	2.14	N/A	3.07	1.06	N/A
[^227^Th]*D*-Glu-PSMA-617	0.60	3.18	0.315	4.74	1.28	7.1
PSMA SMOL-TTC, monomer	0.02	0.17	0.012	0.97	1.28	10.2
PSMA SMOL-TTC, dimer	0.02	0.46	0.011	1.25	1.36	9.9
PSMA SMOL-TTC, trimer	0.03	1.55	0.013	1.96	1.34	11.2
PSMA SMOL-TTC, tetramer	0.02	0.28	N/A	1.55	1.25	N/A

^a^Determined by flow cytometry [3, 15]

All PSMA SMOL-TTCs demonstrated good stability in PBS for up to 48 h but were unstable in citrate buffer (Online Resource, Suppl. Table 1). Furthermore, the PSMA SMOL-TTC monomer was stable in both human and mouse serum for up to 60 h (Online Resource, Suppl. Fig. 7).

### PSMA SMOL-TTC displays high and targeted tumor uptake and in vivo efficacy

**Fig. 3:**
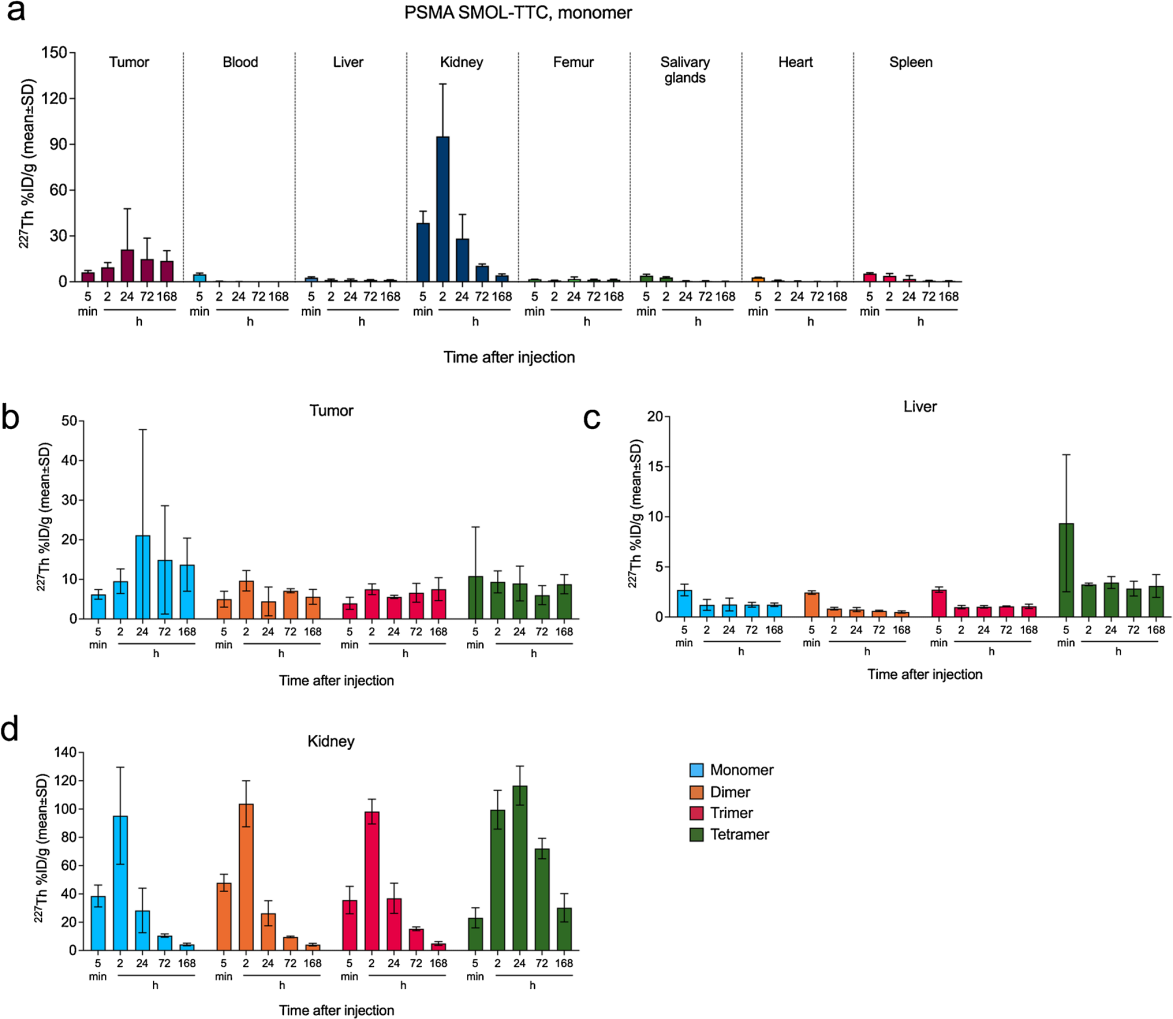
Biodistribution of PSMA SMOL-TTCs in ST1273 PDX tumor-bearing mice Thorium-227 activity for **a** monomer in all studied organs, and for all variants in **b** tumor, **c** liver, and **d** kidneys at 5 min, 2 h, 24 h, 72 h, and 168 h after PSMA SMOL-TTC injection (1.5 MBq/kg, i.v.)

PSMA SMOL-TTC variants demonstrated high tumor uptake of approximately 10% ID/g (Fig. 3a–b). Monomeric, dimeric, and trimeric PSMA SMOL-TTCs showed no major uptake into other organs and were cleared by the 24-h time point primarily by renal excretion (Fig. 3c–d and Online Resource, Suppl. Fig. 8). For the tetrameric PSMA SMOL-TTC, clearance from the kidneys, salivary glands, and spleen was slower than the other multimers with higher uptake and retention in the liver. The bone uptake of thorium-227 was 1.5–2% ID/g for all PSMA SMOL-TTC variants. These results were confirmed by imaging [^89^Zr]-labeled PSMA SMOL monomer, dimer, and trimer by PET in an LnCap mouse xenograft model (see Online Resource, Suppl. Fig. 9).

**Fig. 4:**
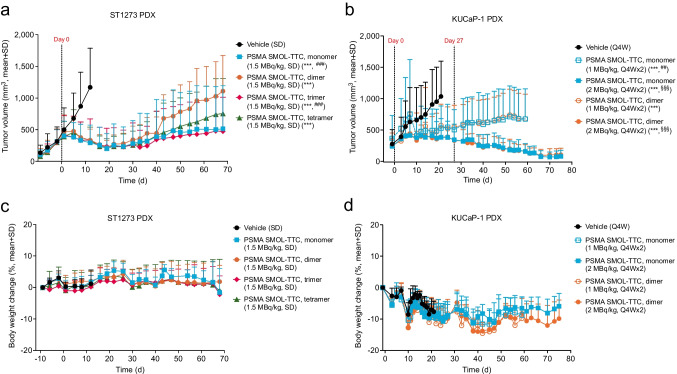
Efficacy of PSMA SMOL-TTCs in PrCa PDX models in mice. **a** Growth curves of ST1273 PDX tumors in mice treated with vehicle (SD, i.v.) or PSMA SMOL-TTC variants (1.5 MBq/kg, SD, i.v.). **b** Growth curves of KUCaP-1 PDX tumors in mice treated with vehicle (Q4W, i.v.) or PSMA SMOL-TTC monomer or dimer (1 or 2 MBg/kg, Q4Wx2, i.v.). **c** Body weight change of ST1273 tumor-bearing mice described in **a**. **d** Body weight change of KUCaP-1 tumor-bearing mice described in **b**. The administration days are indicated with vertical dotted lines. Tumor volume graphs were generated with last measured tumor values carried forward, but statistical analyses were performed with the actual values for each day using a one-way ANOVA model at one time point or a two-way ANOVA model over a defined time period. The individual tumor growth curves are shown in Online Resource, Suppl. Fig. 10. ***, *p*<0.001 compared with vehicle on day 12 (ST1273) or over all time points until day 21 (KUCaP-1). ^##^, *p*<0.01; ^###^, *p*<0.001 compared with the similarly dosed dimer group on day 68 (ST1273) or over all time points until day 59 (KUCaP-1). ^§§§^, *p*<0.001 compared with the lower 1 MBq/kg dose group of the same PSMA SMOL-TTC multimer format over all time points until day 21 (KUCaP-1)

In the ST1273 model, PSMA SMOL-TTC variants (1.5 MBq/kg, SD, i.v.) resulted in a marked reduction (*p*<0.001) in tumor volume compared with vehicle on day 12 with T/C values of 0.27, 0.31, 0.31, and 0.34 for the monomer, dimer, trimer, and tetramer, respectively (Fig. 4a and Online Resource, Suppl. Fig. 10a–e). In the KUCaP-1 model, PSMA SMOL-TTC (1 MBq/kg, Q4Wx2, i.v.) as a monomer and a dimer (T/C 0.61 and 0.66, respectively on day 21) resulted in markedly (*p*<0.001) reduced tumor volume compared with vehicle over all time points (Fig. 4b and Online Resource, Suppl. Fig. 10f–j). Doubling of the PSMA SMOL-TTC dose to 2 MBq/kg resulted in stronger efficacy indicating dose-dependency (T/C 0.44, *p*<0.001, for both variants on day 21). PSMA SMOL-TTC treatment was well tolerated in both models. Tumor-induced body weight loss was observed in the vehicle group in the KUCaP-1 model, whereas the absence of body weight loss and stabilization of body weight observed in the treatment groups can, therefore, be considered as a proof of efficacy and well tolerated therapy (Fig. 4c–d).

### PSMA SMOL conjugates show rapid kidney clearance in mice, minipigs, and monkeys

**Fig. 5:**
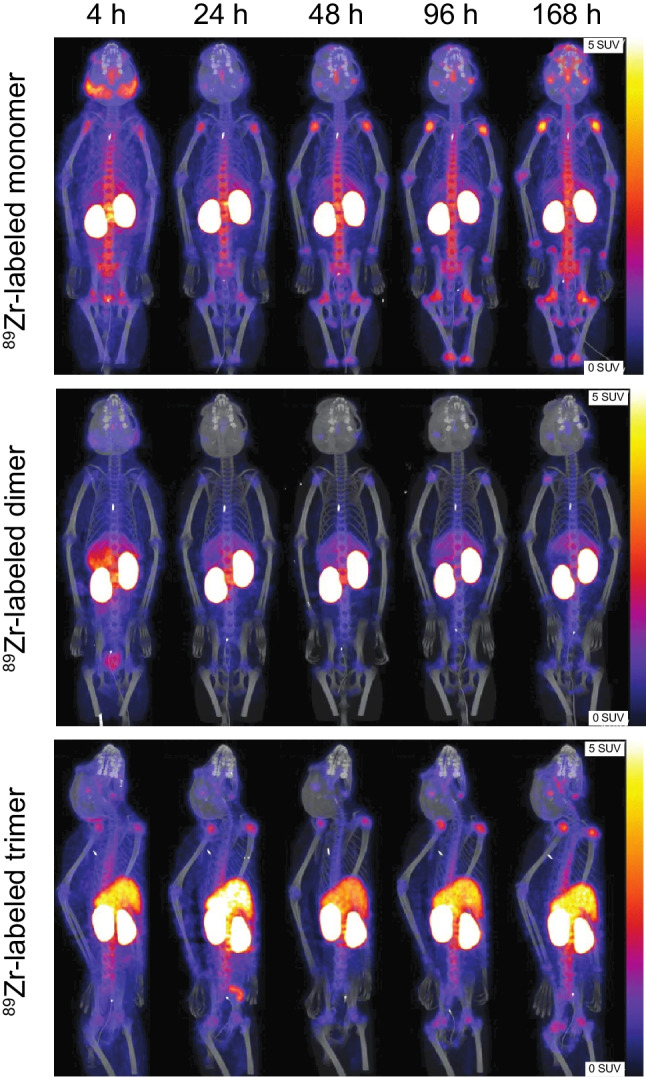
Biodistribution of [^89^Zr]-labeled PSMA SMOL conjugates by PET imaging in cynomolgus monkeys. Representative images of the biodistribution of [^89^Zr]-labeled monomer, dimer, and trimer PSMA SMOL conjugates (48.1×10^6^, 70.3×10^6^, and 44.4×10^6^ Bq, respectively, i.v.) in cynomolgus monkeys 4, 24, 48, 96, and 168 h after injection (*n*=2/group). SUV, standardized uptake value

The biodistribution of PSMA SMOL conjugates was also assessed by imaging in various animal species with special attention to salivary gland as a potential target organ of toxicity. [^89^Zr]-labeled PSMA SMOL conjugates produced for PET imaging demonstrated good stability in monkey serum (Online Resource, Suppl. Table 2). In cynomolgus monkeys (non-tumor-bearing animals), the [^89^Zr]-labeled monomer and dimer PSMA SMOL conjugates showed rapid clearance (Fig. 5). For all compounds, accumulation was observed in the kidneys and only the trimer showed accumulation in the liver. Similar kidney and liver retention was not observed in LNCaP tumor-bearing mice (Online Resource, Suppl. Fig. 9). The joints showed over time increasing uptake of zirconium-89, particularly for the monomer and the trimer. In the salivary gland, only a low and transient uptake of only the monomer was detected in cynomolgus monkeys. However, the data on salivary gland uptake are not conclusive due to the lack of a PSMA-617 positive control in the study. Minipigs were also investigated as a potential model, but no accumulation in salivary gland was observed in them (Online Resource, Suppl. Fig. 11).

## Discussion

Here, we developed PSMA SMOL-TTC, a novel PSMA-targeting SMOL conjugate suitable for labeling with thorium-227 with the aim of improving on the characteristics of PSMA-617 radioligand conjugates, including reducing salivary gland and kidney uptake. PSMA SMOL-TTC is the first SMOL with thorium-227 and it features an optimized linker motif and a modified carboxy-HOPO chelator that improves hydrophilicity. The developed linker-chelator combination provides favorable binding properties, and it can be complexed with thorium-227 at room temperature, unlike conjugates like PSMA-617 that feature DOTA chelators that require heating for the labeling process [21]. The option of producing multimeric PSMA SMOL conjugates offers the possibility of further adjusting the pharmacological properties of PSMA SMOL-TTC, such as tumor uptake rate, salivary gland uptake, and pharmacokinetic profile.

The PSMA SMOL conjugates demonstrated strong ability to compete against the [^3^H]PSMA-617 reference compound in a competition assay in PSMA-expressing PrCa cells. Additionally, PSMA SMOL-TTC and its multimeric forms showed rapid and concentration-dependent internalization into PSMA-expressing PrCa cells in vitro. Interestingly, the binding and internalization of the tested PSMA SMOL-TTC variants was almost completely negated in PSMA-negative PC-3 cells, and in C4-2 cells when incubated together with the PSMA-specific inhibitor 2-PMPA, indicating high specificity.

The development of highly specific PSMA-targeting SMOL ligands is of paramount importance in minimizing their adverse effects and in broadening their clinical use. Previous research has shown that despite the encouraging clinical response rates experienced with [^225^Ac]PSMA-617 in investigational trials [23], strong uptake into the salivary glands becomes a dose-limiting toxicity that leads to severe xerostomia [9]. Given the low-to-moderate expression of PSMA in the salivary gland and the low salivary gland uptake of PSMA-targeting antibody conjugates [9, 24, 25], this accumulation is thought to be at least partially non-specific and could potentially also be transporter-related. Our results indicate that the developed PSMA SMOL-TTC with optimized linker and chelator chemistry presents little non-specific binding and very good internalization into PSMA-positive cells. A lowered potential for xerostomia induction may be further suggested by the size and charge difference between the PSMA-617 and the PSMA SMOL-TTC variants. This could lead to a discriminatory effect on the recognition of potential transporters in the salivary glands between the two compounds. Indeed, all PSMA SMOL-TTC variants showed only little and transient uptake into murine salivary glands. As minipig salivary glands have been used in the literature to investigate salivary gland uptake with reports suggesting that they could be a relevant model [26], and the salivary gland tissues from non-human primates have exhibited comparable PSMA expression to human salivary glands [27], minipigs and cynomolgus monkey were considered as relevant models. Unfortunately, no salivary gland accumulation of [^227^Th]-labeled PSMA-617 was observed in minipigs, and thus, minipigs were proven to be an unsuitable model. The assessment of salivary gland uptake in cynomolgus monkey could not be validated due to the inability to use [^89^Zr]-labeled PSMA-617 as positive control. We also tested [^64^Cu]-labeled PSMA-617 in cynomolgus monkeys but no salivary gland uptake was seen with this compound either, and the overall poor image quality suggested fast decomplexing of copper-64 from the chelator. Therefore, we were unfortunately not able to draw any conclusions on the salivary gland uptake of the PSMA SMOL-TTCs. Thus, the favorable biodistribution of novel PSMA-targeting SMOLs needs to be addressed more thoroughly in clinical trials due to the lack of good animal models representing salivary gland toxicity. The uptake of [^89^Zr]-labeled compounds into the bones and joints is most likely due to decomplexation of zirconium-89 from the chelator, as 80% of the zirconium-89 was observed to be associated with the compound after addition to monkey serum at 37 °C in vitro, and further decomposition was observed over time (Suppl. Table 2)*.* Since the chelator was developed primarily for therapeutic applications of thorium-227, its use for zirconium-89 imaging is actually a secondary application. We, however, believe that zirconium-89 imaging is useful for general compound development. The timeframes of imaging studies are much shorter than those of therapeutic applications, and therefore, the stability of [^89^Zr]-labeled compounds is not expected to play a role the imaging studies.

The clearance of the compounds occurred via the kidneys for the monomeric, dimeric, and trimeric forms of PSMA SMOL-TTC. The clearance in mice was rapid, whereas more retention was observed in the kidneys of cynomolgus monkeys with over half of the maximal activity still present after 1 week. A possible explanation could be the higher PSMA expression in the kidneys of cynomolgus monkeys [27]. Importantly, the specificity of PSMA SMOL-TTC also translates into good tolerability in vivo, as no animals in either the ST1273 or the KUCaP-1 PrCa PDX model displayed major decreases in body weight that would necessitate cessation of treatment.

The linker and chelator optimization of PSMA SMOL-TTC also leads to favorable efficacy both in vitro and in vivo compared with the known hydroxyethyl-HOPO chelator. In PrCa cells with varying PSMA expression levels, the cytotoxicity of monomeric, dimeric, and trimeric PSMA SMOL-TTC increased in proportion to PSMA expression. In the ST1273 model, monomeric, dimeric, and trimeric PSMA SMOL-TTC showed strong antitumor efficacy. Similar results were also seen in KUCaP-1 tumors. Significant liver uptake of the tetrameric variant made it a less desirable option for the clinic, but as the other three variants showed favorable efficacy, further studies are warranted to identify the compound with the optimal biodistribution pattern. Even though [^89^Zr]-labeled compounds are not suitable for diagnostic purposes due to their short half-lives, they could be a valuable tool in understanding the pharmacokinetics and biodistribution of such conjugates in the clinical setting.

Comparing the effects of multimerization in vitro, subtle differences between the monomer, dimer, trimer, and tetramer were observed, which we could attribute to multimerization. With respect to cellular binding and internalization of the radiolabeled compounds, the monomer was clearly the best-performing compound in vitro. Compared with the monomer, the dimer showed stronger displacement of [^3^H]PSMA-617 in the competition assay and similar internalization, yet the dimer displayed overall weaker cellular binding. The trimer showed poorer binding and internalization than monomer and dimer, whereas the tetramer showed diminished competition with [^3^H]PSMA-617 but similar binding and internalization compared with the dimer. In vivo, the monomer and dimer performed quite similarly to each other with respect to efficacy and tumor accumulation, but with regard to elimination behavior, the dimer showed intriguingly fast clearance in the monkeys, with kidneys as the only organ of residual activity. We detected only very little uptake of the dimer into the joints and vertebral column. A possible explanation for this could be the conformation of the dimer structure. The binding regions of the dimer are directly attached to the chelator, and this could stabilize the zirconium-89 in the dimer-chelator resulting in decreased levels of zirconium-89 leaking out of the chelator into the joints. Moreover, the dimer showed better systemic clearance, resulting in lower plasma concentrations, and consequently, less decomplexation of zirconium-89 from the chelator. In contrast, the monomer and trimer seemed to slightly lose zirconium-89 radionuclides out of their chelator when accumulating in the bones and joints. Notably, the larger size of the trimer and tetramer led to their increase in liver accumulation. The observed liver uptake of the trimer could be explained by the multimerization, since also the [^227^Th]-labeled tetramer has shown liver uptake in mice. This effect may be more pronounced with [^89^Zr]-labeled compounds or depend on the species investigated, which could explain the observed higher liver uptake in monkeys compared with mice. However, further studies are needed to elucidate the mechanisms behind the liver uptake patterns of the different multimers. Overall, multivalency did not improve the proven tumor uptake or efficacy, but rather provided an opportunity to improve on other compound properties such as elimination behavior or accumulation in salivary glands.

Taken together, monomeric and dimeric forms of PSMA SMOL-TTC showed the best tumor-to-background ratios and lowest amount of kidney accumulation. Based on these results, further evaluation of mono- and dimeric conjugates in the clinical setting would be warranted. In humans, the biodistribution of PSMA SMOL-TTCs could be evaluated for example by gamma camera imaging of thorium-227, and eventually also its daughter nuclide radium-223, or PET imaging with the [^89^Zr]-labeled compounds.

In summary, the newly developed conjugates demonstrate target-specific uptake and fast clearance resulting in efficacy in PSMA-expressing PrCa cells and xenograft models with good tolerability. Taken together, the presented data highlight the potential of treating PrCa with a PSMA-targeting SMOL-thorium-227 conjugate.

### Supplementary Information

Below is the link to the electronic supplementary material.

Supplementary file1 (DOCX 4979 KB)

Supplementary file2 (DOCX 9890 KB)

## Data Availability

The datasets generated during an/or analyzed during the current study are available from the corresponding author on reasonable request.

## References

[1] Bray F, Ferlay J, Soerjomataram I, Siegel RL, Torre LA, Jemal A (2018). Global cancer statistics 2018: GLOBOCAN estimates of incidence and mortality worldwide for 36 cancers in 185 countries. CA Cancer J Clin..

[2] Hyvakka A, Virtanen V, Kemppainen J, Gronroos TJ, Minn H, Sundvall M (2021). More than meets the eye: scientific rationale behind molecular imaging and therapeutic targeting of prostate-specific membrane antigen (PSMA) in metastatic prostate cancer and beyond. Cancers (Basel)..

[3] Hammer S, Hagemann UB, Zitzmann-Kolbe S, Larsen A, Ellingsen C, Geraudie S (2020). Preclinical efficacy of a PSMA-targeted thorium-227 conjugate (PSMA-TTC), a targeted alpha therapy for prostate cancer. Clin Cancer Res..

[4] Silver DA, Pellicer I, Fair WR, Heston WD, Cordon-Cardo C (1997). Prostate-specific membrane antigen expression in normal and malignant human tissues. Clin Cancer Res..

[5] Will L, Sonni I, Kopka K, Kratochwil C, Giesel FL, Haberkorn U (2017). Radiolabeled prostate-specific membrane antigen small-molecule inhibitors. Q J Nucl Med Mol Imaging..

[6] Sartor O, de Bono J, Chi KN, Fizazi K, Herrmann K, Rahbar K (2021). Lutetium-177-PSMA-617 for metastatic castration-resistant prostate cancer. N Engl J Med..

[7] Hagemann UB, Wickstroem K, Hammer S, Bjerke RM, Zitzmann-Kolbe S, Ryan OB (2020). Advances in precision oncology: targeted thorium-227 conjugates as a new modality in targeted alpha therapy. Cancer Biother Radiopharm..

[8] Feuerecker B, Tauber R, Knorr K, Heck M, Beheshti A, Seidl C (2021). Activity and adverse events of actinium-225-PSMA-617 in advanced metastatic castration-resistant prostate cancer after failure of lutetium-177-PSMA. Eur Urol..

[9] Kratochwil C, Bruchertseifer F, Rathke H, Hohenfellner M, Giesel FL, Haberkorn U (2018). Targeted alpha-therapy of metastatic castration-resistant prostate cancer with (225)Ac-PSMA-617: swimmer-plot analysis suggests efficacy regarding duration of tumor control. J Nucl Med..

[10] Sathekge M, Bruchertseifer F, Vorster M, Lawal IO, Mokoala K, Reed J (2023). (225)Ac-PSMA-617 radioligand therapy of de novo metastatic hormone-sensitive prostate carcinoma (mHSPC): preliminary clinical findings. Eur J Nucl Med Mol Imaging..

[11] Sartor O, Coleman R, Nilsson S, Heinrich D, Helle SI, O’Sullivan JM (2014). Effect of radium-223 dichloride on symptomatic skeletal events in patients with castration-resistant prostate cancer and bone metastases: results from a phase 3, double-blind, randomised trial. Lancet Oncol..

[12] Lee DY, Kim YI (2022). Effects of (225)Ac-labeled prostate-specific membrane antigen radioligand therapy in metastatic castration-resistant prostate cancer: a meta-analysis. J Nucl Med..

[13] Zhao L, Niu B, Fang J, Pang Y, Li S, Xie C (2022). Synthesis, preclinical evaluation, and a pilot clinical PET imaging study of (68)Ga-labeled FAPI dimer. J Nucl Med..

[14] Wurzer A, Pollmann J, Schmidt A, Reich D, Wester HJ, Notni J (2018). Molar activity of Ga-68 labeled PSMA inhibitor conjugates determines PET imaging results. Mol Pharm..

[15] Hammer S, Schlicker A, Zitzmann-Kolbe S, Baumgart S, Hagemann UB, Scholz A (2021). Darolutamide potentiates the antitumor efficacy of a PSMA-targeted thorium-227 conjugate by a dual mode of action in prostate cancer models. Clin Cancer Res..

[16] Lejeune P, Cruciani V, Berg-Larsen A, Schlicker A, Mobergslien A, Bartnitzky L (2021). Immunostimulatory effects of targeted thorium-227 conjugates as single agent and in combination with anti-PD-L1 therapy. J Immunother Cancer..

[17] Schatz CA, Hagemann U, Zitzmann-Kolbe S, Haendler B, Hennekes H, Hammer S (2022). Darolutamide potentiates the antitumor efficacy of a PSMA-targeted thorium-227 conjugate (PSMA-TTC) in a hormone-independent prostate cancer model. Cancer Res..

[18] Schatz CA, Suominen MI, Knuuttila M, Zitzmann-Kolbe S, Rissanen J, Käkönen SM (2022). PSMA-targeted thorium-227 conjugate (PSMA-TTC) inhibits tumor growth and abnormal bone changes in the intratibial LNCaP xenograft model of bone-metastatic prostate cancer. Cancer Res..

[19] Wick M, Quinn M, Mangold A, Gamez L, Diaz A, Vaught T (2016). Establishment and characterization of a hormone dependent, PSA/PSMA positive prostate PDX model. Eur J Cancer..

[20] Yoshida T, Kinoshita H, Segawa T, Nakamura E, Inoue T, Shimizu Y (2005). Antiandrogen bicalutamide promotes tumor growth in a novel androgen-dependent prostate cancer xenograft model derived from a bicalutamide-treated patient. Cancer Res..

[21] Heyerdahl H, Krogh C, Borrebaek J, Larsen A, Dahle J (2011). Treatment of HER2-expressing breast cancer and ovarian cancer cells with alpha particle-emitting 227Th-trastuzumab. Int J Radiat Oncol Biol Phys..

[22] Bonge-Hansen HT, Ryan OB. Radio-pharmaceutical complexes. WO 2013;167756

[23] Sathekge MM, Bruchertseifer F, Vorster M, Morgenstern A, Lawal IO (2021). Global experience with PSMA-based alpha therapy in prostate cancer. Eur J Nucl Med Mol Imaging..

[24] Rupp NJ, Umbricht CA, Pizzuto DA, Lenggenhager D, Topfer A, Muller J (2019). First clinicopathologic evidence of a non-PSMA-related uptake mechanism for (68)Ga-PSMA-11 in salivary glands. J Nucl Med..

[25] Tagawa ST, Milowsky MI, Morris M, Vallabhajosula S, Christos P, Akhtar NH (2013). Phase II study of Lutetium-177-labeled anti-prostate-specific membrane antigen monoclonal antibody J591 for metastatic castration-resistant prostate cancer. Clin Cancer Res..

[26] Tonnesmann R, Meyer PT, Eder M, Baranski AC (2019). [(177)Lu]Lu-PSMA-617 salivary gland uptake characterized by quantitative in vitro autoradiography. Pharmaceuticals (Basel)..

[27] Roy J, Warner BM, Basuli F, Zhang X, Wong K, Pranzatelli T (2020). Comparison of prostate-specific membrane antigen expression levels in human salivary glands to non-human primates and rodents. Cancer Biother Radiopharm..

