# Supplementary material for: Mono- and multimeric PSMA-targeting small molecule-thorium-227 conjugates for optimized efficacy and biodistribution in preclinical models
Source: Eur J Nucl Med Mol Imaging. 2023 Oct 26;51(3):669–80. doi: 10.1007/s00259-023-06474-z (PMC10796422; doi:10.1007/s00259-023-06474-z)
Supplement: Supplementary file 1 — Supplementary file1 (DOCX 4979 KB) [file 259_2023_6474_MOESM1_ESM.docx]

**Supplementary materials and methods for:**

**Mono- and multimeric PSMA-targeting small molecule-thorium-227 conjugates for optimized efficacy and biodistribution in preclinical models**

***European Journal of Nuclear Medicine and Molecular Imaging***

Niels Böhnke^1^, Bård Indrevoll^2^, Stefanie Hammer^1^, Alex Papple^2^, Alexander Kristian^2^, Hans Briem^1^, Arif Celik^1^, Dominik Mumberg^1,3^, Alan Cuthbertson^2^, Sabine Zitzmann-Kolbe^1^

^1^Bayer AG, Pharmaceuticals, Berlin, Germany;

^2^Bayer AS, Oslo, Norway

^3^Current address: Adcendo ApS, Copenhagen, Denmark

**Corresponding author:** Sabine Zitzmann-Kolbe, Bayer AG, Research & Early Development, Pharmaceuticals, 13342 Berlin, Germany. E-mail: sabine.zitzmann-kolbe@bayer.com

**SUPPLEMENTARY METHODS**

**Conjugate development**

**Radiolabeling with thorium-227 and zirconium-89, quality control, and radioactivity detection**

The monomeric, dimeric, trimeric, and tetrameric variants of the optimized PSMA SMOL conjugates were labeled with thorium-227 in TRIS/citrate buffer (40 mM TRIS, 6 mM citrate, 2 mM EDTA, 10 mg/mL ascorbate, 50 mg/mL sucrose, pH 7) to produce the final PSMA-targeting small molecule thorium-227 conjugates (PSMA SMOL-TTCs). Purified thorium-227 in 0.05 M HCl was added to 0.02 mM solutions of the conjugates at a radioactive concentration of 5 MBq/mL. The labeling mixtures were incubated at room temperature for 90 min, followed by dilution with the citrate buffer described above. The radiochemical purity (RCP) of PSMA SMOL-TTCs was assessed with radio-HPLC and iTLC.

PSMA-617 and *D*-Glu-PSMA-617 (both at 0.02 mM) carrying DOTA chelates were labeled with thorium-227 in 0.1 M acetate (pH 5) at 90 ºC for 45 min.

PSMA-617-hydroxyethyl-HOPO, 0.02 mM in citrate buffer (30 mM citrate, 2 mM EDTA, 2 mg/mL PABA, 50 mg/mL sucrose, pH 5.5), was labeled with thorium-227 at room temperature for 60 min at a radioactive concentration of 3.7 MBq/mL.

The RCP of the ^227^Th-PSMA-617 compounds was assessed by instant thin-layer chromatography (iTLC) where RCP is defined as the fraction of bound thorium-227 compared to free thorium-227, quantified using a HPGe detector.

Radio-HPLC system consisted of a Vanquish UHPLC from Thermo Fisher connected to a Berthold FlowStar² LB514 radio detector equipped with a BGO gamma-ray absorber. A Phenomenex Luna C5 column (5 um 150 x 2 mm) was used with eluents A (100 mM acetate, 30 mm citrate, pH 6) and B (acetonitrile, flow: 0.5 mL/min; temperature: 40 °C). A gradient of 5–30% B over 10 min was used for the monomer and trimer, whereas a gradient of 5–50% B over 10 min was used for the dimer.

Measurements of thorium-227 were performed by gamma spectroscopic measurement using a Germanium detector (Mirion). As each alpha decay emits specific energy lines, the decay can be assigned to thorium-227, radium-223 or any of the daughters. Therefore, the data from thorium-227 measurements experience no interference from radium-223 or daughters. Decay corrections were carried out by the software to the appropriate assay time point or sectioning time point.

The monomeric, dimeric, and trimeric variants of the optimized PSMA SMOL conjugates were also separately labeled with zirconium-89 in HEPES buffer (1 M HEPES pH 8, 0.02% Tween-20) for the biodistribution and imaging studies. Purified Zr(Ox)_2_ (#NEZ308000MC, PerkinElmer) was added to solutions of the conjugates and the labeling mixtures were incubated at room temperature for 90 min, followed by purification with Waters Sep-Pak tC18 Plus Light Cartridge (WAT036805, Waters). The RCP of [^89^Zr]PSMA SMOL conjugates was assessed with radio-HPLC and iTLC, where HPLC yielded >99% RCP but iTLC was not able to distinguish [^89^Zr]PSMA SMOL conjugates from free [^89^Zr]. The purity of ^89^Zr-labeled PSMA SMOLs as determined by HPLC using an in-line radiation detector on the day of production is shown below.

**Competition assay of PSMA SMOL conjugates**

The effect of the linkers on the binding of the resulting PSMA SMOL conjugates was assessed in a competition assay against ^3^H-PSMA-617 on PSMA-expressing LNCaP prostate cancer cells. Conjugates were used at 10 or 100 nM concentrations and ^3^H-PSMA-617 at 10 nM. LNCaP cells (7.5x10^5^) were seeded in 500 μL growth medium [RPMI1640 (Biochrom #FG1215), 10% fetal calf serum (FCS Superior; Biochrom #S0615)] in 24-well plates (#3526, Corning). Cells were incubated at 37 °C and 5% CO_2_ overnight. The following day, 400 μL of the media were removed and a total of 200 μL growth medium was added, supplemented with ^3^H-PSMA-617 (926.6 GBq/mmol, Bayer AG) in the presence or absence of competing compounds in triplicate reactions. The cells were incubated under slight agitation for 10 min, followed by a 2-h incubation at 37 °C, 5% CO_2_. The supernatant was removed and the cells were washed twice with ice cold PBS. Cells were lysed in 500 μL of 0.3 M NaOH (PanReac AppliChem ITW Reagents) by vigorous shaking for 15 min and the cell lysates were transferred into scintillator tubes (S207 - SNAPTWIST® Scintillation Vials 6.5 ml #S207, 15 Simport). The wells were washed with 500 μL PBS and the wash solution was added to the corresponding samples in the scintillator tubes, along with 4 mL of Ultima Gold liquid scintillation cocktail (Sigma-Aldrich #L8286). After a 30-min incubation, the radioactivity was determined with a Tri-Carb Liquid Scintillation Analyzer (Perkin Elmer). Non-specific binding was assessed by measuring binding in the presence of unlabeled PSMA-617 (5000 nM, 500-fold excess) and was found to be less than 5%. The percentage of remaining binding of the ^3^H-PSMA-617 was calculated and the linker showing the strongest improvement in competing against ^3^H-PSMA-617 was chosen for further development.

**Evaluation of cytotoxicity**

The *in vitro* antiproliferative activity of monomeric, dimeric, and trimeric PSMA SMOL-TTCs was determined in LNCaP, C4-2, MDA-PCa-2b, VCaP, and 22Rv1 prostate cancer cells showing varying PSMA expression levels. PC-3 cells without PSMA expression were used as a negative control. Cytotoxicity was measured using the CellTiter-Glo® (Promega) proliferation assay after a 5-day incubation with a serial dilution of the compounds radiolabeled at 375 kBq/nmol. Cytotoxicity was similarly determined for ^227^Th-PSMA-617 and ^227^Th-*D*-Glu-PSMA-617 (a compound that due to a modification in the stereochemistry of the PSMA-binding motif binds to PSMA only very weakly). Dose response curves and the calculation of the IC_50_ values (50% inhibition of proliferation) were generated using BELLA-Dose Response Curve (DRC) spreadsheets. The DRC software is a Biobook Spreadsheet that was developed by Bayer AG and Bayer Business Services on the IDBS E-Workbook Suite platform (IDBS: ID Business Solutions Ltd., Guildford, UK). Results show the mean IC_50_ values calculated from 1 to 6 independent experiments.

**Binding, internalization, and induction of apoptosis *in vitro***

The binding and internalization of monomeric, dimeric, trimeric, and tetrameric PSMA SMOL-TTCs was determined on C4-2 and LNCaP prostate cancer cells. The results were compared to the binding and internalization of ^227^Th-PSMA-617 and ^227^Th-*D*-Glu-PSMA-617 in the same cells.

The experiments were performed by adding 7.5 x 10^6^ C4-2 cells or 7.5 x 10^5^ LNCaP cells per well on poly-L-lysine-coated plates (#3526, Corning Incorporated Costar). The plates were incubated at 37º C overnight. In the first C4-2 experiment, the PSMA SMOL conjugates were added into the wells at 0,3, 1, 3, 10, or 30 nM concentrations (radiolabeled at 375 kBq/nmol) and the binding and internalization were measured after a 2-h incubation at 37º C. In the second C4-2 experiment, the PSMA SMOL conjugates were used at a fixed concentration of 10 nM (radiolabeled at 375 kBq/nmol) and the measurements were performed after a 30-min, 2-h, 4-h, or 6-h incubation at 37º C. In the LNCaP experiment, the PSMA SMOL conjugates were used at a fixed concentration of 10 nM (radiolabeled at 375 kBq/nmol) and the measurements were performed after a 30-min or a 2-h incubation at 37º C.

For internalization assays, 24-well plates (#3526, Corning) were coated with 200 µL Poly-L-Lysine (0.1 mg/ml, #3438-100-01, Fa. Cultrex) per well at 37°C for 2 h. After two washes with growth medium, 7.5x10^5^ LNCaP cells were seeded in 500 µL growth medium (RPMI1640, 10% FCS Superior) in the coated wells. Cells were incubated at 37°C and 5%CO_2_ overnight. The next day, 400 µL of the medium was removed from each sample, and a total of 200 µL growth medium supplemented with PSMA SMOLs labeled with thorium-227 at 375 kBq/nmoL was added in triplicates. Cells were incubated with the indicated concentrations and incubation times at 37°C. Next, supernatants were discarded, and the cells were washed once with PBS, twice with glycine HCl buffer (50 mM; # 55097, Sigma Aldrich), and finally, once with PBS. The washing solutions were combined in counting vials (#TK75-018, Fa. Corning) and measured using a 1470 Wizard automatic gamma counter (Wallac). The resulting counts were attributed to membrane-bound radioactivity. Cells were then lysed with 500 µL of 0.3 N NaOH (#181691.1211, Fa. PanReac AppliChem ITW Reagents) and subsequently washed with 500 µL PBS. The internalized radioactivity fraction was determined by combining the NaOH lysate and the final wash solution and subjecting the sample to gamma counting as described above. Results were expressed as fmol of thorium-227 / 10^6^ cells in the membrane-bound and internalized fractions measured in triplicate. Representative samples were also analyzed using a HPGe detector with thorium-227 specific detection confirming that data from gamma counting were representative.

The cell-bound and internalized activity of thorium-227 was measured using a gamma counter (Wizard 2470, PerkinElmer). For standardization, aliquots of the radiolabeled PSMA SMOL conjugates were measured (cpm/pmol of radiolabeled compound). The amount of radiolabeled compound detected in the cell bound fraction as well as the internalized fraction was referred back to the number of cells in a given well and was expressed as fmol/10^6^ cells. Representative samples were also analyzed using a HPGe detector with thorium-227 specific detection confirming that data from gamma counting were representative.

The binding specificity of PSMA SMOL-TTCs (10 nM, radiolabeled at 375 kBq/nmol) was studied in PSMA-expressing C4-2 cells and PSMA-negative PC-3 cells using a glutamate carboxypeptidase II inhibitor 2-PMPA (30 μM) to compete for binding to PSMA after a 2-h incubation.

The ability of PSMA SMOL-TTCs to induce apoptosis was measured in VCaP prostate cancer cells using the Caspase-Glo® 3/7 assay (Promega) after a 3-day incubation. The effects were compared with ^227^Th-PSMA-617, and the fold induction was calculated against untreated control cells.

**Antitumor efficacy of PSMA SMOL conjugates *in vivo***

The *in vivo* antitumor efficacy of PSMA SMOL-TTCs was assessed in ST1273 and KUCaP-1 PDX models of human prostate cancer in mice. The KUCaP-1 study and the LNCaP biodistribution study were performed in-house and approved by Berlin authorities (Landesamt für Gesundheit und Soziales Berlin, LAGeSo); the ST1273 study and the biodistribution study in minipigs were conducted by Minerva Imaging (Copenhagen, Denmark), and the biodistribution study in cynomolgus monkeys by Invicro (Boston, MA, USA).

In the ST1273 model, female NMRI nude (RjOrI:NMRI-*Fox1^nu^/Foxn1^nu^*) mice (24-30 g, 6 weeks old, Janvier Labs) were implanted subcutaneously (s.c.) with ST1273 tumor fragments (5 x 5 x 5 mm). Two days prior to transplant, the mice were implanted with a testosterone rod (Testosterone MedRod, releasing 75 μg/day for 100 days, PreclinApps). Starting on day 27 after transplantation at an average tumor size of 330 mm^3^, mice (n=10/group) were randomized and treated with a single dose of vehicle (0.9% NaCl) or the monomer, dimer, trimer, or tetramer format of PSMA SMOL-TTC (1.5 MBq/kg, a single dose, i.v.). Along with body weights, tumors were measured twice weekly with calipers and their volume calculated with the formula: 0.52 x (length x width^2^).

In the KUCaP-1 model, male SCID (scid/scid) mice (22 g, 9 weeks old, Janvier Labs) were implanted s.c. with KUCaP-1 tumor fragments (3 x 3 x 3 mm). Starting on day 38 after transplantation at an average tumor size of 275 mm^3^, mice (n=10/group) were randomized and treated with vehicle (0.9% NaCl; Q4W, i.v.), or the monomer or the dimer format of PSMA SMOL-TTC (1 or 2 MBq/kg, Q4Wx2, i.v.). Along with body weights, tumors were measured three times a week with calipers and their volume calculated with the formula: 0.5 x (length x width^2^).

**Stability of PSMA SMOL conjugates**

Stability of PSMA SMOL-TTCs (monomer, dimer, trimer, and tetramer) was determined in citrate buffer (pH 4.5) or PBS at room temperature for 6 and 48 hours. Radiochemical purity was determined by instant thin-layer chromatography (iTLC). In addition, stability of PSMA SMOL-TTC (monomer) was determined at 37 °C in both human and mouse serum after incubation for 1, 2, 4, 20, 40, and 60 hours.

The stability of the [^89^Zr]-labeled PSMA SMOL conjugate (monomer) was evaluated in cynomolgus monkey serum. The monkey serum was thawed, aliquoted (10 mL), and the radiopeptide was added (approximately 30 MBq). This mixture was incubated at 37°C and sampled at each time point (0, 0.5, 4, 7, 24, 96, and 168 hours). At each time point, the pH of the serum solution as tested with pH paper. Post pH test, a serum sample was taken (1 mL) and the total activity of the sample was determined with the dose calibrator or gamma counter. Acetonitrile/water (50/50, 500 μL) was then added to the sample, mixed, and centrifuged (>15,000 RPM, 15 min). The supernatant was carefully transferred into a separate centrifuge tube, and activity was measured in both the supernatants and in the remaining pellets using a dose calibrator or a gamma counter, respectively. In addition, radiochemical purity (RCP) was determined from the supernatants via HPLC and iTLC. Experiments were performed in triplicate.

**Biodistribution of PSMA SMOL conjugates**

Biodistribution of PSMA SMOL conjugates was studied in ST1273 PDX tumor-bearing mice, healthy minipigs, healthy cynomolgus monkeys, and LNCaP tumor-bearing mice.

In the ST1273 biodistribution study, female NMRI nude (RjOrI:NMRI-*Fox1^nu^/Foxn1^nu^*) mice (24–30 g, 6 weeks old, Janvier Labs) were implanted s.c. with testosterone rods (Testosterone MedRod releasing 75 μg/day for 100 days, PreclinApps) and, two days later, with ST1273 tumor fragments (5 x 5 x 5 mm). On day 30 after transplantation, the mice (n=3/group/time point) were treated with a single dose of the monomer, dimer, trimer, or tetramer format of PSMA SMOL-TTC (1.5 MBq/kg, i.v.) and sacrificed at 5 min, 2 h, 24 h, 72 h, or 168 h after treatment. Blood, tumors, heart, spleen, femurs, liver, kidneys, and salivary glands were collected and analyzed for remaining thorium-227 activity using a HPGe detector.

The biodistribution of ^227^Th-PSMA-617 was studied in 6 healthy male Ellegaard Göttingen minipigs (12 months old, 25 kg, Ellegaard, Denmark). The animals were given a single dose of ^227^Th-PSMA-617 (200 kBq/kg, i.v.) and sacrificed 1 h, 24 h, and 72 h after dosing (n=2 animals/time point). Selected organs (salivary glands, lacrimal gland, liver, kidney, spleen, small intestine, colon, bone, bone marrow, prostate, lungs, and blood) were collected and analyzed for remaining thorium-227 activity using a HPGe well detector (Mirion). From two to three representative samples from each of the larger organs were measured and the mean value was calculated. The whole of the salivary glands was sliced into several pieces (n=20 for each gland) and all of them were measured.

Biodistribution of ^89^Zr-labeled PSMA SMOL conjugates in the monomer, dimer, and trimer formats was evaluated in healthy male cynomolgus monkeys (2–4 years old, 2.6–2.8 kg, Worldwide Primates, Inc). The monkeys (n=2/group) were given a single i.v. dose of the ^89^Zr-labeled PSMA-targeting monomer (1.17 ± 0.12 mCi), dimer (1.97 ± 0.09 mCi), or trimer (1.11 ± 0.01 mCi) peptide and evaluated by PET imaging (Siemens™ Focus 220 PET, Siemens) and CT imaging (CereTom OTOscan, NeuroLogica) 4 h, 24 h, 48 h, 96 h, and 168 h after dosing. The PET and CT images captured a whole-body field of view. The scan duration was 30 min for the 4-h and 24-h time points and 60 min for the 48, 96, and 168-h time points.

Biodistribution of ^89^Zr-labeled PSMA SMOL conjugates in the monomer, dimer, and trimer formats was evaluated in LNCaP tumor-bearing male SCID (scid/scid) mice (22 g, 8 weeks old, Janvier Labs) that had been implanted s.c. with testosterone pellets (12.5 mg, 4 mm). The mice (n=2/group) were given a single dose of the monomer, dimer, or trimer ^89^Zr-PSMA SMOL conjugate (3 MBq/mouse, i.v.) and evaluated by PET imaging (Inveon μPET/CT scanner, Siemens) 2 h, 24 h, 72 h, and 144/168 h after dosing. The scan duration was 10 min for the 2-h, 24-h, and 72-h timepoints and 20 min for the 144/168-h time point.

**General materials and methods for synthesis of compounds**

All reactants or reagents of which the preparation has not been described herein were purchased from generally accessible commercial sources and were used without further purification. For all other reactants or reagents for which the preparation likewise is not described herein and which were not commercially available or were obtained from sources which are not generally accessible, a reference is given to the published literature in which their preparation is described. All air and moisture-sensitive reactions were carried out in oven-dried (at 120 °C) glassware under an inert atmosphere of argon.

Reactions were monitored by thin layer chromatography (TLC) and ultra-performance liquid chromatography (UPLC) analysis.

Analytical TLC was carried out on aluminum-backed plates coated with Merck silica gel 60 F254, with visualization under UV light at 254 nm. Flash chromatography was carried out using a Biotage Isolera One system with a 200–400 nm variable detector.

# **Analytical LC-MS and HPLC conditions**

LC-MS-data given in the subsequent specific experimental descriptions refer (unless otherwise noted) to the following conditions:

#### Method 1:

Instrument: Waters Acquity UPLCMS SingleQuad; Column: Acquity UPLC BEH C18 1.7 µm, 50x2.1mm; eluent A: water + 0.1 vol-% formic acid (99%), eluent B: acetonitrile; gradient: 0-1.6 min 1-99% B, 1.6-2.0 min 99% B; flow 0.8 mL/min; temperature: 60 °C; DAD scan: 210-400 nm.

*Method 2:*

Instrument: SHIMADZU LCMS-2020 SingleQuad; Column: Chromolith@Flash RP-18E 25-2 MM; eluent A: water + 0.0375 vol-% trifluoroacetic acid, eluent B: acetonitrile + 0.01875 vol-% trifluoroacetic acid; gradient: 0-0.8 min, 5-95% B, 0.8-1.2 min 95% B; flow 1.5 mL/min; temperature: 50 °C; PDA: 220 nm & 254 nm.

*Method 3:*

Instrument: Agilent 1100\G1956A SingleQuad; Column: Kinetex@ 5 μm EVO C18 30*2.1 mm; eluent A: water + 0.0375 vol-% trifluoroacetic acid, eluent B: acetonitrile + 0.01875 vol-% trifluoroacetic acid; gradient: 0-0.8 min 5-95% B, 0.8-1.2 min 95% B; flow 1.5 mL/min; temperature: 50 °C; PDA: 220 nm & 254 nm.

*Method 4:*

Instrument: Waters Acquity/QTOF; Column: Phenomenex Kinetex 1.7 µm C18, 100 Å, 30 x 2.1 mm; eluent A: Water/0.1% TFA; eluent B: ACN/0.1% TFA; flow: 0.5 mL/min; temperature: ambient; detection: PDA.

# **Flash column chromatography conditions**

“Purification by (flash) column chromatography” as stated in the subsequent specific experimental descriptions refers to the use of a Biotage Isolera purification system. For technical specifications see “Biotage product catalogue” on www.biotage.com.

## **Synthesis of carboxy-HOPO chelator**

*Reaction sequence:*

###

Intermediates 1-3 shown below were prepared following the procedure published by Pailloux *et al*. [1].

### *Intermediate 1 for carboxy-HOPO chelator*

*Ethyl 3-hydroxy-6-methyl-2-oxo-1,2-dihydropyridine-4-carboxylate*

To a solution of sodium diethyloxylacetate (42.1 g, 200 mmol) in dry THF (500 mL) in a 1-liter 3-neck round bottom flask was added chloroacetone (16 mL, 200 mmol). After 10 min, NH_3_ gas was bubbled through the reaction followed by careful addition of AlCl_3_ (2.67 g, 20 mmol). The reaction was stirred under ambient temperature for 5 days. The resulting orange solid was filtered and taken up in 1 M HCl (500 mL) so that pH was below 3. The resulting suspension was stirred for 30 min and the precipitate filtered, washed with water, and dried to give 25.5 g (65%) of the target compound as a pale-yellow solid.

### *Intermediate 2 for carboxy-HOPO chelator*

*3-(benzyloxy)-6-methyl-2-oxo-1,2-dihydropyridine-4-carboxylate*

1,8-Diazabicyclo[5.4.0]undec-7-ene (30.0 g, 197 mmol) was added to a solution of ethyl 3-hydroxy-6-methyl-2-oxo-1,2-dihydropyridine-4-carboxylate (25.5 g, 129 mmol) in isopropanol (300 mL). The reaction mixture was refluxed at 83 °C under N_2_ before adding benzyl bromide (24 mL, 202 mmol) slowly. Refluxing was maintained for 4 h, and the solvent was evaporated. The resulting dark brown oil was dissolved in dichloromethane (100 mL), washed with aqueous 3 M HCl (2 x 100 mL) and water (3 x 100 mL). The organic layer was dried (Na_2_SO_4_), filtered, and concentrated. Diisopropyl ether (500 mL) was added to the oily residue and after spinning on the rotary evaporator the solid was filtered and dried to give 25 g (67%) of the target compound as a light grey solid. MS (ESIneg): m/z = 286.1 [M-H]^-^

### *Intermediate 3 for carboxy-HOPO chelator*

*Ethyl 3-(benzyloxy)-1-(2-(tert-butoxy)-2-oxoethyl)-6-methyl-2-oxo-1,2-dihydro-pyridine-4-carboxylate*

To a solution of tert-butylbromoacetate (25.0 g, 0.128 mol) in acetone (130 mL) was added sodium iodide (25.6 g, 0.17 mol). The reaction mixture was heated at reflux for 9 h, cooled to room temperature, filtered, and concentrated. The crude product was used without further purification.

To a 1 L round bottom flask was added 3-(benzyloxy)-6-methyl-2-oxo-1,2-dihydropyridine-4-carboxylate (20.9 g, 72.8 mmol) followed by potassium fluoride on alumina (40 g). These reagents were purged under nitrogen for 15 min after which dimethoxyethane (320 mL) was added. Tert-butyliodoacetate (35 g, 144 mmol) was added to the solution and the reaction was left to stir overnight, filtered and the filter cake washed thoroughly with THF. The filtrate was concentrated and the residue purified by flash chromatography (heptane – heptane/EtOAc 90:10 – 80:20 – 70:30 – 50:50) to afford 25.5 g (87%) of the target compound.

^1^H-NMR (400 MHz, DMSO-*d_6_*) δ 7.50 – 7.20 (m, 5H), 6.21 (s, 1H), 5.09 (s, 2H), 4.76 (s, 2H), 4.22 (q, J = 7.1, 2H), 3.32 (s, 2H), 2.50 (s, 4H), 2.26 (s, 3H), 1.44 (s, 9H), 1.21 (t, J = 7.1, 3H).

^13^C-NMR (101 MHz, DMSO-*d_6_*) δ 166.84, 164.69, 159.24, 142.90, 141.66, 136.94, 130.68, 128.30, 128.21, 128.16, 127.94, 102.59, 81.96, 72.87, 61.31, 46.63, 31.24, 28.35, 27.60, 27.28, 22.09, 19.41, 13.94, 13.89.

### *Intermediate 4 for carboxy-HOPO chelator*

*3-(Benzyloxy)-1-(2-(tert-butoxy)-2-oxoethyl)-6-methyl-2-oxo-1,2-dihydropyridine-4-carboxylic acid*

To a solution of ethyl 3-(benzyloxy)-1-(2-(tert-butoxy)-2-oxoethyl)-6-methyl-2-oxo-1,2-dihydropyridine-4-carboxylate (25.5 g, 63.5 mmol) in 1:1 THF/H2O (500 mL), aqueous LiOH (76 mL 1 M, 76.2 mmol) was added and the reaction mixture was stirred overnight at ambient temperature. Aqueous citric acid was added to neutral pH and the aqueous phase was extracted with EtOAc (x3). The combined organic extract was dried (Na_2_SO_4_), filtered, and concentrated. The residue was triturated with heptane/EtOAc 90:10 and the precipitated material filtered and dried to afford 21.9 g (92%) of the target compound as a colorless solid. MS (ESIpos): m/z = 374.1 [M+H]^+^. ^1^H-NMR (400 MHz, CDCl_3_) δ ppm 7.50 – 7.29 (m, 5H), 6.54 (d, J = 0.7, 1H), 5.53 (s, 2H), 4.72 (s, 2H), 2.26 (d, J = 0.6, 3H), 1.48 (s, 9H). ^13^C-NMR (101 MHz, CDCl_3_) δ 166.31, 163.79, 159.38, 145.20, 140.76, 134.63, 129.73, 129.65, 129.15, 126.82, 104.74, 83.51, 77.55, 77.23, 76.91, 75.75, 47.06, 28.20, 20.39.

### *Intermediate 5 for carboxy-HOPO chelator*

*Bis(2-azidoethyl)amine*

To a stirred solution of sodium azide (56.0 g, 0.86 mol) in water (500 mL), bis(2-chloroethyl)amine hydrochloride (76.8 g, 0.43 mol) was added. After stirring for 2 h at 90 °C, another portion of sodium azide (56.0 g, 0.86 g) was added, and the reaction mixture stirred for 48 h at 90 °C. After cooling to room temperature, the pH was adjusted to around 10 with aqueous NaOH (10 M). The aqueous solution was extracted with diethyl ether (x5). The combined organic extract was dried (Na_2_SO_4_), filtered and concentrated. Purification by chromatography (heptane/EtOAc gradient) afforded 45.5 g (68%) of the target compound as a yellow oil.

### *Intermediate 6 for carboxy-HOPO chelator*

*N1,N1,N3,N3-tetrakis(2-azidoethyl)propane-1,3-diamine*

To a solution of 1,3-dibromopropane (15 mL, 147 mmol), potassium carbonate (101 g, 730 mmol) and potassium iodide (48 g, 280 mmol) in acetonitrile (500 mL), bis(2-azidoethyl)amine dissolved in acetonitrile was added slowly, and the reaction mixture was heated at 80 °C for 48 h. The solid was removed by filtration and the filtrate concentrated *in vacuo*. The residue was purified by column chromatography using heptane/EtOAc (0-50% EtOAc) to afford 38 g (74%) of the target compound as a yellow oil. MS (ESIpos): m/z = 373.2 [M+Na]^+^.

### *Intermediate 7 for carboxy-HOPO chelator*

*N1,N1'-(propane-1,3-diyl)bis(N1-(2-aminoethyl)ethane-1,2-diamine)*

A solution of N1,N1,N3,N3-tetrakis(2-azidoethyl)propane-1,3-diamine (5.20 g) in EtOH (100 mL) was hydrogenated overnight at 5 bar in the presence of Pd (2 g). Filtration and concentration afforded the target compound as a yellow oil used without further purification. MS (ESIpos): m/z = 247.2 [M+H]^+^. ^13^C-NMR (101 MHz, D2O) δ 50.4, 49.7, 34.7, 19.00.

### *Intermediate 8 for carboxy-HOPO chelator*

*Tetra-tert-butyl 2,2',2'',2'''-((5,9-bis(2-formamidoethyl)-2,5,9,12-tetraazatridecanedioyl)-tetrakis-(3-(benzyloxy)-6-methyl-2-oxopyridine-4,1(2H)-diyl))tetraacetate*

To a solution of N1,N1'-(propane-1,3-diyl)bis(N1-(2-aminoethyl)ethane-1,2-diamine) (246 mg, 1 mmol) and 3-(benzyloxy)-1-(2-(tert-butoxy)-2-oxoethyl)-6-methyl-2-oxo-1,2-dihydropyridine-4-carboxylic acid (1.50 g, 4 mmol) in dimethylformamide (DMF, 15 mL) was added N,N-diisopropylethylamine (DIPEA, 2 mL, 12 mmol), followed by hexafluorophosphate azabenzotriazole tetramethyl uronium (HATU, 3.0 g, 8 mmol). The reaction mixture was stirred at room temperature overnight, poured into water, and extracted three times with EtOAc. The combined organic extract was washed with brine, dried (Na_2_SO_4_), filtered, and concentrated. Flash chromatography (DCM/EtOAc 50:50 – DCM/EtOAc/MeOH 47.5:47.5:5 – DCM/EtOAc/MeOH 45:45:10) afforded 0.45 g (27%) of the target compound as a yellow solid. Different batches were combined and purified by flash chromatography to afford 2.70 g of the target compound as a yellow solid used in the final step. LC-MS (Method 4, gradient: 10-70% B over 3 min): R_t_ = 3.01 min; MS (ESIpos): m/z = 1667.7 [M+H]^+^. ^1^H-NMR (400 MHz, CDCl_3_) δ ppm 8.13 (s, 3H), 7.39 – 7.25 (m, 18H), 6.37 (s, 4H), 5.27 (s, 8H), 4.68 (s, 8H), 3.39 – 3.03 (m, 9H), 2.72 (d, J = 44.6, 8H), 2.10 (d, J = 63.3, 15H), 1.50 – 1.34 (m, 40H)), and ^13^C-NMR (101 MHz, CDCl_3_) δ ppm 166.8, 144.1, 130.1, 129.2, 129.3, 128.5, 83.2, 74.9, 55.9, 46.9, 38.8, 28.2, 20.2).

*Final carboxy-HOPO chelator*

*2,2',2'',2'''-(propane-1,3-diylbis{nitrilobis[(ethane-2,1-diyl)carbamoyl(3-hydroxy-6-methyl-2-oxopyridine-4,1(2H)-diyl)]})tetraacetic acid*

Tetra-tert-butyl 2,2',2'',2'''-((5,9-bis(2-formamidoethyl)-2,5,9,12-tetraazatridecanedioyl)-tetrakis-(3-(benzyloxy)-6-methyl-2-oxopyridine-4,1(2H)-diyl))tetraacetate (2.70 g) was treated with concentrated hydrochloric acid (100 mL) at room temperature for 3 h and concentrated to dryness by evaporation *in vacuo*. The residue was purified by reverse phase flash chromatography (0-50% ACN in water) to give 2.2 g of the target compound. This material was again purified using preparative HPLC (column: Phenomenex Luna 5 µm C18(2) 100Å, 250 x 50 mm; mobile phase: water/0.1% TFA; ACN; gradient: 0-30% B over 40 min; flow: 50 mL/min; detection: UV 280/335 nm) to afford 400 mg of the target compound. LC-MS (Method 4, gradient: 5-30% B over 3 min): R_t_ = 1.68 min; MS (ESIpos): m/z = 1083.5 [M+H]^+^. ^1^H-NMR (400 MHz, DMSO-*d_6_*) δ ppm 11.03 (4H), 8.62 (s, 4H), 6.39 (s, 4H), 4.74 (s, 8H), 4.16 – 2.85 (m, 26H), 2.19 (s, 12H)), and ^13^C-NMR (101 MHz, DMSO-*d_6_*) δ ppm 169.3, 165.5, 158.9, 144.3, 134.3, 117.5, 102.9, 51.7, 46.1, 34.6, 19.1).

**Synthesis of linkers**

*Reaction sequence:*

**Synthesis of DOTA conjugates**

*Reaction sequence:*

**Synthesis of Compound B**

### *Intermediate 1 for Compound B*

*Tri-tert-butyl (5S,12S,16S)-1-(9H-fluoren-9-yl)-3,6,14-trioxo-5-[(quinoline-2-yl)methyl]-2-oxa-4,7,13,15-tetraazaoctadecane-12,16,18-tricarboxylate*

Di-tert-butyl N-{[(2S)-6-amino-1-tert-butoxy-1-oxohexan-2-yl]carbamoyl}-L-glutamate (567 mg, 1.16 mmol) and N-{[(9H-fluoren-9-yl)methoxy]carbonyl}-3-quinolin-2-yl-L-alanine (510 mg, 1.16 mmol) were solubilized in DMF (8.9 ml), 4-methylmorpholine (320 µL, 2.9 mmol, CAS-RN: 109-02-4) and HATU (663 mg, 1.74 mmol) were added, and the mixture was stirred under argon atmosphere at room temperature for 3 h. The mixture was evaporated, diluted with water, and extracted with DCM/isopropanol (4:1). The organic layer was dried, evaporated, and purified by preparative HPLC (C18, acetonitrile/water with 0.1% formic acid) to give 433 mg (95% purity, 41% yield) of the target compound. LC-MS (Method 1): Rt = 1.59 min; MS (ESIpos): m/z = 909 [M+H]^+^. ^1^H-NMR (400 MHz, DMSO-*d*_6_) δ ppm 8.25 (d, *J*=8.62 Hz, 1 H) 8.00 (br t, *J*=5.70 Hz, 1 H) 7.93 (dd, *J*=7.35, 5.58 Hz, 2 H) 7.85 (d, *J*=7.35 Hz, 2 H) 7.65 – 7.76 (m, 2 H) 7.52 – 7.63 (m, 3 H) 7.47 (d, *J*=8.36 Hz, 1 H) 7.38 (td, *J*=7.16, 3.68 Hz, 2 H) 7.16 – 7.29 (m, 2 H) 6.27 (dd, *J*=14.07, 8.24 Hz, 2 H) 4.53 (td, *J*=8.81, 5.20 Hz, 1 H) 4.08 – 4.22 (m, 3 H) 4.03 (td, *J*=8.55, 5.45 Hz, 1 H) 3.86 – 3.96 (m, 1 H) 3.27 (d, *J*=5.32 Hz, 1 H) 3.14 – 3.20 (m, 1 H) 2.91 – 3.12 (m, 2 H) 2.13 – 2.28 (m, 2 H) 1.80 – 1.92 (m, 1 H) 1.60 – 1.73 (m, 1 H) 1.51 – 1.57 (m, 1 H) 1.38 (s, 30 H) 1.22 (br d, *J*=5.07 Hz, 2 H).

### *Intermediate 2 for Compound B*

*Di-tert-butyl (2S)-2-({[(2S)-6-{[(2S)-2-amino-3-(quinoline-2-yl)propanoyl]amino}-1-tert-butoxy-1-oxohexan-2-yl]carbamoyl}amino)pentanedioate*

Tri-tert-butyl (5S,12S,16S)-1-(9H-fluoren-9-yl)-3,6,14-trioxo-5-[(quinoline-2-yl)methyl]-2-oxa-4,7,13,15-tetraazaoctadecane-12,16,18-tricarboxylate (366 mg, 403 µmol) was solubilized in DMF (3.1 mL), piperidine (800 µL, 8.1 mmol) was added, and the mixture was stirred under argon atmosphere at room temperature for 2 h. The mixture was evaporated and purified by preparative HPLC (C18, acetonitrile/water with 0.1% formic acid) to give 83.0 mg (100% purity, 51% yield) of the target compound. LC-MS (Method 1): Rt = 1.16 min; MS (ESIpos): m/z = 687 [M+H]^+^. ^1^H-NMR (400 MHz, DMSO-*d*_6_) δ ppm 8.26 (d, *J*=8.11 Hz, 1 H) 8.21 (s, 1 H) 7.90 – 8.01 (m, 3 H) 7.72 (ddd, *J*=8.49, 6.97, 1.27 Hz, 1 H) 7.51 – 7.58 (m, 1 H) 7.42 (d, *J*=8.36 Hz, 1 H) 6.28 (dd, *J*=18.50, 8.36 Hz, 2 H) 4.03 (td, *J*=8.49, 5.07 Hz, 1 H) 3.93 (td, *J*=8.05, 5.45 Hz, 1 H) 3.73 (dd, *J*=8.62, 4.82 Hz, 1 H) 3.26 (br dd, *J*=13.81, 4.69 Hz, 2 H) 2.98 – 3.13 (m, 2 H) 2.13 – 2.30 (m, 2 H) 1.80 – 1.91 (m, 1 H) 1.42 – 1.75 (m, 4 H) 1.32 – 1.42 (m, 29 H) 1.18 – 1.28 (m, 2 H).

### *Intermediate 3 for Compound B*

*Tri-tert-butyl (3S,10S,14S)-1-{(1r,4S)-4-[({[(9H-fluoren-9-yl)methoxy]carbonyl}amino) methyl]cyclohexyl}-1,4,12-trioxo-3-[(quinoline-2-yl)methyl]-2,5,11,13-tetraazahexadecane-10,14,16-tricarboxylate*

Di-tert-butyl (2S)-2-({[(2S)-6-{[(2S)-2-amino-3-(quinoline-2-yl)propanoyl]amino}-1-tert-butoxy-1-oxohexan-2-yl]carbamoyl}amino)pentanedioate (67.0 mg, 97.7 µmol) and (1r,4r)-4-[({[(9H-fluoren-9-yl)methoxy]carbonyl}amino)methyl]cyclohexane-1-carboxylic acid (37.1 mg, 97.7 µmol) were solubilized in DMF (1.1 mL), 4-methylmorpholine (32 µL, 290 µmol, CAS-RN: 109-02-4) and HATU (39.0 mg, 103 µmol) were added, and the mixture was stirred under argon atmosphere at room temperature overnight. The mixture was evaporated and purified by preparative HPLC (C18, acetonitrile/water with 0.1% formic acid) to give 34.0 mg (95% purity, 32% yield) of the target compound. ^1^H-NMR (400 MHz, DMSO-*d*_6_) δ ppm 8.24 (d, *J*=8.62 Hz, 1 H) 8.01 (d, *J*=8.36 Hz, 1 H) 7.82 – 7.96 (m, 5 H) 7.68 (d, *J*=7.60 Hz, 3 H) 7.50 – 7.57 (m, 1 H) 7.36 – 7.47 (m, 3 H) 7.20 – 7.35 (m, 3 H) 6.27 (dd, *J*=19.26, 8.36 Hz, 2 H) 4.74 (td, *J*=8.74, 5.58 Hz, 1 H) 4.29 (d, *J*=6.84 Hz, 2 H) 4.14 – 4.23 (m, 1 H) 4.04 (td, *J*=8.55, 5.20 Hz, 1 H) 3.85 – 3.97 (m, 1 H) 3.28 (dd, *J*=13.69, 5.32 Hz, 1 H) 2.91 – 3.19 (m, 3 H) 2.78 (br t, *J*=6.34 Hz, 2 H) 2.12 – 2.30 (m, 2 H) 1.99 – 2.09 (m, 1 H) 1.80 – 1.93 (m, 1 H) 1.42 – 1.71 (m, 7 H) 1.38 (d, *J*=1.77 Hz, 27 H) 1.13 – 1.35 (m, 7 H) 0.94 – 1.08 (m, 1 H) 0.75 – 0.86 (m, 1 H).

### *Intermediate 4 for Compound B*

*Tri-tert-butyl (3S,10S,14S)-1-[(1r,4S)-4-(aminomethyl)cyclohexyl]-1,4,12-trioxo-3-[quinoline-2-yl)methyl]-2,5,11,13-tetraazahexadecane-10,14,16-tricarboxylate*

Tri-tert-butyl (3S,10S,14S)-1-{(1r,4S)-4-[({[(9H-fluoren-9-yl)methoxy]carbonyl}amino) methyl]cyclohexyl}-1,4,12-trioxo-3-[(quinoline-2-yl)methyl]-2,5,11,13-tetraazahexadecane-10,14,16-tricarboxylate (63.0 mg, 60.2 µmol) was solubilized in DMF (1.9 mL), piperidine (120 µL, 1.2 mmol) was added, and the mixture was stirred under argon atmosphere at room temperature for 1.5 h. The mixture was evaporated and purified by preparative HPLC (C18, acetonitrile/water with 0.1% formic acid) to give 28.0 mg (100% purity, 56% yield) of the target compound. LC-MS (Method 1): Rt = 1.14 min; MS (ESIpos): m/z = 826 [M+H]^+^. ^1^H-NMR (400 MHz, DMSO-*d*_6_) δ ppm 8.42 (s, 1 H) 8.24 (d, *J*=8.62 Hz, 1 H) 8.05 (d, *J*=8.62 Hz, 1 H) 7.84 – 7.98 (m, 3 H) 7.72 (ddd, *J*=8.43, 7.03, 1.52 Hz, 1 H) 7.54 (td, *J*=7.54, 1.14 Hz, 1 H) 7.42 (d, *J*=8.62 Hz, 1 H) 6.29 (dd, *J*=19.77, 8.36 Hz, 2 H) 4.74 (td, *J*=8.87, 5.32 Hz, 1 H) 4.03 (td, *J*=8.49, 5.32 Hz, 1 H) 3.86 – 3.98 (m, 1 H) 3.25 – 3.31 (m, 4 H) 2.91 – 3.17 (m, 3 H) 2.13 – 2.30 (m, 2 H) 1.98 – 2.11 (m, 1 H) 1.80 – 1.93 (m, 1 H) 1.60 – 1.77 (m, 4 H) 1.41 – 1.58 (m, 3 H) 1.38 (d, *J*=1.52 Hz, 27 H) 1.32 (br s, 2 H) 1.15 – 1.27 (m, 4 H) 0.96 – 1.10 (m, 1 H) 0.75 – 0.91 (m, 2 H).

### *Intermediate 5 for Compound B*

*Tri-tert-butyl (3S,10S,14S)-1,4,12-trioxo-3-[(quinoline-2-yl)methyl]-1-[4-({2-[4,7,10-tris(2-tert-butoxy-2-oxoethyl)-1,4,7,10-tetraazacyclododecan-1-yl]acetamido}methyl)cyclohexyl]-2,5,11,13-tetraazahexadecane-10,14,16-tricarboxylate*

(1r,4r)-4-({2-[4,7,10-tris(2-tert-butoxy-2-oxoethyl)-1,4,7,10-tetraazacyclododecan-1-yl]acetamido}methyl)cyclohexane-1-carboxylic acid (71.6 mg, 101 µmol), [(1H-benzotriazol-1-yl)oxy](dimethylamino)-N,N-dimethylmethaniminium hexafluoridophosphate(1-) (45.9 mg, 121 µmol) and 4-methyl morpholine (33 µl, 300 µmol) were stirred in DMF (1.0 mL) at room temperature for 20 min. di-tert-butyl (2S)-2-({[(2S)-6-{[(2S)-2-amino-3-(quinoline-2-yl)propanoyl]amino}-1-tert-butoxy-1-oxohexan-2-yl]carbamoyl}amino)pentanedioate (69.0 mg, 101 µmol) was added and the mixture was stirred at room temperature for 14 h. The mixture was diluted with dichloromethane and isopropanol and washed with water. The organic phase was filtered through a phase separator and concentrated under reduced pressure. The residue was purified by preparative HPLC (C18, acetonitrile/water with 0.1% formic acid) to give 138 mg (99% yield) of the target compound which was directly used in the next step.

*Compound B*

*N^6^-{3-(quinolin-2-yl)-N-[(1r,4S)-4-({2-[4,7,10-tris(carboxymethyl)-1,4,7,10-tetraazacyclododecan-1-yl]acetamido}methyl)cyclohexane-1-carbonyl]-L-alanyl}-N^2^-{[(1S)-1,3-dicarboxypropyl]carbamoyl}-L-lysine*

Tri-tert-butyl (3S,10S,14S)-1,4,12-trioxo-3-[(quinolin-2-yl)methyl]-1-[(1r,4S)-4-({2-[4,7,10-tris(2-tert-butoxy-2-oxoethyl)-1,4,7,10-tetraazacyclododecan-1-yl]acetamido}methyl)cyclohexyl]-2,5,11,13-tetraazahexadecane-10,14,16-tricarboxylate (100 mg, 72.5 µmol) was solubilized in DCM (2.8 mL), TFA (2.8 mL, 36 mmol) was added, and the mixture was stirred under argon atmosphere at room temperature for 4.5 h. The mixture was evaporated and purified by preparative HPLC (C18, acetonitrile/water with 0.1% formic acid) to give 20.0 mg (95% purity, 25% yield) of the target compound. LC-MS (Method 1): Rt = 0.56 min; MS (ESIneg): m/z = 1042 [M-H]^-^. ^1^H-NMR (400 MHz, DMSO-*d*_6_) δ ppm 8.24 (d, *J*=8.36 Hz, 1 H) 8.13 (s, 2 H) 8.04 - 8.12 (m, 2 H) 7.83 - 7.99 (m, 3 H) 7.72 (td, *J*=7.73, 1.52 Hz, 1 H) 7.50 - 7.58 (m, 1 H) 7.42 (d, *J*=8.62 Hz, 1 H) 6.30 (br dd, *J*=14.07, 8.24 Hz, 2 H) 4.73 (td, *J*=8.62, 5.32 Hz, 1 H) 4.06 - 4.14 (m, 1 H) 3.95 - 4.05 (m, 1 H) 3.45 - 3.54 (m, 4 H) 3.28 (br m, 6 H) 3.08 - 3.16 (m, 2 H) 2.87 - 3.07 (m, 12 H) 2.73 (s, 3 H) 2.15 - 2.30 (m, 3 H) 2.05 (br s, 1 H) 1.91 (br d, *J*=6.84 Hz, 1 H) 1.54 - 1.78 (m, 5 H) 1.14 - 1.53 (m, 10 H) 0.99 - 1.12 (m, 1 H) 0.72 - 0.88 (m, 2 H)

**Synthesis of Compound C**

### *Intermediate 1 for Compound C*

*Isoquinoline-3-carbaldehyde*

To a mixture of methyl isoquinoline-3-carboxylate (7.80 g, 41.7 mmol) in toluene (160 mL) was added diisobutylaluminum hydride (1 M in toluene, 63 mL, 63 mmol) dropwise at -60 °C under nitrogen atmosphere. The reaction mixture was stirred at ‑60 °C for 2 h. The mixture was quenched with saturated ammonium chloride solution and saturated potassium tartrate. After stirring at room temperature for 16 h, the mixture was extracted with ethyl acetate. The combined organic layers were washed with brine, dried over anhydrous sodium sulfate, filtered, and concentrated under reduced pressure to give a residue. The residue was purified by column chromatography (200-300 mesh, petroleum ether: ethyl acetate = 50:1 to 1:1) to give isoquinoline-3-carbaldehyde (2.60 g, 90% purity, 36% yield) as a yellow solid. ^1^H-NMR (400 MHz, CDCl_3_): δ ppm = 10.30 (s, 1H), 9.42 (s, 1H), 9.43 (s, 1H), 8.11 (dd, 1H), 8.06 (dd, 1H), 7.86-7.82 (m, 2H).

### *Intermediate 2 for Compound C*

*Methyl (2Z)-2-{[(benzyloxy)carbonyl]amino}-3-(isoquinolin-3-yl)prop-2-enoate*

To a solution of methyl {[(benzyloxy)carbonyl]amino}(dimethoxyphosphoryl)acetate (10.9 g, 33.0 mmol) in dichloromethane (100 mL), 1,8-diazabicyclo(5.4.0)undec-7-ene (4.9 ml, 33 mmol) was added dropwise at 0-5 °C. After stirring for 30 min, a solution of isoquinoline-3-carbaldehyde (4.80 g, 90% purity, 27.5 mmol) in dichloromethane (100 mL) was added dropwise at 0-5 °C. After stirring at room temperature for 2 h, the reaction mixture (combined with another batch) was diluted with water and extracted with ethyl acetate. The organic phase was washed with brine, dried over anhydrous sodium sulfate, filtered, and concentrated *in vacuo*. The residue was purified by column chromatography on silica gel (200-300 mesh, petroleum ether: ethyl acetate = 20:1 to 5:1) to give methyl (2Z)-2-{[(benzyloxy)carbonyl]amino}-3-(isoquinolin-3-yl)acrylate (7.20 g, 99% purity, 72% yield) as a yellow solid. ^1^H-NMR (400 MHz, CDCl_3_): δ ppm = 11.05 (s, 1H), 9.13 (s, 1H), 7.89 (d, 1H), 7.73 (d, 1H), 7.64 (td, 1H), 7.56-7.52 (m, 2H), 7.34-7.28 (m, 5H), 6.45 (s, 1H), 5.13 (s, 2H), 3.78 (s, 3H).

### *Intermediate 3 for Compound C*

*(rac)-methyl N-[(benzyloxy)carbonyl]-3-isoquinolin-3-yl-alaninate*

To a solution of (2Z)-2-{[(benzyloxy)carbonyl]amino}-3-(isoquinolin-3-yl)prop-2-enoate (4.70 g, 99% purity, 12.8 mmol) and nickel(II) chloride hexahydrate (305 mg, 1.28 mmol) in methanol (120 mL)/tetrahydrofuran (40 mL) was added sodium borohydride (1.46 g, 38.5 mmol) in three portions at 0 °C. After stirring at room temperature for 16 h, the reaction mixture (combined with another batch) was diluted with water and extracted with ethyl acetate. The organic phase was washed with brine, dried over sodium sulfate, filtered, and the filtrate was concentrated under reduce pressure to afford methyl N-[(benzyloxy)carbonyl]-3-isoquinolin-3-ylalaninate (4.45 g, 90% purity, 86% yield) as a yellow oil. LC-MS (Method 2): R_t_ = 0.782 min; MS (ESIpos): m/z = 365.2 [M+H]^+^.

### *Intermediate 4 for Compound C*

*(rac)-N-[(benzyloxy)carbonyl]-3-isoquinolin-3-yl-alanine*

To a solution of methyl N-[(benzyloxy)carbonyl]-3-isoquinolin-3-yl-alaninate (3.85 g, 90% purity, 9.51 mmol) in THF (100 mL) and water (100 mL) was added lithium hydroxide (1.20 g, 28.5 mmol). The reaction mixture was stirred at room temperature for 2 h. The reaction mixture was acidified to pH = 3 by HCl (6 M), diluted with water, and extracted with EtOAc. The organic phase was washed with brine, dried, and concentrated *in vacuo* to give 2.20 g (94% purity, 62% yield) of the target compound.

### *Intermediate 5 for Compound C*

*N-[(benzyloxy)carbonyl]-3-isoquinolin-3-yl-L-alanine*

(Rac)N-[(benzyloxy)carbonyl]-3-isoquinolin-3-yl-L-alanine (2.20 g, 94% purity, 5.90 mmol) was separated by preparative-SFC (instrument: Waters 80Q SFC; column: Chiralcel OD column, 250×25 mm×10 μm particle size; mobile phase: Phase A for Supercritical carbon dioxide, Phase B for ethanol (0.1% ammonia water); isocratic elution: 40% Phase B (60% Phase A); flow: 70 g/min; cycle time: 4.6 min; back pressure: 100 bar to keep the carbon dioxide in supercritical flow; temperature: room temperature; detector: UV 220 nm) to give (Ent)N-[(benzyloxy)carbonyl]-3-isoquinolin-3-ylalanine (stereoisomer 1, first eluting, SFC retention time: 1.595, 865 mg, 97% purity) as an off-white solid and (Ent)N-[(benzyloxy)carbonyl]-3-isoquinolin-3-ylalanine (stereoisomer 2, second eluting, SFC retention time: 1.969, specific rotation (calc.): -9.458°, concentration: 0.1882 g/100 mL in methanol, 25 °C, 698 mg, 97% purity) as a white solid. SFC (Method: Cellucoat-Isopropanol (diethylamine)-20-3 ml-35T); instrument: SHIMADZU-2020; column: Cellucoat 50×4.6mm I.D., 3 μm; mobile phase: 20% iso-propanol (0.05% diethylamine) in carbon dioxide 5-40%; flow rate: 3 mL/min; temperature: 35 °C; detector: 220 nm. LC-MS (Method 2): R_t_ = 0.637 min; MS (ESIpos): m/z = 351.0 [M+H]^+^. ^1^H-NMR (400 MHz, DMSO-*d_6_*): δ ppm = 9.27 (s, 1H), 8.10 (d, 1H), 7.88 (d, 1H), 7.76 (td, 1H), 7.65-7.62 (m, 3H), 7.26-7.21 (m, 5H), 4.98-4.91 (m, 2H), 4.60-4.56 (m, 1H), 3.37-3.36 (m, 1H), 3.19-3.13 (m, 1H). LC-MS (Method 2): R_t_ = 0.637 min; MS (ESIpos): m/z = 351.1 [M+H]^+^. ^1^H-NMR (400 MHz, DMSO-*d_6_*): δ ppm = 9.27 (s, 1H), 8.10 (d, 1H), 7.88 (d, 1H), 7.76 (td, 1H), 7.67-7.63 (m, 3H), 7.26-7.21 (m, 5H), 4.98-4.91 (m, 2H), 4.60-4.56 (m, 1H), 3.37-3.36 (m, 1H), 3.19-3.13 (m, 1H).

### *Intermediate* *6 for Compound C*

*Tri-tert-butyl (5S,12S,16S)-5-[(isoquinolin-3-yl)methyl]-3,6,14-trioxo-1-phenyl-2-oxa-4,7,13,15-tetraazaoctadecane-12,16,18-tricarboxylate*

Di-tert-butyl N-{[(2S)-6-amino-1-tert-butoxy-1-oxohexan-2-yl]carbamoyl}-L-glutamate (615 mg, 1.26 mmol) and N-[(benzyloxy)carbonyl]-3-isoquinolin-3-yl-L-alanine (442 mg, 1.26 mmol) were solubilized in DMF (9.7 mL), 4-methylmorpholine (350 µL, 3.2 mmol, CAS-RN: 109-02-4) and HATU (671 mg, 1.77 mmol) were added, and the mixture was stirred under argon atmosphere at room temperature overnight. The mixture was diluted with DCM/propan-2-ol and washed with water and brine. The organic layer was dried, evaporated, and purified by flash chromatography (SiO_2_, DCM/Ethanol gradient 0-5%) to give 400 mg (100% purity, 39% yield) of the target compound. LC-MS (Method 1): Rt = 1.45 min; MS (ESIpos): m/z = 821 [M+H]^+^.

### *Intermediate* *7 for Compound C*

*Di-tert-butyl (2S)-2-({[(2S)-6-{[(2S)-2-amino-3-(isoquinolin-3-yl)propanoyl]amino}-1-tert-butoxy-1-oxohexan-2-yl]carbamoyl}amino)pentanedioate*

Tri-tert-butyl (5S,12S,16S)-5-[(isoquinolin-3-yl)methyl]-3,6,14-trioxo-1-phenyl-2-oxa-4,7,13,15-tetraazaoctadecane-12,16,18-tricarboxylate (400 mg, 488 µmol) was solubilized in MeOH (4.0 mL), palladium on carbon (51.9 mg, 10% purity, 48.8 µmol) was added, and the mixture was purged with hydrogen. The mixture was stirred under hydrogen atmosphere at room temperature for 6 h. The mixture was filtered over Celite, washed with MeOH, and evaporated to give 300 mg (94% purity, 84% yield) of the target compound. LC-MS (Method 1): Rt = 1.12 min; MS (ESIpos): m/z = 687 [M+H]^+^. ^1^H-NMR (400 MHz, DMSO-*d*_6_) δ ppm 9.25 (s, 1 H) 8.08 (d, *J*=8.36 Hz, 1 H) 7.87 (br d, *J*=8.62 Hz, 2 H) 7.73 (ddd, *J*=8.17, 6.91, 1.14 Hz, 1 H) 7.55 - 7.64 (m, 1 H) 6.17 - 6.36 (m, 2 H) 4.03 (td, *J*=8.49, 5.32 Hz, 1 H) 3.87 - 3.96 (m, 1 H) 3.63 (dd, *J*=8.74, 4.69 Hz, 1 H) 3.13 - 3.25 (m, 1 H) 3.02 (dt, *J*=13.56, 6.65 Hz, 2 H) 2.86 (dd, *J*=13.43, 8.87 Hz, 1 H) 2.12 - 2.29 (m, 2 H) 1.79 - 1.96 (m, 2 H) 1.68 (m, 2 H) 1.49 - 1.59 (m, 1 H) 1.09 - 1.48 (m, 32 H).

### *Intermediate* *8 for Compound C*

*Tri-tert-butyl (3S,10S,14S)-1-[(1r,4S)-4-({[(benzyloxy)carbonyl]amino}methyl)cyclohexyl]-3-[(isoquinolin-3-yl)methyl]-1,4,12-trioxo-2,5,11,13-tetraazahexadecane-10,14,16-tricarboxylate*

Di-tert-butyl (2S)-2-({[(2S)-6-{[(2S)-2-amino-3-(isoquinolin-3-yl)propanoyl]amino}-1-tert-butoxy-1-oxohexan-2-yl]carbamoyl}amino)pentanedioate (300 mg, 437 µmol) and (1r,4r)-4-({[(benzyloxy)carbonyl]amino}methyl)cyclohexane-1-carboxylic acid (127 mg, 437 µmol) were solubilized in DMF (5 mL), 4-methylmorpholine (140 µL, 1.3 mmol, CAS-RN: 109-02-4) and HATU (200 mg, 525 µmol) were added, and the mixture was stirred under argon atmosphere at room temperature overnight. The mixture was evaporated and purified by preparative HPLC (C18, acetonitrile/water with 0.1% formic acid) to give 165 mg (95% purity, 37% yield) of the target compound. LC-MS (Method 1): R_t_ = 1.46 min; MS (ESIpos): m/z = 960 [M+H]^+^. ^1^H-NMR (400 MHz, DMSO-*d*_6_) δ ppm 9.23 (s, 1 H) 8.07 (d, *J*=7.86 Hz, 1 H) 7.94 (d, *J*=7.86 Hz, 1 H) 7.81 - 7.88 (m, 2 H) 7.69 - 7.76 (m, 1 H) 7.54 - 7.65 (m, 2 H) 7.27 - 7.39 (m, 5 H) 7.18 - 7.26 (m, 1 H) 6.19 - 6.31 (m, 2 H) 4.98 (s, 2 H) 4.62 - 4.73 (m, 1 H) 4.04 (td, *J*=8.49, 5.07 Hz, 1 H) 3.84 - 3.95 (m, 1 H) 3.23 (dd, *J*=13.56, 5.45 Hz, 1 H) 2.87 - 3.09 (m, 2 H) 2.80 (t, *J*=6.34 Hz, 2 H) 2.13 - 2.28 (m, 2 H) 1.96 - 2.09 (m, 1 H) 1.80 - 1.91 (m, 1 H) 1.42 - 1.73 (m, 5 H) 1.38 (d, *J*=0.76 Hz, 28 H) 1.23 (br s, 8 H) 0.92 - 1.07 (m, 1 H) 0.71 - 0.89 (m, 2 H).

### *Intermediate 9 for Compound C*

*Tri-tert-butyl (3S,10S,14S)-1-[(1r,4S)-4-(aminomethyl)cyclohexyl]-3-[(isoquinolin-3-yl)methyl]-1,4,12-trioxo-2,5,11,13-tetraazahexadecane-10,14,16-tricarboxylate*

Tri-tert-butyl (3S,10S,14S)-1-[(1r,4S)-4-({[(benzyloxy)carbonyl]amino}methyl)cyclo-hexyl]-3-[(isoquinolin-3-yl)methyl]-1,4,12-trioxo-2,5,11,13-tetraazahexadecane-10,14,16-tricarboxylate (170 mg, 177 µmol) was solubilized in MeOH (1.4 mL). Palladium on carbon (18.9 mg, 10% purity, 17.7 µmol) was added, and the mixture was purged with hydrogen. The mixture was stirred under hydrogen atmosphere at room temperature for 8 h. The mixture was filtered over Celite, washed with MeOH, and evaporated to give 120 mg (95% purity, 78% yield) of the target compound. LC-MS (Method 1): Rt = 1.09 min; MS (ESIpos): m/z = 826 [M+H]^+^. ^1^H-NMR (400 MHz, DMSO-*d*_6_) δ ppm 9.23 (s, 1 H) 8.04 - 8.11 (m, 1 H) 7.90 - 8.00 (m, 1 H) 7.77 - 7.88 (m, 2 H) 7.68 - 7.75 (m, 1 H) 7.53 - 7.66 (m, 2 H) 6.20 - 6.34 (m, 2 H) 4.62 - 4.75 (m, 1 H) 3.97 - 4.07 (m, 1 H) 3.86 - 3.95 (m, 1 H) 3.14 - 3.27 (m, 2 H) 2.86 - 3.14 (m, 2 H) 2.20 (s, 2 H) 1.96 - 2.12 (m, 1 H) 1.79 - 1.93 (m, 1 H) 1.56 - 1.75 (m, 4 H) 1.38 (s, 29 H) 1.23 (br s, 8 H) 0.96 - 1.07 (m, 1 H) 0.64 - 0.89 (m, 2 H).

### *Intermediate* *10 for Compound C*

*Tri-tert-butyl (3S,10S,14S)-3-[(isoquinolin-3-yl)methyl]-1,4,12-trioxo-1-[(1r,4S)-4-({2-[4,7,10-tris(2-tert-butoxy-2-oxoethyl)-1,4,7,10-tetraazacyclododecan-1-yl]acetamido}methyl)cyclohexyl]-2,5,11,13-tetraazahexadecane-10,14,16-tricarboxylate*

[4,7,10-tris(2-tert-butoxy-2-oxoethyl)-1,4,7,10-tetraazacyclododecan-1-yl]acetic acid (69.4 mg, 121 µmol; CAS-RN:[137076-54-1]), [(1H-benzotriazol-1-yl)oxy](dimethylamino)-N,N-dimethylmethaniminium hexafluoridophosphate(1-) (45.4 mg, 120 µmol; CAS-RN:94790-37-1) and N,N-diisopropylethylamine (17 µL, 110 µmol) were stirred in DMF (1.2 mL) at room temperature for 10 min. Tri-tert-butyl (3S,10S,14S)-1-[(1r,4S)-4-(aminomethyl)cyclohexyl]-3-[(isoquinolin-3-yl)methyl]-1,4,12-trioxo-2,5,11,13-tetraazahexadecane-10,14,16-tricarboxylate (25.0 mg, 30.3 µmol) was added and the mixture was stirred at room temperature overnight. The mixture was evaporated and purified by preparative HPLC (C18, acetonitrile/water with 0.1% formic acid) to give 25.0 mg (97% purity, 58% yield) of the target compound. LC-MS (Method 1): Rt = 1.38 min; MS (ESIpos): m/z = 1380 [M+H]^+^.

### *Compound C*

*N^6^-{3-(isoquinolin-3-yl)-N-[(1r,4S)-4-({2-[4,7,10-tris(carboxymethyl)-1,4,7,10-tetraazacyclododecan-1-yl]acetamido}methyl)cyclohexane-1-carbonyl]-L-alanyl}-N^2^-{[(1S)-1,3-dicarboxypropyl]carbamoyl}-L-lysine*

Tri-tert-butyl (3S,10S,14S)-3-[(isoquinolin-3-yl)methyl]-1,4,12-trioxo-1-[(1r,4S)-4-({2-[4,7,10-tris(2-tert-butoxy-2-oxoethyl)-1,4,7,10-tetraazacyclododecan-1-yl]acetamido}methyl)cyclohexyl]-2,5,11,13-tetraazahexadecane-10,14,16-tricarboxylate (25.0 mg, 18.1 µmol) was solubilized in DCM (580 µL), TFA (280 µL, 3.6 mmol) was added, and the mixture was stirred under argon atmosphere at room temperature over the weekend. The mixture was evaporated and purified by preparative HPLC (C18, acetonitrile/water with 0.1% formic acid) to give 5.00 mg (95% purity, 25% yield) of the target compound. LC-MS (Method 1): Rt = 0.54 min; MS (ESIpos): m/z = 1044 [M+H]^+^. ^1^H-NMR (400 MHz, DMSO-*d*_6_) δ ppm 9.23 (s, 1 H) 8.07 (br d, *J*=8.11 Hz, 3 H) 7.76 - 7.88 (m, 2 H) 7.73 (t, *J*=7.48 Hz, 1 H) 7.58 - 7.63 (m, 2 H) 6.19 - 6.40 (m, 2 H) 4.62 - 4.74 (m, 1 H) 3.93 - 4.13 (m, 2 H) 3.41 - 3.45 (m, 6 H) 2.85 - 3.12 (m, 19 H) 2.57 - 2.65 (m, 2 H) 2.19 - 2.28 (m, 2 H) 1.82 - 2.09 (m, 3 H) 1.50 - 1.81 (m, 6 H) 1.36 - 1.48 (m, 3 H) 1.14 - 1.34 (m, 10 H) 0.67 - 0.87 (m, 3 H).

**Synthesis of Compound D**

### *Intermediate 1 for Compound D*

*Methyl isoquinoline-7-carboxylate*

To a solution of 7-bromoisoquinoline (15.0 g, 72.1 mmol) in MeOH (150 ml), 1,1'-bis(diphenylphosphino)ferrocenepalladium(II) chloride (5.28 g, 7.21 mmol; CAS-RN:[72287-26-4]) and trimethylamine (20 mL, 140 mmol) were added. The reaction mixture was stirred under carbon monoxide atmosphere (50 Psi) at 70 °C for 16 h. The precipitate was removed by filtration. The filtrate was concentrated under reduced pressure. The residue was purified by flash chromatography (SiO_2_, petroleum ether / EtOAc gradient 10-20%) to give 11.5 g (85% yield) of the target compound. ^1^H-NMR (400 MHz, CDCl_3_): δ ppm = 9.35 (s, 1H), 8.72 (s, 1H), 8.62 (dd, 1H), 8.28 (d, 1H), 7.86 (d, 1H), 7.69 (d, 1H), 4.01 (s, 3H).

### *Intermediate 2 for Compound D*

*(Isoquinolin-7-yl)MeOH*

To a mixture of methyl isoquinoline-7-carboxylate (8.50 g, 45.4 mmol) in THF (85 mL), was added lithium aluminum hydride (1.72 g, 45.4 mmol; CAS-RN:[16853-85-3]) at 0 °C. The reaction mixture was stirred at room temperature for 2 h. EtOAc and sodium potassium tartrate aqueous solution were added to the reaction mixture. The mixture was stirred at room temperature overnight. The mixture was extracted with EtOAc. The organic phase was dried and concentrated under reduced pressure. The residue was purified by flash chromatography (SiO_2_, petroleum ether / EtOAc gradient 20-50%) to give 3.90 g (54% yield) of the target compound.

### *Intermediate 3 for Compound D*

*Isoquinoline-7-carbaldehyde*

To a solution of (isoquinolin-7-yl)MeOH (3.90 g, 24.5 mmol) in acetonitrile (50 ml), manganese(IV) oxide (10.6 g, 122 mmol) was added. The reaction was stirred at 80 °C for 16 h. The solid was removed by filtration. The filtrate was concentrated under reduced pressure to give a residue. The residue was purified by flash chromatography (SiO_2_, petroleum ether / EtOAc gradient 20-50%) to give 2.41 g (63% yield) of the target compound. ^1^H-NMR (400 MHz, CDCl_3_): δ ppm = 10.23 (s, 1H), 9.47 (s, 1H), 8.72 (d, 1H), 8.53 (s, 1H), 8.22 (dd, 1H), 7.98 (d, 1H), 7.78 (d, 1H).

### *Intermediate 4 for Compound D*

*Methyl (2Z)-2-{[(benzyloxy)carbonyl]amino}-3-(isoquinolin-7-yl)prop-2-enoate*

1,8-Diazabicyclo(5.4.0)undec-7-ene (6.0 mL, 40 mmol) was added dropwise to a solution of methyl {[(benzyloxy)carbonyl]amino}(dimethoxyphosphoryl)acetate (13.4 g, 40.5 mmol) in DCM (100 mL). After stirring at room temperature for 10 min, a solution of isoquinoline-7-carbaldehyde (5.30 g, 33.7 mmol) in DCM (50 mL) was added. The reaction was stirred at room temperature for 2 h. The reaction solvent was removed under reduced pressure. The residue was diluted with EtOAc. The solution was washed with 1 M HCl and brine. The organic phase was dried. The filtrate was concentrated and purified by flash chromatography (SiO_2_, petroleum ether / EtOAc gradient 10-50%) to give 8.50 g (70% yield) of the target compound. LC-MS (Method 3): R_t_ = 0.608 min; MS (ESIpos): m/z =363.1 [M+H]^+^.

### *Intermediate 5 for Compound D*

*Methyl (2R)-2-{[(benzyloxy)carbonyl]amino}-3-(isoquinolin-7-yl)propanoate*

To a solution of methyl (2Z)-2-{[(benzyloxy)carbonyl]amino}-3-(isoquinolin-7-yl)prop-2-enoate (3.20 g, 8.83 mmol) in MeOH, (R)-[Rh(COD)(MaxPHOS)]BF4 (150 mL) was added. The reaction mixture was stirred under hydrogen atmosphere (1 MPa) at room temperature for 96 h. The reaction mixture was evaporated under reduced pressure. The residue was purified by flash chromatography (SiO_2_, petroleum ether / EtOAc gradient 10-50%) to give 1.20 g (91% purity, 34% yield) and 500 mg (50% purity, 8% yield) of the target compound. LC-MS (Method 3): R_t_ = 0.623 min; MS (ESIpos): m/z =365.0 [M+H]^+^.

### *Intermediate 6 for Compound D*

*(2R)-2-{[(benzyloxy)carbonyl]amino}-3-(isoquinolin-7-yl)propanoic acid*

To a cooled solution (0 °C) of methyl (2R)-2-{[(benzyloxy)carbonyl]amino}-3-(isoquinolin-7-yl)propanoate (3.30 g, 9.06 mmol) in THF (30 mL), lithium hydroxide (5.4 mL, 2.0 M, 11 mmol; CAS-RN:[1310-65-2]) was added. The reaction mixture was stirred at room temperature for 16 h. The reaction solution was concentrated under reduced pressure and dissolved in MeOH. The pH of the mixture was adjusted to 5 with formic acid. The mixture was concentrated and purified by preparative HPLC (C18, acetonitrile/water with 0.1% formic acid) to give 2.00 g (63% yield) of the target compound. LC-MS (Method 2): R_t_ = 0.685 min; MS (ESIpos): m/z =351.1 [M+H]^+^. ^1^H-NMR (400 MHz, DMSO-*d_6_*): δ ppm = 9.23 (s, 1H), 8.48 (d, 1H), 7.96 (s, 1H), 7.90 (d, 1H), 7.83-7.68 (m, 3H), 7.29-7.07 (m, 5H), 4.94 (s, 2H), 4.39-4.31 (m, 1H), 3.27-3.22 (m, 1H), 3.07 (dd, 1H).

### *Intermediate* *7 for Compound D*

*Tri-tert-butyl (5R,12S,16S)-5-[(isoquinolin-7-yl)methyl]-3,6,14-trioxo-1-phenyl-2-oxa-4,7,13,15-tetraazaoctadecane-12,16,18-tricarboxylate*

Di-tert-butyl N-{[(2S)-6-amino-1-tert-butoxy-1-oxohexan-2-yl]carbamoyl}-L-glutamate (1.11 g, 2.28 mmol) and (2R)-2-{[(benzyloxy)carbonyl]amino}-3-(isoquinolin-7-yl)propanoic acid (800 mg, 2.28 mmol) were solubilized in DMF (18 mL), 4-methylmorpholine (630 µL, 5.7 mmol, CAS-RN: 109-02-4) and HATU (1.22 g, 3.20 mmol) were added, and the mixture was stirred under argon atmosphere at room temperature overnight. The mixture was evaporated and purified by preparative HPLC (C18, acetonitrile/water with 0.1% formic acid) to give 460 mg (98% purity, 24% yield) of the target compound. LC-MS (Method 1): R_t_ = 1.28 min; MS (ESIpos): m/z = 821 [M+H]^+^. ^1^H-NMR (400 MHz, DMSO-*d*_6_) δ ppm 9.20 (s, 1 H) 8.46 (d, *J*=5.83 Hz, 1 H) 8.04 (br t, *J*=5.45 Hz, 1 H) 7.92 (s, 1 H) 7.88 (d, *J*=8.36 Hz, 1 H) 7.79 (d, *J*=5.58 Hz, 1 H) 7.71 (dd, *J*=8.62, 1.52 Hz, 1 H) 7.59 (d, *J*=8.62 Hz, 1 H) 7.12 - 7.24 (m, 5 H) 6.27 (dd, *J*=19.64, 8.24 Hz, 2 H) 4.84 - 4.97 (m, 2 H) 4.32 (td, *J*=9.25, 4.82 Hz, 1 H) 4.04 (td, *J*=8.49, 5.32 Hz, 1 H) 3.93 (td, *J*=8.05, 5.45 Hz, 1 H) 3.16 (dd, *J*=13.43, 4.56 Hz, 1 H) 2.90 - 3.11 (m, 3 H) 2.12 - 2.30 (m, 2 H) 1.80 - 1.92 (m, 1 H) 1.61 - 1.73 (m, 1 H) 1.49 - 1.60 (m, 1 H) 1.42 - 1.49 (m, 1 H) 1.38 (d, *J*=1.27 Hz, 28 H) 1.22 (br d, *J*=7.60 Hz, 3 H).

### *Intermediate* *8 for Compound D*

*Di-tert-butyl (2S)-2-({[(2S)-6-{[(2R)-2-amino-3-(isoquinolin-7-yl)propanoyl]amino}-1-tert-butoxy-1-oxohexan-2-yl]carbamoyl}amino)pentanedioate*

Tri-tert-butyl (5R,12S,16S)-5-[(isoquinolin-7-yl)methyl]-3,6,14-trioxo-1-phenyl-2-oxa-4,7,13,15-tetraazaoctadecane-12,16,18-tricarboxylate (460 mg, 561 µmol) was solubilized in MeOH (4.5 mL). Palladium on carbon (59.7 mg, 10% purity, 56.1 µmol) was added and the mixture was purged with hydrogen. The mixture was stirred under hydrogen atmosphere at room temperature for 8 h. The mixture was filtered over Celite, washed with MeOH, and evaporated to give 330 mg (91% purity, 78% yield) of the target compound. LC-MS (Method 1): R_t_ = 1.29 min; MS (ESIpos): m/z = 686 [M+H]^+^.

### *Intermediate* *9 for Compound D*

*Tri-tert-butyl (3R,10S,14S)-1-{(1r,4S)-4-[({[(9H-fluoren-9-yl)methoxy]carbonyl} amino)methyl]cyclohexyl}-3-[(isoquinolin-7-yl)methyl]-1,4,12-trioxo-2,5,11,13-tetraazahexadecane-10,14,16-tricarboxylate*

(1r,4r)-4-[({[(9H-fluoren-9-yl)methoxy]carbonyl}amino)methyl]cyclohexane-1-carboxylic acid (183 mg, 482 µmol) was solubilized in DMF (6.7 mL), 4-methylmorpholine (140 µL, 1.3 mmol, CAS-RN: 109-02-4) and HATU (183 mg, 482 µmol) were added, and stirred at room temperature for 20 min. di-tert-butyl (2S)-2-({[(2S)-6-{[(2R)-2-amino-3-(isoquinolin-7-yl)propanoyl]amino}-1-tert-butoxy-1-oxohexan-2-yl]carbamoyl}amino)pentanedioate (330 mg, 91% purity, 438 µmol) was added and the mixture was stirred under argon at room temperature overnight. The mixture was filtered and purified by preparative HPLC (C18, acetonitrile/water with 0.1% formic acid) to give 150 mg (95% purity, 31% yield) of the target compound. LC-MS (Method 1): R_t_ = 1.42 min; MS (ESIpos): m/z = 1049 [M+H]^+^.

### *Intermediate* *10 for Compound D*

*Tri-tert-butyl (3R,10S,14S)-1-[(1r,4S)-4-(aminomethyl)cyclohexyl]-3-[(isoquinolin-7-yl)methyl]-1,4,12-trioxo-2,5,11,13-tetraazahexadecane-10,14,16-tricarboxylate*

Tri-tert-butyl (3R,10S,14S)-1-{(1r,4S)-4-[({[(9H-fluoren-9-yl)methoxy]carbonyl}amino) methyl]cyclohexyl}-3-[(isoquinolin-7-yl)methyl]-1,4,12-trioxo-2,5,11,13-tetraazahexadecane-10,14,16-tricarboxylate (150 mg, 95% purity, 136 µmol) was solubilized in DMF (2.1 mL), piperidine (270 µL, 2.7 mmol) was added, and the mixture was stirred under argon atmosphere at room temperature overnight. The mixture was evaporated and purified by preparative HPLC (C18, acetonitrile/water with 0.1% formic acid) to give 84.0 mg (98% purity, 73% yield) of the target compound.

LC-MS (Method 1): R_t_ = 0.96 min; MS (ESIpos): m/z = 826 [M+H]^+^. ^1^H-NMR (400 MHz, DMSO-*d*_6_) δ ppm 9.19 (s, 1 H) 8.40 - 8.47 (m, 2 H) 7.98 - 8.05 (m, 2 H) 7.84 - 7.90 (m, 2 H) 7.77 (d, *J*=5.83 Hz, 1 H) 7.66 (dd, *J*=8.49, 1.39 Hz, 1 H) 6.22 - 6.37 (m, 2 H) 4.56 (td, *J*=9.06, 5.20 Hz, 1 H) 4.03 (td, *J*=8.49, 5.32 Hz, 1 H) 3.87 - 3.94 (m, 1 H) 3.15 (br dd, *J*=13.43, 5.07 Hz, 2 H) 2.91 - 3.09 (m, 3 H) 2.13 - 2.30 (m, 2 H) 1.97 - 2.11 (m, 1 H) 1.80 - 1.92 (m, 1 H) 1.59 - 1.79 (m, 4 H) 1.42 - 1.58 (m, 3 H) 1.38 (m, 28 H) 1.23 (br d, *J*=5.83 Hz, 7 H) 0.94 - 1.07 (m, 1 H) 0.72 - 0.92 (m, 2 H).

### *Intermediate* *11 for Compound D*

*Tri-tert-butyl (3R,10S,14S)-3-[(isoquinolin-7-yl)methyl]-1,4,12-trioxo-1-[(1r,4S)-4-({2-[4,7,10-tris(2-tert-butoxy-2-oxoethyl)-1,4,7,10-tetraazacyclododecan-1-yl]acetamido}methyl)cyclohexyl]-2,5,11,13-tetraazahexadecane-10,14,16-tricarboxylate*

[4,7,10-tris(2-tert-butoxy-2-oxoethyl)-1,4,7,10-tetraazacyclododecan-1-yl]acetic acid (136 mg, 238 µmol; CAS-RN:[137076-54-1]), [(1H-benzotriazol-1-yl)oxy](dimethylamino)-N,N-dimethylmethaniminium hexafluoridophosphate(1-) (89.0 mg, 235 µmol; CAS-RN:94790-37-1), and N,N-diisopropylethylamine (30 µL, 180 µmol) were stirred in DMF (6.2 mL) at room temperature for 10 min. Tri-tert-butyl (3R,10S,14S)-1-[(1r,4S)-4-(aminomethyl)cyclohexyl]-3-[(isoquinolin-7-yl)methyl]-1,4,12-trioxo-2,5,11,13-tetraazahexadecane-10,14,16-tricarboxylate (49.0 mg, 59.4 µmol) was added and the mixture was stirred at room temperature overnight. The mixture was evaporated and purified by preparative HPLC (C18, acetonitrile/water with 0.1% formic acid) to give 70.0 mg (95% purity, 81% yield) of the target compound. LC-MS (Method 1): R_t_ = 1.26 min; MS (ESIpos): m/z = 1381 [M+H]^+^.

### *Compound D*

*(3R,10S,14S)-3-[(isoquinolin-7-yl)methyl]-1,4,12-trioxo-1-[(1r,4S)-4-({2-[4,7,10-tris(carboxymethyl)-1,4,7,10-tetraazacyclododecan-1-yl]acetamido}methyl) cyclohexyl]-2,5,11,13-tetraazahexadecane-10,14,16-tricarboxylic acid*

Tri-tert-butyl (3R,10S,14S)-3-[(isoquinolin-7-yl)methyl]-1,4,12-trioxo-1-[(1r,4S)-4-({2-[4,7,10-tris(2-tert-butoxy-2-oxoethyl)-1,4,7,10-tetraazacyclododecan-1-yl]acetamido}methyl)cyclohexyl]-2,5,11,13-tetraazahexadecane-10,14,16-tricarboxylate (4.00 mg, 2.90 µmol) was solubilized in DCM (1.0 mL), TFA (510 µL) was added and the mixture was stirred under argon overnight at room temperature. The mixture was evaporated and purified by preparative HPLC (C18, acetonitrile/water with 0.1% formic acid) to give 2.50 mg (90% purity, 74% yield) of the target compound. LC-MS (Method 1): R_t_ = 0.48 min; MS (ESIpos): m/z = 1042 [M-H]^+^.  ^1^H-NMR (400 MHz, DMSO-*d*_6_) δ ppm 9.20 (s, 1 H) 8.43 (d, *J*=5.58 Hz, 1 H) 8.03 - 8.12 (m, 2 H) 7.98 (br d, *J*=1.01 Hz, 1 H) 7.82 - 7.92 (m, 2 H) 7.77 (d, *J*=5.58 Hz, 1 H) 7.67 (dd, *J*=8.62, 1.27 Hz, 1 H) 6.32 (br dd, *J*=7.35, 5.32 Hz, 2 H) 4.44 - 4.56 (m, 1 H) 4.03 - 4.13 (m, 1 H) 3.91 - 4.02 (m, 1 H) 3.40 - 3.48 (br s, 9 H) 3.14 - 3.20 (m, 3 H) 2.85 - 3.12 (m, 20 H) 2.16 - 2.29 (m, 2 H) 1.97 - 2.09 (m, 2 H) 1.82 - 1.95 (m, 1 H) 1.54 - 1.78 (m, 6 H) 1.12 - 1.51 (m, 11 H) 0.72 - 0.89 (m, 2 H).

**Synthesis of Compound E**

### *Intermediate 1 for Compound E*

*Tri-tert-butyl (4S,11S,15S)-1-[4-({[(9H-fluoren-9-yl)methoxy]carbonyl}amino)phenyl]-4-[(naphthalen-2-yl)methyl]-2,5,13-trioxo-3,6,12,14-tetraazaheptadecane-11,15,17-tricarboxylate*

Di-tert-butyl (2S)-2-({[(2S)-6-{[(2S)-2-amino-3-(naphthalen-2-yl)propanoyl]amino}-1-tert-butoxy-1-oxohexan-2-yl]carbamoyl}amino)pentanedioate (319 mg, 90% purity, 419 µmol) and [4-({[(9H-fluoren-9-yl)methoxy]carbonyl}amino)phenyl]acetic acid (130 mg, 349 µmol) were solubilized in DMF (2.7 ml), 4-methylmorpholine (260 µL, 1.0 mmol, CAS-RN: 109-02-4) and COMU (150 mg, 349 µmol) were added, and the mixture was stirred under argon atmosphere at room temperature for 2 h. The mixture was diluted with brine and extracted 3 times with DCM. The combined organic phases were dried and evaporated and purified by flash chromatography (SiO2, DCM/Ethanol gradient 0-10%) to give 219 mg (80% purity, 40% yield) of the target compound. LC-MS (Method 1): R_t_ = 1.64 min; MS (ESIpos): m/z = 1041 [M+H]^+^. ^1^H-NMR (400 MHz, DMSO-*d*_6_) δ ppm 8.26 (d, *J*=8.62 Hz, 1 H) 8.02 (t, *J*=5.45 Hz, 1 H) 7.91 (d, *J*=7.35 Hz, 2 H) 7.81 - 7.85 (m, 1 H) 7.72 - 7.78 (m, 3 H) 7.64 (s, 1 H) 7.39 - 7.49 (m, 3 H) 7.30 - 7.39 (m, 3 H) 7.16 - 7.30 (m, 2 H) 6.96 (br d, *J*=8.11 Hz, 1 H) 6.26 (dd, *J*=19.39, 8.24 Hz, 2 H) 4.54 (br d, *J*=5.32 Hz, 1 H) 4.46 (br d, *J*=6.84 Hz, 1 H) 4.25 - 4.35 (m, 1 H) 4.03 (td, *J*=8.43, 5.20 Hz, 1 H) 3.85 - 3.96 (m, 1 H) 3.52 - 3.60 (m, 4 H) 3.04 - 3.10 (m, 5 H) 2.89 - 2.98 (m, 1 H) 2.74 (s, 2 H) 2.15 - 2.29 (m, 1 H) 1.80 - 1.93 (m, 1 H) 1.60 - 1.72 (m, 1 H) 1.48 - 1.57 (m, 1 H) 1.42 - 1.47 (m, 1 H) 1.38 (d, *J*=1.77 Hz, 27 H) 1.13 - 1.32 (m, 4 H).

### *Intermediate 2 for Compound E*

*Tri-tert-butyl (4S,11S,15S)-1-(4-aminophenyl)-4-[(naphthalen-2-yl)methyl]-2,5,13-trioxo-3,6,12,14-tetraazaheptadecane-11,15,17-tricarboxylate*

Tri-tert-butyl (4S,11S,15S)-1-[4-({[(9H-fluoren-9-yl)methoxy]carbonyl}amino)phenyl]-4-[(naphthalen-2-yl)methyl]-2,5,13-trioxo-3,6,12,14-tetraazaheptadecane-11,15,17-tricarboxylate (219 mg, 85% purity, 179 µmol) was solubilized in DMF (1.4 mL), piperidine (350 µL, 3.6 mmol) was added, and the mixture was stirred under argon atmosphere at room temperature overnight. The mixture was evaporated and purified by preparative HPLC (C18, acetonitrile/water with 0.1% formic acid) to give 126 mg (90% purity, 77% yield) of the target compound. LC-MS (Method 1): R_t_ = 1.40 min; MS (ESIpos): m/z = 819 [M+H]^+^. ^1^H-NMR (400 MHz, DMSO-*d*_6_) δ ppm 8.06 (d, *J*=8.36 Hz, 1 H), 7.98 (t, *J*=5.58 Hz, 1 H), 7.82 - 7.91 (m, 2 H), 7.73 - 7.80 (m, 2 H), 7.65 (s, 1 H), 7.30 - 7.51 (m, 4 H), 6.71 (d, *J*=8.36 Hz, 2 H), 6.22 - 6.36 (m, 4 H), 4.83 (s, 2 H), 4.52 (td, *J*=8.55, 5.45 Hz, 1 H), 4.03 (td, *J*=8.49, 5.07 Hz, 1 H), 3.92 (td, *J*=7.98, 5.58 Hz, 1 H), 3.00 - 3.11 (m, 2 H), 2.88 - 2.99 (m, 2 H), 2.12 - 2.29 (m, 2 H), 1.79 - 1.91 (m, 1 H), 1.60 - 1.72 (m, 1 H), 1.42 - 1.59 (m, 2 H), 1.38 (d, *J*=1.77 Hz, 27 H), 1.13 - 1.31 (m, 4 H).

### *Intermediate 3 for Compound E*

*Tri-tert-butyl (4S,11S,15S)-4-[(naphthalen-2-yl)methyl]-2,5,13-trioxo-1-(4-{2-[4,7,10-tris(2-tert-butoxy-2-oxoethyl)-1,4,7,10-tetraazacyclododecan-1-yl]acetamido}phenyl)-3,6,12,14-tetraazaheptadecane-11,15,17-tricarboxylate*

N-[9,12-bis(2-tert-butoxy-2-oxoethyl)-2,2,6-trimethyl-4-oxo-3-oxa-6,9,12-triazatetradecan-14-yl]-N-ethylglycine (132 mg, 225 µmol), [(1H-benzotriazol-1-yl)oxy](dimethylamino)-N,N-dimethylmethaniminium hexafluoridophosphate(1-) (84.2 mg, 222 µmol; CAS-RN:94790-37-1) and N,N-diisopropylethylamine (36 µL, 220 µmol) were stirred in DMF (2 mL) at room temperature for 10 min. Tri-tert-butyl (4S,11S,15S)-1-(4-aminophenyl)-4-[(naphthalen-2-yl)methyl]-2,5,13-trioxo-3,6,12,14-tetraazaheptadecane-11,15,17-tricarboxylate (57.5 mg, 80% purity, 56.2 µmol) was added and the mixture was stirred at room temperature overnight. The mixture was evaporated and purified by preparative HPLC (C18, acetonitrile/water with 0.1% formic acid) to give 45.0 mg (70% purity, 41% yield) of the target compound. LC-MS (Method 1): R_t_ = 1.26 min; MS (ESIpos): m/z = 1373 [M+H]^+^. ^1^H-NMR (400 MHz, DMSO-*d*_6_) δ ppm 8.20 - 8.31 (m, 1 H), 8.04 (br t, *J*=5.32 Hz, 1 H), 7.80 - 7.87 (m, 1 H), 7.76 (br d, *J*=8.36 Hz, 2 H), 7.65 (s, 1 H), 7.42 - 7.50 (m, 2 H), 7.26 - 7.41 (m, 4 H), 6.99 - 7.06 (m, 2 H), 6.26 (dd, *J*=19.52, 8.36 Hz, 2 H), 4.48 - 4.59 (m, 1 H), 3.53 - 4.24 (m, 11 H), 2.83 - 3.22 (m, 18 H), 2.13 - 2.30 (m, 2 H), 1.86 (td, *J*=13.62, 6.97 Hz, 1 H), 1.60 - 1.71 (m, 2 H), 1.32 - 1.50 (m, 60 H), 1.12 - 1.21 (m, 2 H).

### *Compound E*

*(4S,11S,15S)-4-[(naphthalen-2-yl)methyl]-2,5,13-trioxo-1-(4-{2-[4,7,10-tris(carboxymethyl)-1,4,7,10-tetraazacyclododecan-1-yl]acetamido}phenyl)-3,6,12,14-tetraazaheptadecane-11,15,17-tricarboxylic acid*

Tri-tert-butyl (4S,11S,15S)-4-[(naphthalen-2-yl)methyl]-2,5,13-trioxo-1-(4-{2-[4,7,10-tris(2-tert-butoxy-2-oxoethyl)-1,4,7,10-tetraazacyclododecan-1-yl]acetamido}phenyl)-3,6,12,14-tetraazaheptadecane-11,15,17-tricarboxylate (40.0 mg, 29.1 µmol) was solubilized in DCM (560 µL), TFA (166 mg, 1.46 mmol) was added, and the mixture was stirred under argon atmosphere at room temperature overnight. The mixture was evaporated and purified by preparative HPLC (C18, acetonitrile/water with 0.1% formic acid) to give 3.80 mg (90% purity, 11% yield) of the target compound. LC-MS (Method 1): R_t_ = 0.70 min; MS (ESIpos): m/z = 1037 [M+H]^+^. ^1^H-NMR (400 MHz, DMSO-*d*_6_) δ ppm 9.87 - 10.23 (m, 1 H), 8.23 - 8.40 (m, 1 H), 7.74 - 7.90 (m, 3 H), 7.65 - 7.70 (m, 1 H), 7.41 - 7.52 (m, 3 H), 7.32 - 7.39 (m, 1 H), 7.01 (d, *J*=8.36 Hz, 1 H), 6.20 - 6.38 (m, 1 H), 4.48 - 4.61 (m, 1 H), 3.97 - 4.13 (m, 2 H), 3.48 - 3.61 (m, 8 H), 2.70 - 3.21 (m, 20 H), 2.19 - 2.28 (m, 2 H), 1.85 - 1.96 (m, 1 H), 1.64 - 1.79 (m, 1 H), 1.51 - 1.62 (m, 1 H), 1.37 - 1.49 (m, 1 H), 1.14 - 1.33 (m, 7 H).

**Synthesis of Compound F**

### *Intermediate 1 for Compound F*

*Tri-tert-butyl (3S,10S,14S)-1-{4-[({[(9H-fluoren-9-yl)methoxy]carbonyl}amino)methyl] phenyl}-3-[(naphthalen-2-yl)methyl]-1,4,12-trioxo-2,5,11,13-tetraazahexadecane-10,14,16-tricarboxylate*

Di-tert-butyl (2S)-2-({[(2S)-6-{[(2S)-2-amino-3-(naphthalen-2-yl)propanoyl]amino}-1-tert-butoxy-1-oxohexan-2-yl]carbamoyl}amino)pentanedioate (290 mg, 95% purity, 402 µmol) and 4-[({[(9H-fluoren-9-yl)methoxy]carbonyl}amino)methyl]benzoic acid (150 mg, 402 µmol) were solubilized in DMF (3.1 mL), 4-methylmorpholine (180 µL, 1.6 mmol, CAS-RN: 109-02-4) and HATU (229 mg, 603 µmol) were added, and the mixture was stirred under argon atmosphere at room temperature for 1 h. The mixture was evaporated and purified by preparative HPLC (C18, acetonitrile/water with 0.1% formic acid) to give 109 mg (95% purity, 25% yield) of the target compound. LC-MS (Method 1): Rt = 1.62 min; MS (ESIpos): m/z = 1041 [M+H]^+^. ^1^H-NMR (400 MHz, DMSO-*d*_6_) δ ppm 8.56 (br d, *J*=7.86 Hz, 1 H), 8.05 - 8.14 (m, 1 H), 7.85 - 7.92 (m, 3 H), 7.76 - 7.85 (m, 4 H), 7.66 - 7.75 (m, 4 H), 7.51 (br d, *J*=9.12 Hz, 1 H), 7.38 - 7.47 (m, 4 H), 7.29 - 7.37 (m, 2 H), 7.23 (br d, *J*=7.86 Hz, 3 H), 6.28 (dd, *J*=13.69, 8.36 Hz, 2 H), 4.69 - 4.81 (m, 1 H), 4.35 (d, *J*=6.59 Hz, 2 H), 4.15 - 4.26 (m, 3 H), 3.99 - 4.09 (m, 1 H), 3.89 - 3.97 (m, 1 H), 2.94 - 3.27 (m, 3 H), 2.14 - 2.28 (m, 2 H), 1.80 - 1.93 (m, 1 H), 1.43 - 1.70 (m, 3 H), 1.38 (s, 29 H), 1.24 (m, 2 H).

### *Intermediate 2 for Compound F*

*Tri-tert-butyl (3S,10S,14S)-1-[4-(aminomethyl)phenyl]-3-[(naphthalen-2-yl)methyl]-1,4,12-trioxo-2,5,11,13-tetraazahexadecane-10,14,16-tricarboxylate*

Tri-tert-butyl (3S,10S,14S)-1-{4-[({[(9H-fluoren-9-yl)methoxy]carbonyl}amino)methyl] phenyl}-3-[(naphthalen-2-yl)methyl]-1,4,12-trioxo-2,5,11,13-tetraazahexadecane-10,14,16-tricarboxylate (267 mg, 80% purity, 205 µmol) was solubilized in DMF (2.1 mL), piperidine (390 µL, 2.1 mmol) was added, and the mixture was stirred under argon atmosphere at room temperature overnight. The mixture was evaporated and purified by preparative HPLC (C18, acetonitrile/water with 0.1% formic acid) to give 56.0 mg (70% purity, 23% yield) of the target compound. LC-MS (Method 1): R_t_ = 1.23 min; MS (ESIpos): m/z = 819 [M+H]^+^. ^1^H-NMR (400 MHz, DMSO-*d*_6_) δ ppm 8.58 (d, *J*=8.62 Hz, 1 H), 8.31 (s, 1 H), 8.10 - 8.18 (m, 1 H), 7.95 (s, 1 H), 7.72 - 7.87 (m, 6 H), 7.34 - 7.56 (m, 5 H), 6.29 (dd, *J*=13.94, 8.36 Hz, 2 H), 4.68 - 4.82 (m, 1 H), 4.03 (td, *J*=8.55, 5.45 Hz, 1 H), 3.88 - 3.98 (m, 2 H), 2.96 - 3.18 (m, 5 H), 2.11 - 2.29 (m, 2 H), 1.85 (dt, *J*=13.69, 7.10 Hz, 1 H), 1.42 - 1.72 (m, 4 H), 1.38 (s, 30 H).

### *Intermediate 3 for Compound F*

*Tri-tert-butyl (3S,10S,14S)-3-[(naphthalen-2-yl)methyl]-1,4,12-trioxo-1-[4-({2-[4,7,10-tris(2-tert-butoxy-2-oxoethyl)-1,4,7,10-tetraazacyclododecan-1-yl]acetamido}methyl)phenyl]-2,5,11,13-tetraazahexadecane-10,14,16-tricarboxylate*

[4,7,10-tris(2-tert-butoxy-2-oxoethyl)-1,4,7,10-tetraazacyclododecan-1-yl]acetic acid (89.6 mg, 156 µmol), [(1H-benzotriazol-1-yl)oxy](dimethylamino)-N,N-dimethylmethaniminium hexafluoridophosphate(1-) (58.6 mg, 155 µmol; CAS-RN:94790-37-1), and N,N-diisopropylethylamine (28 µL, 160 µmol) were stirred in DMF (1 mL) at room temperature for 10 min. Tri-tert-butyl (3S,10S,14S)-1-[4-(aminomethyl)phenyl]-3-[(naphthalen-2-yl)methyl]-1,4,12-trioxo-2,5,11,13-tetraazahexadecane-10,14,16-tricarboxylate (40.0 mg, 80% purity, 39.1 µmol) was added and the mixture was stirred at room temperature for 5 days. The mixture was evaporated and purified by preparative HPLC (C18, acetonitrile/water with 0.1% formic acid) to give 35.0 mg (85% purity, 55% yield) of the target compound. LC-MS (Method 1): R_t_ = 1.39 min; MS (ESIpos): m/z = 1373 [M+H]^+^. ^1^H-NMR (400 MHz, DMSO-*d*_6_) δ ppm 8.72 - 8.94 (m, 1 H), 8.47 - 8.61 (m, 1 H), 8.09 - 8.23 (m, 1 H), 7.78 (br d, *J*=7.10 Hz, 6 H), 7.23 - 7.56 (m, 6 H), 6.28 (dd, *J*=13.69, 8.36 Hz, 2 H), 4.69 - 4.81 (m, 1 H), 4.28 - 4.38 (m, 2 H), 3.87 - 4.16 (m, 4 H), 3.70 - 3.80 (m, 1 H), 3.45 - 3.63 (m, 4 H), 2.71 - 3.28 (m, 21 H), 2.13 - 2.31 (m, 2 H), 1.86 (td, *J*=13.69, 7.35 Hz, 1 H), 1.52 - 1.72 (m, 3 H), 1.30 - 1.51 (m, 51 H), 1.18 - 1.30 (m, 4 H)

### *Compound F*

*(3S,10S,14S)-3-[(naphthalen-2-yl)methyl]-1,4,12-trioxo-1-[4-({2-[4,7,10-tris(carboxymethyl)-1,4,7,10-tetraazacyclododecan-1-yl]acetamido}methyl)phenyl]-2,5,11,13-tetraazahexadecane-10,14,16-tricarboxylic acid*

Tri-tert-butyl (3S,10S,14S)-3-[(naphthalen-2-yl)methyl]-1,4,12-trioxo-1-[4-({2-[4,7,10-tris(2-tert-butoxy-2-oxoethyl)-1,4,7,10-tetraazacyclododecan-1-yl]acetamido}methyl)phenyl]-2,5,11,13-tetraazahexadecane-10,14,16-tricarboxylate (36.0 mg, 26.2 µmol) was solubilized in DCM (840 µL), TFA (2.0 mL, 26 mmol) was added, and the mixture was stirred under argon atmosphere at room temperature over the weekend. The mixture was evaporated and purified by preparative HPLC (C18, acetonitrile/water with 0.1% formic acid) to give 6.00 mg (95% purity, 21% yield) of the target compound. LC-MS (Method 1): R_t_ = 0.73 min; MS (ESIpos): m/z = 1037 [M+H]^+^. ^1^H-NMR (400 MHz, DMSO-*d*_6_) δ ppm 8.55 - 8.79 (m, 2 H), 8.05 - 8.25 (m, 1 H), 7.92 - 8.02 (m, 2 H), 7.66 - 7.89 (m, 6 H), 7.21 - 7.59 (m, 6 H), 6.33 (br d, *J*=1.01 Hz, 2 H), 4.69 - 4.83 (m, 1 H), 4.21 - 4.40 (m, 2 H), 3.99 - 4.10 (m, 2 H), 2.63 - 3.28 (m, 29 H), 1.09 - 1.95 (m, 12 H).

**Synthesis of Compound G**

### *Intermediate 1 for Compound G*

*5-[({[(9H-fluoren-9-yl)methoxy]carbonyl}amino)methyl]pyridine-2-carboxylic acid*

5-(aminomethyl)pyridine-2-carboxylic acid (410 mg, 97% purity, 2.61 mmol) was solubilized in 1,4-dioxane (5.0 mL), sodium carbonate (5.8 mL, 2.0 M, 12 mmol) and (9H-fluoren-9-yl)methyl carbonochloridate (751 mg, 2.90 mmol) were added, and the mixture was stirred at room temperature for 2 days. HCl (20 mL, 2.0 M) was added dropwise and extracted with DCM. The organic phase was washed with brine, dried, evaporated, and purified by preparative HPLC (C18, acetonitrile/water with 0.1% formic acid) to give 160 mg (95% purity, 16% yield) of the target compound. LC-MS (Method 1): R_t_ = 0.99 min; MS (ESIpos): m/z = 375 [M+H]^+^ . ^1^H-NMR (400 MHz, DMSO-*d*_6_) δ ppm 8.57 (d, *J*=1.77 Hz, 1 H), 7.89 (d, *J*=7.60 Hz, 2 H), 7.75 (dd, *J*=7.98, 2.15 Hz, 1 H), 7.68 (d, *J*=7.60 Hz, 2 H), 7.38 - 7.46 (m, 2 H), 7.29 - 7.35 (m, 2 H), 4.38 (d, *J*=6.59 Hz, 2 H), 4.28 (d, *J*=6.08 Hz, 2 H), 4.19 - 4.26 (m, 1 H), 4.10 (q, *J*=5.15 Hz, 1 H), 3.17 (d, *J*=5.07 Hz, 2 H).

### *Intermediate 2 for Compound G*

*Di-tert-butyl (2S)-2-[[(1S)-1-tert-butoxycarbonyl-5-[[(2S)-2-[[5-[(9H-fluoren-9-ylmethoxycarbonylamino)methyl]pyridine-2-carbonyl]amino]-3-(2-naphthyl)propanoyl]amino]pentyl]carbamoylamino]pentanedioate*

Di-tert-butyl (2S)-2-({[(2S)-6-{[(2S)-2-amino-3-(naphthalen-2-yl)propanoyl]amino}-1-tert-butoxy-1-oxohexan-2-yl]carbamoyl}amino)pentanedioate (129 mg, 85% purity, 160 µmol) and 5-[({[(9H-fluoren-9-yl)methoxy]carbonyl}amino)methyl]pyridine-2-carboxylic acid (70.5 mg, 85% purity, 160 µmol) were solubilized in DMF (1.2 mL), 4-methylmorpholine (79 µL, 640 µmol, CAS-RN: 109-02-4) and HATU (102 mg, 240 µmol) were added, and the mixture was stirred under argon atmosphere at room temperature. The mixture was diluted with brine and extracted 3 times with DCM. The combined organic layers were dried, evaporated, and purified by preparative HPLC (C18, acetonitrile/water with 0.1% formic acid) to give 144 mg (90% purity, 78% yield) of the target compound. LC-MS (Method 1): R_t_ = 1.63 min; MS (ESIpos): m/z = 1042 [M+H]^+^. ^1^H-NMR (400MHz, DMSO-*d*_6_) δ ppm 8.64 (d, *J*=8.62 Hz, 1 H), 8.48 (d, *J*=1.27 Hz, 1 H), 8.21 (br t, *J*=5.32 Hz, 1 H), 7.86 - 7.97 (m, 4 H), 7.80 - 7.85 (m, 1 H), 7.72 - 7.79 (m, 2 H), 7.67 (d, *J*=8.11 Hz, 3 H), 7.34 - 7.48 (m, 5 H), 7.27 - 7.33 (m, 2 H), 6.27 (dd, *J*=14.07, 8.24 Hz, 2 H), 4.77 - 4.86 (m, 1 H), 4.37 (d, *J*=6.84 Hz, 2 H), 4.15 - 4.29 (m, 3 H), 4.03 (td, *J*=8.49, 5.32 Hz, 1 H), 3.90 - 3.98 (m, 1 H), 3.16 - 3.29 (m, 2 H), 2.95 - 3.14 (m, 2 H), 2.12 - 2.30 (m, 2 H), 1.80 - 1.91 (m, 1 H), 1.42 - 1.72 (m, 3 H), 1.31 - 1.40 (m, 29 H), 1.16 - 1.28 (m, 2 H).

### *Intermediate 3 for Compound G*

*Tri-tert-butyl (3S,10S,14S)-1-[5-(aminomethyl)pyridin-2-yl]-3-[(naphthalen-2-yl)methyl]-1,4,12-trioxo-2,5,11,13-tetraazahexadecane-10,14,16-tricarboxylate*

Tri-tert-butyl (3S,10S,14S)-1-{5-[({[(9H-fluoren-9-yl)methoxy]carbonyl}amino)methyl] pyridin-2-yl}-3-[(naphthalen-2-yl)methyl]-1,4,12-trioxo-2,5,11,13-tetraazahexadecane-10,14,16-tricarboxylate (142 mg, 90% purity, 123 µmol) was solubilized in DMF (1.9 mL), piperidine (105 mg, 1.23 mmol) was added, and the mixture was stirred under argon atmosphere at room temperature for 1 h. The mixture was evaporated and purified by preparative HPLC (C18, acetonitrile/water with 0.1% formic acid) to give 66.0 mg (95% purity, 62% yield) of the target compound. LC-MS (Method 1): R_t_ = 1.21 min; MS (ESIpos): m/z = 819 [M+H]^+^. ^1^H-NMR (400 MHz, DMSO-*d*_6_) δ ppm 8.64 - 8.69 (m, 2 H), 8.26 (br t, *J*=5.58 Hz, 1 H), 8.02 (s, 2 H), 7.80 - 7.86 (m, 1 H), 7.72 - 7.80 (m, 2 H), 7.68 (s, 1 H), 7.41 - 7.48 (m, 2 H), 7.36 (dd, *J*=8.49, 1.65 Hz, 1 H), 6.28 (dd, *J*=13.05, 8.49 Hz, 2 H), 4.78 - 4.88 (m, 1 H), 4.15 (s, 2 H), 4.03 (td, *J*=8.43, 5.20 Hz, 1 H), 3.90 - 3.98 (m, 1 H), 3.16 - 3.29 (m, 2 H), 3.05 - 3.14 (m, 1 H), 2.96 - 3.05 (m, 3 H), 2.11 - 2.30 (m, 2 H), 1.80 - 1.92 (m, 1 H), 1.60 - 1.71 (m, 3 H), 1.56 (m, 2 H), 1.43 - 1.50 (m, 1 H), 1.38 (m, 28 H), 1.18 - 1.30 (m, 2 H).

### *Intermediate 4 for Compound G*

*Tri-tert-butyl (3S,10S,14S)-3-[(naphthalen-2-yl)methyl]-1,4,12-trioxo-1-[5-({2-[4,7,10-tris(2-tert-butoxy-2-oxoethyl)-1,4,7,10-tetraazacyclododecan-1-yl]acetamido}methyl)pyridin-2-yl]-2,5,11,13-tetraazahexadecane-10,14,16-tricarboxylate*

N-[9,12-bis(2-tert-butoxy-2-oxoethyl)-2,2,6-trimethyl-4-oxo-3-oxa-6,9,12-triazatetradecan-14-yl]-N-ethylglycine (119 mg, 202 µmol), [(1H-benzotriazol-1-yl)oxy](dimethylamino)-N,N-dimethylmethaniminium hexafluoridophosphate(1-) (84.1 mg, 200 µmol; CAS-RN:94790-37-1) and N,N-diisopropylethylamine (29 µL, 150 µmol) were stirred in DMF (970 µL) at room temperature for 10 min. Tri-tert-butyl (3S,10S,14S)-1-[5-(aminomethyl)pyridin-2-yl]-3-[(naphthalen-2-yl)methyl]-1,4,12-trioxo-2,5,11,13-tetraazahexadecane-10,14,16-tricarboxylate (46.0 mg, 90% purity, 50.5 µmol) was added and the mixture was stirred at room temperature for 5 h. The mixture was evaporated and purified by preparative HPLC (C18, acetonitrile/water with 0.1% formic acid) to give 52.0 mg (80% purity, 60% yield) of the target compound. LC-MS (Method 1): R_t_ = 1.37 min; MS (ESIpos): m/z = 1374 [M+H]^+^.

### *Compound G*

*(3S,10S,14S)-3-[(naphthalen-2-yl)methyl]-1,4,12-trioxo-1-[5-({2-[4,7,10-tris(carboxymethyl)-1,4,7,10-tetraazacyclododecan-1-yl]acetamido}methyl)pyridin-2-yl]-2,5,11,13-tetraazahexadecane-10,14,16-tricarboxylic acid*

Tri-tert-butyl (3S,10S,14S)-3-[(naphthalen-2-yl)methyl]-1,4,12-trioxo-1-[5-({2-[4,7,10-tris(2-tert-butoxy-2-oxoethyl)-1,4,7,10-tetraazacyclododecan-1-yl]acetamido}methyl)pyridin-2-yl]-2,5,11,13-tetraazahexadecane-10,14,16-tricarboxylate (50.0 mg, 80% purity, 29.1 µmol) was solubilized in DCM (740 µL), TFA (670 µL, 8.7 mmol) was added, and the mixture was stirred under argon atmosphere at room temperature overnight. The mixture was evaporated and purified by preparative HPLC (C18, acetonitrile/water with 0.1% formic acid) to give 7.10 mg (95% purity, 22% yield) of the target compound. LC-MS (Method 1): Rt = 0.72 min; MS (ESIpos): m/z = 1038 [M+H]^+^. ^1^H-NMR (400 MHz, DMSO-*d*_6_) δ ppm 8.75 - 8.82 (m, 1 H), 8.70 (br d, *J*=8.36 Hz, 1 H), 8.56 - 8.65 (m, 1 H), 8.15 - 8.22 (m, 1 H), 7.91 - 8.00 (m, 1 H), 7.75 - 7.89 (m, 4 H), 7.70 (s, 1 H), 7.42 - 7.46 (m, 2 H), 7.38 (d, *J*=8.11 Hz, 1 H), 6.19 - 6.42 (m, 2 H), 4.74 - 4.83 (m, 1 H), 4.39 (br dd, *J*=4.31, 1.27 Hz, 2 H), 3.94 - 4.10 (m, 2 H), 3.41 - 3.49 (m, 6 H), 3.15 - 3.26 (m, 8 H), 2.84 - 3.12 (m, 16 H), 2.55 - 2.63 (m, 3 H), 2.23 (br t, *J*=7.48 Hz, 2 H), 1.79 - 1.88 (m, 1 H), 1.67 - 1.78 (m, 1 H), 1.52 - 1.65 (m, 1 H), 1.40 - 1.51 (m, 1 H), 1.30 - 1.38 (m, 2 H), 1.23 (br s, 3 H).

**Synthesis of Compound H**

### *Intermediate 1 for Compound H*

*Tri-tert-butyl (3S,10S,14S)-1-{5-[({[(9H-fluoren-9-yl)methoxy]carbonyl} amino)methyl]pyridin-2-yl}-1,4,12-trioxo-3-[(quinolin-2-yl)methyl]-2,5,11,13-tetraazahexadecane-10,14,16-tricarboxylate*

Di-tert-butyl(2S)-2-({[(2S)-6-{[(2S)-2-amino-3-(quinolin-2-yl)propanoyl]amino}-1-tert-butoxy-1-oxohexan-2-yl]carbamoyl}amino)pentanedioate (83.0 mg, 121 µmol) and (2*S*)-5-[({[(9H-fluoren-9-yl)methoxy]carbonyl}amino)methyl]pyridine-2-carboxylic acid (64.0 mg, 85% purity, 145 µmol) were solubilized in DMF (940 µL), 4-methylmorpholine (79 µL, 480 µmol, CAS-RN: 109-02-4) and HATU (102 mg, 182 µmol) were added, and the mixture was stirred under argon atmosphere at room temperature overnight. The mixture was evaporated and purified by preparative HPLC (C18, acetonitrile/water with 0.1% formic acid) to give 82.0 mg (65% yield) of the target compound. LC-MS (Method 1): Rt = 1.57 min; MS (ESIpos): m/z = 1043 [M+H]^+^. ^1^H-NMR (400 MHz, DMSO-*d*_6_) δ ppm 9.14 (br d, *J*=8.36 Hz, 1 H), 8.54 (d, *J*=1.27 Hz, 1 H), 8.23 (d, *J*=8.36 Hz, 1 H), 8.14 (br t, *J*=5.45 Hz, 1 H), 7.86 - 7.99 (m, 6 H), 7.65 - 7.77 (m, 4 H), 7.50 - 7.56 (m, 1 H), 7.40 (s, 3 H), 7.28 - 7.34 (m, 2 H), 6.27 (dd, *J*=17.74, 8.36 Hz, 2 H), 4.89 - 4.98 (m, 1 H), 4.37 (d, *J*=6.84 Hz, 2 H), 4.20 - 4.31 (m, 2 H), 4.03 (td, *J*=8.49, 5.32 Hz, 1 H), 3.87 - 3.96 (m, 1 H), 3.38 - 3.46 (m, 2 H), 2.93 - 3.09 (m, 2 H), 2.12 - 2.30 (m, 2 H), 1.80 - 1.92 (m, 1 H), 1.59 - 1.73 (m, 2 H), 1.48 - 1.58 (m, 2 H), 1.42 - 1.47 (m, 1 H), 1.27 - 1.41 (m, 28 H), 1.12 - 1.26 (m, 2 H).

### *Intermediate 2 for Compound H*

*Tri-tert-butyl (3S,10S,14S)-1-[5-(aminomethyl)pyridin-2-yl]-1,4,12-trioxo-3-[(quinolin-2-yl)methyl]-2,5,11,13-tetraazahexadecane-10,14,16-tricarboxylate*

Tri-tert-butyl(3S,10S,14S)-1-{5-[({[(9H-fluoren-9-yl)methoxy]carbonyl}amino)methyl] pyridin-2-yl}-1,4,12-trioxo-3-[(quinolin-2-yl)methyl]-2,5,11,13-tetraazahexadecane-10,14,16-tricarboxylate (80.0 mg, 76.8 µmol) was solubilized in DMF (1.2 mL), piperidine (150 µL, 1.5 mmol) was added, and the mixture was stirred under argon atmosphere at room temperature overnight. The mixture was evaporated and purified by preparative HPLC (C18, acetonitrile/water with 0.1% formic acid) to give 37.0 mg (97% purity, 57% yield) of the target compound. LC-MS (Method 1): Rt = 1.14 min; MS (ESIpos): m/z = 821 [M+H]^+^. ^1^H-NMR (400 MHz, DMSO-*d*_6_) δ ppm 9.13 (d, *J*=8.36 Hz, 1 H), 8.63 (s, 1 H), 8.21 - 8.27 (m, 1 H), 8.15 (br t, *J*=5.45 Hz, 1 H), 7.88 - 7.99 (m, 4 H), 7.73 (td, *J*=7.73, 1.27 Hz, 1 H), 7.51 - 7.57 (m, 1 H), 7.41 (d, *J*=8.62 Hz, 1 H), 6.27 (dd, *J*=17.49, 8.36 Hz, 2 H), 4.89 - 4.98 (m, 1 H), 4.03 (td, *J*=8.49, 5.07 Hz, 1 H), 3.87 - 3.95 (m, 1 H), 3.86 (s, 2 H), 3.40 (br d, *J*=6.34 Hz, 2 H), 2.95 - 3.08 (m, 2 H), 2.13 - 2.30 (m, 2 H), 1.80 - 1.91 (m, 1 H), 1.60 - 1.71 (m, 1 H), 1.47 - 1.58 (m, 1 H), 1.27 - 1.46 (m, 31 H), 1.14 - 1.25 (m, 3 H).

*Intermediate 3* *for Compound H*

*Tri-tert-butyl (3S,10S,14S)-1,4,12-trioxo-3-[(quinolin-2-yl)methyl]-1-[5-({2-[4,7,10-tris(2-tert-butoxy-2-oxoethyl)-1,4,7,10-tetraazacyclododecan-1-yl]acetamido}methyl)pyridin-2-yl]-2,5,11,13-tetraazahexadecane-10,14,16-tricarboxylate*

[4,7,10-tris(2-tert-butoxy-2-oxoethyl)-1,4,7,10-tetraazacyclododecan-1-yl]acetic acid (64.3 mg, 112 µmol and N,N-diisopropylethylamine (14 µl, 84 µmol) were stirred in DMF (1.1 mL) at room temperature for 10 min. [(1H-benzotriazol-1-yl)oxy](dimethylamino)-N,N-dimethylmethaniminium hexafluoridophosphate(1-) (42.0 mg, 111 µmol) was added portion wise and the stirring continued for further 10 min. Then tri-tert-butyl (3S,10S,14S)-1-[5-(aminomethyl)pyridin-2-yl]-3-[(naphthalen-2-yl)methyl]-1,4,12-trioxo-2,5,11,13-tetraazahexadecane-10,14,16-tricarboxylate (23.0 mg, 28.0 µmol) was added and the mixture was stirred at room temperature for 14 h. The mixture was evaporated and purified by preparative HPLC (C18, acetonitrile/water with 0.1% formic acid) to give 20.0 mg (80% purity, 42% yield) of the target compound. LC-MS (Method 1): R_t_ = 1.37 min; MS (ESIpos): m/z = 1374 [M+H]^+^.

### *Compound H*

*N^6^-{3-(quinolin-2-yl)-N-[5-({2-[4,7,10-tris(carboxymethyl)-1,4,7,10-tetraazacyclododecan-1-yl]acetamido}methyl)pyridine-2-carbonyl]-L-alanyl}-N^2^-{[(1S)-1,3-dicarboxypropyl]carbamoyl}-L-lysine*

Tri-tert-butyl (3S,10S,14S)-1,4,12-trioxo-3-[(quinolin-2-yl)methyl]-1-[5-({2-[4,7,10-tris(2-tert-butoxy-2-oxoethyl)-1,4,7,10-tetraazacyclododecan-1-yl]acetamido}methyl)pyridin-2-yl]-2,5,11,13-tetraazahexadecane-10,14,16-tricarboxylate (18.0 mg, 13.1 µmol) was solubilized in DCM (420 µL), TFA (200 µL, 2.6 mmol) was added and the mixture was stirred under argon overnight at room temperature. The mixture was evaporated and purified by preparative HPLC (C18, acetonitrile/water with 0.1% formic acid) to give 5.50 mg (95% purity, 38% yield) of the target compound. MS (ESIpos): m/z = 520 [M+2H]^2+^. ^1^H-NMR (400 MHz, DMSO-*d*_6_) δ ppm 9.17 (d, *J*=8.36 Hz, 1 H), 8.75 - 8.89 (m, 1 H), 8.66 (s, 1 H), 8.23 (s, 1 H), 8.07 - 8.18 (m, 1 H), 7.81 - 8.02 (m, 4 H), 7.74 (ddd, *J*=8.36, 6.97, 1.39 Hz, 1 H), 7.54 (td, *J*=7.54, 1.14 Hz, 1 H), 7.42 (d, *J*=8.36 Hz, 1 H), 6.15 - 6.42 (m, 2 H), 4.85 - 4.98 (m, 1 H), 4.40 (br d, *J*=5.07 Hz, 2 H), 4.03 - 4.13 (m, 1 H), 3.92 - 4.02 (m, 1 H), 3.39 - 3.49 (m, 9 H), 2.83 - 3.14 (m, 16 H), 2.55 - 2.64 (m, 3 H), 2.19 - 2.28 (m, 2 H), 1.65 - 1.78 (m, 2 H), 1.50 - 1.63 (m, 1 H), 1.41 - 1.50 (m, 1 H), 1.38 (d, *J*=1.27 Hz, 3 H), 1.14 - 1.34 (m, 7 H).

**Synthesis of PSMA-617**

### *Intermediate 1 for PSMA-617*

*Di-tert-butyl N-{[(2S)-6-{[(benzyloxy)carbonyl]amino}-1-tert-butoxy-1-oxohexan-2-yl]carbamoyl}-L-glutamate*

Tert-butyl N6-[(benzyloxy)carbonyl]-L-lysinate—hydrogen chloride (1/1) (7.27 g, 19.5 mmol; CAS-RN:[5978-22-3]) was solubilized in DCM (100 mL), cooled to 0 °C under argon atmosphere, and N,N-diisopropylethylamine (14 ml, 78 mmol) was added dropwise. The mixture was stirred at 0 °C for 5 min and at room temperature for 30 min. 4-nitrophenyl carbonochloridate (3.59 g, 17.8 mmol; CAS-RN:[7693-46-1]) and di-tert-butyl L-glutamate—hydrogen chloride (1/1) (5.00 g, 16.9 mmol; CAS-RN:[32677-01-3]) were added and N,N-diisopropylethylamine (14 mL, 78 mmol) was added dropwise to the mixture. It was stirred at room temperature overnight. The mixture was concentrated under reduced pressure, diluted with DCM, and washed 2 times with sodium hydroxide (0.5 M) and once with brine. The organic layer was dried and evaporated. The residue was purified by flash chromatography (SiO_2_, hexane/EtOAc gradient 0-50%) to give 8.40 g (96% purity, 67% yield) of the target compound. LC-MS (Method 1): Rt = 1.48 min; MS (ESIpos): m/z = 623 [M+H]^+^. ^1^H-NMR (400 MHz, DMSO-*d*_6_) δ ppm 1.21 - 1.31 (m, 2 H) 1.34 - 1.44 (m, 27 H) 1.45 - 1.75 (m, 2 H) 1.79 - 1.92 (m, 1 H) 2.13 - 2.29 (m, 2 H) 2.92 - 3.02 (m, 2 H) 3.33 (s, 2 H) 3.91 - 3.99 (m, 1 H) 3.99 - 4.08 (m, 2 H) 4.99 (s, 2 H) 6.17 - 6.37 (m, 2 H) 7.19 - 7.27 (m, 1 H) 7.27 - 7.41 (m, 5 H).

### *Intermediate 2 for PSMA-617*

*Di-tert-butyl N-{[(2S)-6-amino-1-tert-butoxy-1-oxohexan-2-yl]carbamoyl}-L-glutamate*

Di-tert-butyl N-{[(2S)-6-{[(benzyloxy)carbonyl]amino}-1-tert-butoxy-1-oxohexan-2-yl]carbamoyl}-L-glutamate (8.48 g, 13.6 mmol) was solubilized in MeOH (42 mL), palladium on carbon (1.45 g, 10% purity, 1.36 mmol) was added, and the mixture was purged with hydrogen. The mixture was stirred under hydrogen atmosphere at room temperature for 4 h. The mixture was filtered over Celite, washed with MeOH, and evaporated. The residue was diluted with DCM and washed 3 times with saturated sodium hydrogen carbonate solution and once with brine. The organic layer was dried and evaporated. The residue was purified by flash chromatography (SiO_2_, DCM/Ethanol gradient 0-20%) to give 4.06 g (97% purity, 59% yield) of the target compound. LC-MS (Method 1): Rt = 0.97 min; MS (ESIpos): m/z = 489 [M+H]^+^. ^1^H-NMR (400 MHz, DMSO-*d*_6_) δ ppm 1.23 - 1.34 (m, 3 H) 1.37 - 1.42 (m, 27 H) 1.45 - 1.71 (m, 3 H) 1.78 - 1.93 (m, 1 H) 2.10 - 2.30 (m, 2 H) 3.16 (s, 2 H) 3.33 (br s, 2 H) 3.91 - 4.06 (m, 2 H) 4.07 - 4.16 (m, 1 H) 6.27 (dd, *J*=17.11, 8.49 Hz, 2 H).

### *Intermediate 3 for PSMA-617*

*Tri-tert-butyl (5S,12S,16S)-1-(9H-fluoren-9-yl)-5-[(naphthalen-2-yl)methyl]-3,6,14-trioxo-2-oxa-4,7,13,15-tetraazaoctadecane-12,16,18-tricarboxylate*

Di-tert-butyl N-{[(2S)-6-amino-1-tert-butoxy-1-oxohexan-2-yl]carbamoyl}-L-glutamate (1.00 g, 70% purity, 1.44 mmol) and (2S)-2-({[(9H-fluoren-9-yl)methoxy]carbonyl}amino)-3-(naphthalen-2-yl)propanoic acid (940 mg, 2.15 mmol; CAS-RN:[112883-43-9]) were solubilized in DMF (11 mL), 4-methylmorpholine (470 µL, 4.3 mmol, CAS-RN: 109-02-4) and HATU (819 mg, 2.15 mmol) were added, and the mixture was stirred under argon atmosphere at room temperature overnight. The mixture was evaporated and purified by preparative HPLC (C18, acetonitrile/water with 0.1% formic acid) to give 1.11 g (93% purity, 79% yield) of the target compound. LC-MS (Method 1): R_t_ = 1.67 min; MS (ESIpos): m/z = 908 [M+H]^+^. ^1^H-NMR (400 MHz, DMSO-*d*_6_) δ ppm 8.01 (s, 1 H) 7.75 - 7.88 (m, 6 H) 7.67 (d, *J*=8.62 Hz, 1 H) 7.60 (dd, *J*=13.05, 7.48 Hz, 2 H) 7.42 - 7.50 (m, 3 H) 7.37 (q, *J*=7.44 Hz, 2 H) 7.25 (t, *J*=7.10 Hz, 1 H) 7.16 (t, *J*=6.99 Hz, 1 H) 6.27 (dd, *J*=14.19, 8.36 Hz, 2 H) 4.29 (br s, 1 H) 4.00 - 4.14 (m, 4 H) 3.94 (br d, *J*=5.58 Hz, 1 H) 3.04 - 3.15 (m, 2 H) 2.91 - 3.03 (m, 2 H) 2.52 - 2.53 (m, 1 H) 2.14 - 2.28 (m, 2 H) 1.66 (s, 1 H) 1.54 (br s, 1 H) 1.46 (s, 1 H) 1.38 (s, 27 H) 1.32 - 1.36 (m, 2 H) 1.23 (br d, *J*=6.59 Hz, 2 H).

### *Intermediate 4 for PSMA-617*

*Di-tert-butyl (2S)-2-({[(2S)-6-{[(2S)-2-amino-3-(naphthalen-2-yl)propanoyl]amino}-1-tert-butoxy-1-oxohexan-2-yl]carbamoyl}amino)pentanedioate*

Tri-tert-butyl (5S,12S,16S)-1-(9H-fluoren-9-yl)-5-[(naphthalen-1-yl)methyl]-3,6,14-trioxo-2-oxa-4,7,13,15-tetraazaoctadecane-12,16,18-tricarboxylate (1.11 g, 93% purity, 1.14 mmol) was solubilized in DMF (26 ml), piperidine (2.3 mL, 23 mmol) was added, and the mixture was stirred under argon atmosphere at room temperature overnight. The mixture was evaporated and purified by preparative HPLC (C18, acetonitrile/water with 0.1% formic acid) to give 663 mg (85% yield) of the target compound. LC-MS (Method 1): R_t_ = 1.22 min; MS (ESIpos): m/z = 686 [M+H]^+^. ^1^H-NMR (400 MHz, DMSO-*d*_6_) δ ppm 7.78 - 7.90 (m, 4 H) 7.68 (s, 1 H) 7.42 - 7.50 (m, 2 H) 7.38 (d, *J*=8.64 Hz, 1 H) 7.22 - 7.27 (m, 1 H) 7.14 - 7.19 (m, 1 H) 6.21 - 6.33 (m, 2 H) 4.03 (td, *J*=8.55, 5.20 Hz, 1 H) 3.92 (td, *J*=8.05, 5.45 Hz, 1 H) 3.40 - 3.56 (m, 2 H) 2.96 - 3.11 (m, 3 H) 2.72 - 2.89 (m, 1 H) 2.52 - 2.54 (m, 1 H) 2.28 - 2.32 (m, 1 H) 2.18 - 2.26 (m, 2 H) 1.81 - 1.90 (m, 1 H) 1.61 - 1.72 (m, 1 H) 1.48 - 1.58 (m, 1 H) 1.38 (m, 29 H) 1.16 - 1.24 (m, 2 H).

### *Intermediate 5 for PSMA-617*

*Tri-tert-butyl (3S,10S,14S)-1-{(1r,4S)-4-[({[(9H-fluoren-9-yl)methoxy]carbonyl}amino) methyl]cyclohexyl}-3-[(naphthalen-2-yl)methyl]-1,4,12-trioxo-2,5,11,13-tetraazahexadecane-10,14,16-tricarboxylate*

Di-tert-butyl (2S)-2-({[(2S)-6-{[(2S)-2-amino-3-(naphthalen-2-yl)propanoyl]amino}-1-tert-butoxy-1-oxohexan-2-yl]carbamoyl}amino)pentanedioate (116 mg, 70% purity, 119 µmol) and (1R,4R)-4-[({[(9H-fluoren-9-yl)methoxy]carbonyl}amino)methyl]cyclo-hexane-1-carboxylic acid (67.5 mg, 178 µmol) were solubilized in DMF (910 µL), 4-methylmorpholine (39 µL, 360 µmol, CAS-RN: 109-02-4) and HATU (67.6 mg, 178 µmol) were added, and the mixture was stirred under argon atmosphere at room temperature overnight. The mixture was evaporated and purified by preparative HPLC (C18, acetonitrile/water with 0.1% formic acid) to give 134 mg (80% purity, 86% yield) of the target compound. LC-MS (Method 1): R_t_ = 1.66 min; MS (ESIpos): m/z = 1047 [M+H]^+^. ^1^H-NMR (400 MHz, DMSO-*d*_6_) δ ppm 7.76 - 8.01 (m, 7 H) 7.68 (br d, *J*=6.59 Hz, 3 H) 7.21 - 7.50 (m, 8 H) 6.18 - 6.36 (m, 2 H) 4.47 - 4.60 (m, 1 H) 4.25 - 4.35 (m, 2 H) 4.14 - 4.23 (m, 1 H) 3.99 - 4.08 (m, 1 H) 3.87 - 3.97 (m, 1 H) 2.88 - 3.16 (m, 4 H) 2.73 - 2.82 (m, 2 H) 2.14 - 2.28 (m, 2 H) 2.01 - 2.11 (m, 1 H) 1.81 - 1.94 (m, 1 H) 1.38 (d, *J*=2.79 Hz, 41 H) 0.95 - 1.09 (m, 1 H) 0.74 - 0.82 (m, 1 H).

### *Intermediate 6 for PSMA-617*

*Tri-tert-butyl (3S,10S,14S)-1-[(1r,4S)-4-(aminomethyl)cyclohexyl]-3-[(naphthalen-2-yl)methyl]-1,4,12-trioxo-2,5,11,13-tetraazahexadecane-10,14,16-tricarboxylate*

Tri-tert-butyl (3S,10S,14S)-1-{(1R,4S)-4-[({[(9H-fluoren-9-yl)methoxy]carbonyl}amino) methyl]cyclohexyl}-3-[(naphthalen-2-yl)methyl]-1,4,12-trioxo-2,5,11,13-tetraazahexa-decane-10,14,16-tricarboxylate (730 mg, 98% purity, 684 µmol) was solubilized in DMF (18 mL), piperidine (1.4 mL, 14 mmol) was added, and the mixture was stirred under argon atmosphere at room temperature overnight. The mixture was evaporated and purified by preparative HPLC (C18, acetonitrile/water with 0.1% formic acid) to give 381 mg (98% purity, 66% yield) of the target compound. LC-MS (Method 1): R_t_ = 1.20 min; MS (ESIpos): m/z = 825 [M+H]^+^. ^1^H-NMR (400 MHz, DMSO-*d*_6_) δ ppm 8.36 - 8.45 (m, 1 H) 7.95 - 8.03 (m, 2 H) 7.75 - 7.88 (m, 3 H) 7.64 - 7.71 (m, 1 H) 7.36 - 7.50 (m, 3 H) 6.18 - 6.42 (m, 2 H) 4.47 - 4.59 (m, 1 H) 3.99 - 4.08 (m, 1 H) 3.87 - 3.97 (m, 1 H) 2.86 - 3.13 (m, 4 H) 2.52 (br s, 3 H) 2.13 - 2.29 (m, 2 H) 2.02 - 2.12 (m, 1 H) 1.81 - 1.92 (m, 1 H) 1.60 - 1.77 (m, 4 H) 1.13 - 1.58 (m, 35 H) 0.96 - 1.11 (m, 1 H) 0.75 - 0.91 (m, 2 H).

### *Intermediate 7 for PSMA-617*

*(3S,10S,14S)-1-[(1r,4S)-4-(aminomethyl)cyclohexyl]-3-[(naphthalen-2-yl)methyl]-1,4,12-trioxo-2,5,11,13-tetraazahexadecane-10,14,16-tricarboxylic acid*

Tri-tert-butyl (3S,10S,14S)-1-[(1r,4S)-4-(aminomethyl)cyclohexyl]-3-[(naphthalen-2-yl)methyl]-1,4,12-trioxo-2,5,11,13-tetraazahexadecane-10,14,16-tricarboxylate (25.0 mg, 98% purity, 29.7 µmol) was solubilized in DCM (1.0 mL), TFA (1.0 mL, 13 mmol) was added, and the mixture was stirred under argon atmosphere at room temperature for 2 h. The mixture was evaporated and purified by preparative HPLC (C18, acetonitrile/water with 0.1% formic acid) to give 12.0 mg (95% purity, 58% yield) of the target compound. LC-MS (Method 1): R_t_ = 0.70 min; MS (ESIpos): m/z = 657 [M+H]^+^. ^1^H-NMR (400 MHz, DMSO-*d*_6_) δ ppm 8.06 - 8.32 (m, 1 H) 7.65 - 8.02 (m, 5 H) 7.30 - 7.55 (m, 3 H) 6.33 - 6.52 (m, 1 H) 5.95 - 6.28 (m, 1 H) 4.43 - 4.64 (m, 1 H) 3.87 - 4.03 (m, 2 H) 2.87 - 3.15 (m, 5 H) 2.55 - 2.71 (m, 4 H) 2.07 - 2.34 (m, 4 H) 0.67 - 1.87 (m, 18 H).

### *Intermediate 8 for PSMA-617*

*Tri-tert-butyl (3S,10S,14S)-3-[(naphthalen-2-yl)methyl]-1,4,12-trioxo-1-[(1r,4S)-4-({2-[4,7,10-tris(2-tert-butoxy-2-oxoethyl)-1,4,7,10-tetraazacyclododecan-1-yl]acetamido}methyl)cyclohexyl]-2,5,11,13-tetraazahexadecane-10,14,16-tricarboxylate*

[4,7,10-tris(2-tert-butoxy-2-oxoethyl)-1,4,7,10-tetraazacyclododecan-1-yl]acetic acid (289 mg, 505 µmol), [(1H-benzotriazol-1-yl)oxy](dimethylamino)-N,N-dimethylmethaniminium hexafluoridophosphate(1-) (189 mg, 498 µmol; CAS-RN:94790-37-1), and N,N-diisopropylethylamine (88 µL, 500 µmol) were stirred in DMF (3.5 mL) at room temperature for 15min. Tri-tert-butyl (3S,10S,14S)-1-[(1r,4S)-4-(aminomethyl)cyclohexyl]-3-[(naphthalen-2-yl)methyl]-1,4,12-trioxo-2,5,11,13-tetraazahexadecane-10,14,16-tricarboxylate (104 mg, 126 µmol) was added and the mixture was stirred at room temperature. The mixture was evaporated and purified by preparative HPLC (C18, acetonitrile/water with 0.1% formic acid) to give 97.0 mg (99% purity, 55% yield) of the target compound. LC-MS (Method 1): R_t_ = 1.42 min; MS (ESIpos): m/z = 1379 [M+H]^+^.

### *PSMA-617*

*(3S,10S,14S)-3-[(naphthalen-2-yl)methyl]-1,4,12-trioxo-1-[(1r,4S)-4-({2-[4,7,10-tris(carboxymethyl)-1,4,7,10-tetraazacyclododecan-1-yl]acetamido}methyl)cyclohexyl]-2,5,11,13-tetraazahexadecane-10,14,16-tricarboxylic acid*

Tri-tert-butyl (3S,10S,14S)-3-[(naphthalen-2-yl)methyl]-1,4,12-trioxo-1-[(1r,4S)-4-({2-[4,7,10-tris(2-tert-butoxy-2-oxoethyl)-1,4,7,10-tetraazacyclododecan-1-yl]acetamido}methyl)cyclohexyl]-2,5,11,13-tetraazahexadecane-10,14,16-tricarboxylate (96.0 mg, 98% purity, 68.2 µmol) was solubilized in DCM (9.6 mL), TFA (79 µL, 1.0 mmol) was added, and the mixture was stirred under argon atmosphere at room temperature. The mixture was evaporated and purified by preparative HPLC (C18, acetonitrile/water with 0.1% formic acid) to give 48.0 mg (95% purity, 64% yield) of the target compound. LC-MS (Method 1): R_t_ = 0.72 min; MS (ESIneg): m/z = 1041 [M-H]^-^. ^1^H-NMR (400 MHz, DMSO-*d*_6_) δ ppm 7.66 - 8.12 (m, 6 H) 7.33 - 7.53 (m, 3 H) 6.31 (br d, *J*=8.36 Hz, 2 H) 4.40 - 4.55 (m, 1 H) 3.93 - 4.15 (m, 2 H) 3.43 (br s, 12 H) 2.83 - 3.14 (m, 18 H) 2.57 - 2.67 (m, 3 H) 2.17 - 2.29 (m, 2 H) 1.99 - 2.13 (m, 1 H) 1.78 - 1.93 (m, 1 H) 1.02 - 1.75 (m, 12 H) 0.70 - 0.91 (m, 2 H).

### *^232^Th-PSMA-617*

*{3-[(naphthalen-2-yl)methyl]-1,4,12-trioxo-1-{4-[(2-{4,7,10-tris[(carboxy-kappaO)methyl]-1,4,7,10-tetraazacyclododecan-1-yl-kappa^4^N^1^,N^4^,N^7^,N^10^} acetamido)methyl]cyclohexyl}-2,5,11,13-tetraazahexadecane-10,14,16-tricarboxylato(4-)}thorium(3+)*

(3S,10S,14S)-3-[(naphthalen-2-yl)methyl]-1,4,12-trioxo-1-[(1r,4S)-4-({2-[4,7,10-tris(carboxymethyl)-1,4,7,10-tetraazacyclododecan-1-yl]acetamido}methyl)cyclohexyl]-2,5,11,13-tetraazahexadecane-10,14,16-tricarboxylic acid (28.6 mg, 27.4 µmol) was solubilized in ammonium acetate (29 mL, 1.0 M, 29 mmol, prepared with ultrafiltered and autoclaved water), thorium solution (6.6 mL, 27 µmol, 1 µg/µL in HNO_3_ [2%]) was added, and the mixture was stirred at 90 °C for 3.5 h. The mixture was evaporated and diluted with DMF. The resulting suspension was filtered, washed with DMF, and the filtrate was purified by preparative HPLC (C18, acetonitrile/water with 0.1% formic acid) to give 1.20 mg (75% purity, 3% yield) of the target compound. LC-MS (Method 1): R_t_ = 0.74 min; MS (ESIpos): m/z = 1271 [M+H]^+^.

### *Final ^227^Th-PSMA-617*

*[^227^Th]Thorium (3S,10S,14S)-3-[(naphthalen-2-yl)methyl]-1,4,12-trioxo-1-{(1r,4S)-4-[(2-{4,7,10-tris[(carboxy-kappaO)methyl]-1,4,7,10-tetraazacyclododecan-1-yl-kappa^4^N^1^,N^4^,N^7^,N^10^}acetamido)methyl]cyclohexyl}-2,5,11,13-tetraazahexadecane-10,14,16-tricarboxylate*

(3S,10S,14S)-3-[(naphthalen-2-yl)methyl]-1,4,12-trioxo-1-[(1r,4S)-4-({2-[4,7,10-tris(carboxymethyl)-1,4,7,10-tetraazacyclododecan-1-yl]acetamido}methyl)cyclohexyl]-2,5,11,13-tetraazahexadecane-10,14,16-tricarboxylic acid dissolved in 400 mM sodium acetate buffer (pH 5.6) containing 0.5 mg/mL pABA was mixed with thorium-227 in 0.5 M HCl at 0.3 MBq/nmol specific activity and RAC of 7.2 MBq/mL at 95 °C for 40 min. The labelling efficiency was determined to be 92% by iTLC.

**Synthesis of *D*-Glu-PSMA-617**

*Intermediate 1 for D-Glu-PSMA-617*

*Di-tert-butyl N-{[(2S)-6-{[(benzyloxy)carbonyl]amino}-1-tert-butoxy-1-oxohexan-2-yl]carbamoyl}-D-glutamate*

Di-tert-butyl D-glutamate hydrogen chloride salt (4.97 g, 16.8 mmol) and 4-nitrophenyl carbonochloridate (3.57 g, 17.7 mmol) were solubilized in DCM (51 mL), cooled to 0 °C under argon atmosphere, and N,N-diisopropylethylamine (6.7 mL, 39 mmol) was added dropwise. The mixture was stirred at 0 °C for 5 min and at room temperature for 30 min. Tert-butyl *N*6-[(benzyloxy)carbonyl]-L-lysinate / hydrogen chloride (1/1) (7.22 g, 19.4 mmol) was added, followed by the dropwise addition of N,N-diisopropylethylamine (6.7 mL, 39 mmol) and the reaction mixture was stirred at room temperature for 1 h. The mixture was washed 3 times with saturated sodium hydrogen carbonate, once with sodium hydroxide (1.0 M), and once with brine. The organic layer was dried and concentrated under reduced pressure to give 13.3 g (78% purity, 99% yield) of the target compound, which was used without further purification. LC-MS (Method 1): R_t_ = 1.47 min; MS (ESIpos): m/z = 623 [M+H]^+^. ^1^H-NMR (400 MHz, DMSO-*d*_6_) δ ppm 7.19 - 7.41 (m, 6 H) 6.32 (dd, *J*=10.39, 8.36 Hz, 2 H) 4.99 (s, 2 H) 3.86 - 4.12 (m, 2 H) 2.97 (br dd, *J*=6.46, 1.39 Hz, 2 H) 2.11 - 2.29 (m, 2 H) 1.77 - 1.91 (m, 1 H) 1.45 - 1.75 (m, 3 H) 1.39 (d, *J*=5.07 Hz, 29 H) 1.13 - 1.28 (m, 2 H).

*Intermediate 2 for D-Glu-PSMA-617*

*Di-tert-butyl N-{[(2S)-6-amino-1-tert-butoxy-1-oxohexan-2-yl] carbamoyl}-D-glutamate*

Di-tert-butyl *N*-{[(2S)-6-{[(benzyloxy)carbonyl]amino}-1-tert-butoxy-1-oxohexan-2-yl] carbamoyl}-*D*-glutamate (2.37 g, 70% purity, 2.67 mmol) was solubilized in MeOH (40 mL). Palladium on carbon (60.0 mg, 10% purity) was added and the mixture was purged with hydrogen. The mixture was stirred at room temperature for 4.5 h under hydrogen atmosphere. The mixture was filtered over Celite, washed with MeOH, and concentrated under reduced pressure to give 1.80 g (70% purity, 97% yield) of the target compound, which was used without further purification. LC-MS (Method 1): R_t_ = 0.98 min; MS (ESIpos): m/z = 489 [M+H]^+^. ^1^H-NMR (400 MHz, DMSO-*d*_6_) δ ppm 6.28 (dd, *J*=17.24, 8.36 Hz, 2 H) 3.90 - 4.08 (m, 2 H) 2.11 - 2.29 (m, 2 H) 1.44 - 1.92 (m, 7 H) 1.37 - 1.42 (m, 28 H) 1.22 - 1.35 (m, 4 H).

*Intermediate 3 for D-Glu-PSMA-617*

*Tri-tert-butyl (5S,12S,16R)-1-(9H-fluoren-9-yl)-5-[(naphthalen-2-yl)methyl]-3,6,14-trioxo-2-oxa-4,7,13,15-tetraazaoctadecane-12,16,18-tricarboxylate*

Di-tert-butyl N-{[(2S)-6-amino-1-tert-butoxy-1-oxohexan-2-yl]carbamoyl}-D-glutamate (4.00 g, 85% purity, 6.97 mmol) and (2S)-2-({[(9H-fluoren-9-yl)methoxy] carbonyl}amino)-3-(naphthalen-2-yl)propanoic acid (4.58 g, 10.5 mmol; CAS-RN:[112883-43-9]) were solubilized in DMF (54 mL). 4-methylmorpholine (2.3 mL, 21 mmol, CAS-RN: 109-02-4) and HATU (3.98 g, 10.5 mmol) were added, and the mixture was stirred under argon atmosphere at room temperature overnight. The mixture was evaporated, diluted with water, and extracted 3 times with DCM. The combined organic layers were washed with brine, dried, and evaporated. The residue was purified by flash chromatography (SiO2, hexane/EtOAc gradient 0%-25%) to give 6.07 g (96% yield) of the target compound. LC-MS (Method 1): R_t_ = 1.65 min; MS (ESIpos): m/z = 908 [M+H]^+^. ^1^H-NMR (400 MHz, DMSO-*d*_6_) δ ppm 7.73 - 7.92 (m, 5 H) 7.55 - 7.71 (m, 3 H) 7.31 - 7.51 (m, 4 H) 7.10 - 7.30 (m, 4 H) 6.31 (t, *J*=8.62 Hz, 2 H) 4.29 (td, *J*=9.31, 4.94 Hz, 1 H) 3.90 - 4.17 (m, 5 H) 3.05 - 3.14 (m, 2 H) 2.91 - 3.03 (m, 2 H) 2.30 (s, 2 H) 2.10 - 2.26 (m, 2 H) 1.78 - 1.89 (m, 1 H) 1.61 - 1.73 (m, 1 H) 1.51 - 1.59 (m, 1 H) 1.28 - 1.48 (m, 29 H) 1.15 - 1.27 (m, 2 H).

*Intermediate 4 for D-Glu-PSMA-617*

*Di-tert-butyl (2R)-2-({[(2S)-6-{[(2S)-2-amino-3-(naphthalen-2-yl) propanoyl] amino}-1-tert-butoxy-1-oxohexan-2-yl]carbamoyl}amino)pentanedioate*

Tri-tert-butyl (5S,12S,16R)-1-(9H-fluoren-9-yl)-5-[(naphthalen-2-yl)methyl]-3,6,14-trioxo-2-oxa-4,7,13,15-tetraazaoctadecane-12,16,18-tricarboxylate (940 mg, 95% purity, 984 µmol) was solubilized in DMF (26 mL). Piperidine (1.9 mL, 20 mmol) was added, and the mixture was stirred under argon atmosphere at room temperature over the weekend. The mixture was evaporated and purified by preparative HPLC (C18, acetonitrile/water with 0.2% ammonia) to give 305 mg (98% purity, 44% yield) of the target compound. LC-MS (Method 1): R_t_ = 1.21 min; MS (ESIpos): m/z = 686 [M+H]^+^. ^1^H-NMR (400 MHz, DMSO-*d*_6_) δ ppm 7.77 - 7.91 (m, 4 H) 7.68 (s, 1 H) 7.32 - 7.50 (m, 3 H) 6.31 (dd, *J*=15.21, 8.11 Hz, 2 H) 4.19 (s, 1 H) 3.88 - 4.11 (m, 2 H) 3.45 (dd, *J*=7.98, 5.20 Hz, 1 H) 2.91 - 3.13 (m, 3 H) 2.78 (dd, *J*=13.18, 8.11 Hz, 1 H) 2.11 - 2.29 (m, 2 H) 1.62 - 1.90 (m, 3 H) 1.49 - 1.56 (m, 1 H) 1.26 - 1.47 (m, 29 H) 1.13 - 1.24 (m, 2 H).

*Intermediate 5 for D-Glu-PSMA-617*

*Tri-tert-butyl (3S,10S,14R)-1-{(1r,4S)-4-[({[(9H-fluoren-9-yl)methoxy]carbonyl}amino) methyl]cyclohexyl}-3-[(naphthalen-2-yl)methyl]-1,4,12-trioxo-2,5,11,13-tetraazahexadecane-10,14,16-tricarboxylate*

Di-tert-butyl (2R)-2-({[(2S)-6-{[(2S)-2-amino-3-(naphthalen-2-yl)propanoyl]amino}-1-tert-butoxy-1-oxohexan-2-yl]carbamoyl}amino)pentanedioate (305 mg, 445 µmol) and (1r,4r)-4-[({[(9H-fluoren-9-yl)methoxy]carbonyl}amino)methyl]cyclohexane-1-carboxylic acid (253 mg, 668 µmol) were solubilized in DMF (3.4 mL), 4-methylmorpholine (150 µL, 1.3 mmol, CAS-RN: 109-02-4) and HATU (254 mg, 668 µmol) were added, and the mixture was stirred under argon atmosphere at room temperature overnight. The mixture was purified by preparative HPLC (C18, acetonitrile/water with 0.1% formic acid) to give 160 mg (96% purity, 33% yield) of the target compound. LC-MS (Method 1): R_t_ = 1.65 min; MS (ESIpos): m/z = 1047 [M+H]^+^. ^1^H-NMR (400 MHz, DMSO-*d*_6_) δ ppm 7.74 - 8.00 (m, 7 H) 7.68 (br d, *J*=6.84 Hz, 3 H) 7.07 - 7.48 (m, 9 H) 6.30 (dd, *J*=14.95, 8.36 Hz, 2 H) 4.46 - 4.60 (m, 1 H) 4.29 (d, *J*=6.84 Hz, 2 H) 3.85 - 4.10 (m, 2 H) 2.86 - 3.16 (m, 4 H) 2.78 (br t, *J*=6.08 Hz, 2 H) 2.11 - 2.26 (m, 2 H) 1.76 - 1.91 (m, 1 H) 1.11 - 1.72 (m, 42 H) 0.70 - 0.91 (m, 2 H).

*Intermediate 6 for D-Glu-PSMA-617*

*Tri-tert-butyl (3S,10S,14R)-1-[(1r,4S)-4-(aminomethyl)cyclohexyl]-3-[(naphthalen-2-yl)methyl]-1,4,12-trioxo-2,5,11,13-tetraazahexadecane-10,14,16-tricarboxylate*

Tri-tert-butyl (3S,10S,14R)-1-{(1r,4S)-4-[({[(9H-fluoren-9-yl)methoxy]carbonyl}amino) methyl] cyclohexyl}-3-[(naphthalen-2-yl)methyl]-1,4,12-trioxo-2,5,11,13-tetraazahexa-decane-10,14,16-tricarboxylate (160 mg, 96% purity, 147 µmol) was solubilized in DMF (3.9 mL), piperidine (290 µl, 2.9 mmol) was added, and the mixture was stirred under argon atmosphere at room temperature. The mixture was evaporated and purified by preparative HPLC (C18, acetonitrile/water with 0.1% formic acid) to give 95.0 mg (100% purity, 79% yield) of the target compound. LC-MS (Method 1): R_t_ = 1.18 min; MS (ESIpos): m/z = 825 [M+H]^+^. ^1^H-NMR (400 MHz, DMSO-*d*_6_) δ ppm 8.41 (s, 1 H) 7.91 - 8.05 (m, 2 H) 7.75 - 7.89 (m, 3 H) 7.68 (s, 1 H) 7.30 - 7.55 (m, 3 H) 6.32 (dd, *J*=15.97, 8.11 Hz, 2 H) 4.53 (td, *J*=8.93, 5.20 Hz, 1 H) 3.88 - 4.13 (m, 2 H) 2.90 - 3.14 (m, 4 H) 2.10 - 2.28 (m, 3 H) 1.59 - 1.90 (m, 5 H) 1.13 - 1.57 (m, 39 H) 0.72 - 1.11 (m, 3 H).

*Intermediate 7 for D-Glu-PSMA-617*

*Tri-tert-butyl (3S,10S,14R)-3-[(naphthalen-2-yl)methyl]-1,4,12-trioxo-1-[(1r,4S)-4-({2-[4,7,10-tris(2-tert-butoxy-2-oxoethyl)-1,4,7,10-tetraazacyclododecan-1-yl]acetamido}methyl)cyclohexyl]-2,5,11,13-tetraazahexadecane-10,14,16-tricarboxylate*

[4,7,10-tris(2-tert-butoxy-2-oxoethyl)-1,4,7,10-tetraazacyclododecan-1-yl]acetic acid (448 mg, 781 µmol), [(1H-benzotriazol-1-yl)oxy](dimethylamino)-N,N-dimethylmethaniminium hexafluoridophosphate(1-) (293 mg, 772 µmol; CAS-RN:94790-37-1), and N,N-diisopropylethylamine (140 µl, 780 µmol) were stirred in DMF (5.4 mL) at room temperature for 15 min. Tri-tert-butyl (3S,10S,14S)-1-[(1r,4S)-4-(aminomethyl)cyclohexyl]-3-[(naphthalen-2-yl)methyl]-1,4,12-trioxo-2,5,11,13-tetraazahexadecane-10,14,16-tricarboxylate (161 mg, 195 µmol) was added and the mixture was stirred at room temperature. The mixture was evaporated and purified by preparative HPLC (C18, acetonitrile/water with 0.1% formic acid) to give 113 mg (97% purity, 41% yield) of the target compound. LC-MS (Method 1): R_t_ = 1.36 min; MS (ESIpos): m/z = 1380 [M+H]^+^. ^1^H-NMR (400 MHz, DMSO-*d*_6_) δ ppm 7.88 - 8.02 (m, 2 H) 7.84 (br d, *J*=7.10 Hz, 1 H) 7.73 - 7.81 (m, 2 H) 7.67 (s, 1 H) 7.45 (s, 4 H) 6.30 (dd, *J*=17.24, 8.36 Hz, 2 H) 4.48 - 4.59 (m, 1 H) 4.07 - 4.24 (m, 1 H) 4.02 (br d, *J*=5.58 Hz, 3 H) 3.83 - 3.89 (m, 1 H) 3.68 - 3.74 (m, 1 H) 3.49 - 3.56 (m, 3 H) 2.74 - 3.19 (m, 17 H) 2.54 (s, 3 H) 2.01 - 2.27 (m, 3 H) 1.78 - 1.90 (m, 1 H) 1.58 - 1.74 (m, 4 H) 1.12 - 1.56 (m, 66 H) 0.94 - 1.10 (m, 1 H) 0.72 - 0.93 (m, 2 H).

*Final D-Glu-PSMA-617*

*(3S,10S,14R)-3-[(naphthalen-2-yl)methyl]-1,4,12-trioxo-1-[(1r,4S)-4-({2-[4,7,10-tris(carboxymethyl)-1,4,7,10-tetraazacyclododecan-1-yl]acetamido} methyl)cyclohexyl]-2,5,11,13-tetraazahexadecane-10,14,16-tricarboxylic acid*

Tri-tert-butyl (3S,10S,14R)-3-[(naphthalen-2-yl)methyl]-1,4,12-trioxo-1-[(1r,4S)-4-({2-[4,7,10-tris(2-tert-butoxy-2-oxoethyl)-1,4,7,10-tetraazacyclododecan-1-yl]acetamido}methyl) cyclohexyl]-2,5,11,13-tetraazahexadecane-10,14,16-tricarboxylate (30.0 mg, 21.8 µmol) was solubilized in DCM (280 µL). TFA (840 µL, 11 mmol) was added and the mixture was stirred under argon atmosphere at room temperature overnight. The mixture was evaporated and purified by preparative HPLC (C18, acetonitrile/water with 0.1% formic acid) to give 6.00 mg (95% purity, 25% yield) of the target compound. ^1^H-NMR (400 MHz, DMSO-*d*_6_) δ ppm 11.84 – 12.93 (br s, 4 H) 8.19 – 8.48 (br s, 1 H) 7.98 (br t, *J*=5.45 Hz, 1 H) 7.93 (br d, *J*=8.36 Hz, 1 H) 7.82 – 7.87 (m, 1 H) 7.73 – 7.81 (m, 2 H) 7.68 (s, 1 H) 7.35 – 7.53 (m, 3 H) 6.36 (dd, *J*=15.84, 8.24 Hz, 2 H) 4.53 (td, *J*=9.00, 5.32 Hz, 1 H) 3.97 – 4.17 (m, 3 H) 3.52 – 3.88 (m, 9 H) 2.85 – 3.23 (m, 21 H) 2.15 – 2.28 (m, 2 H) 2.01 – 2.13 (m, 1 H) 1.83 – 1.93 (m, 1 H) 1.54 – 1.76 (m, 5 H) 1.39 – 1.53 (m, 2 H) 1.15 – 1.37 (m, 7 H) 0.97 – 1.13 (m, 1 H) 0.72 – 0.91 (m, 2 H).

### Synthesis of PSMA-617-hydroxyethyl-HOPO

### *Intermediate 1 for PSMA-617-hydroxyethyl-HOPO*

*(3S,10S,14S)-1-{(1r,4S)-4-[({[4-(3-[bis(2-{[3-hydroxy-1-(2-hydroxyethyl)-2-oxo-1,2-dihydropyridine-4-carbonyl]amino}ethyl)amino]-2-{[bis(2-{[3-hydroxy-1-(2-hydroxyethyl)-2-oxo-1,2-dihydropyridine-4-carbonyl]amino}ethyl)amino]methyl} propyl)phenyl]carbamothioyl}amino)methyl]cyclohexyl}-3-[(naphthalen-2-yl)methyl]-1,4,12-trioxo-2,5,11,13-tetraazahexadecane-10,14,16-tricarboxylic acid*

N,N',N'',N'''-({2-[(4-isothiocyanatophenyl)methyl]propane-1,3-diyl}bis[nitrilodi(ethane-2,1-diyl)])tetrakis[3-hydroxy-1-(2-hydroxyethyl)-2-oxo-1,2-dihydropyridine-4-carboxamide] [2] (9.0 mg, 8.0 µmol) and (3S,10S,14S)-1-[(1r,4S)-4-(aminomethyl)cyclohexyl]-3-[(naphthalen-2-yl)methyl]-1,4,12-trioxo-2,5,11,13-tetraazahexadecane-10,14,16-tricarboxylic acid (3.0 mg, 4.6 µmol) were dissolved in 0.1 M carbonate buffer, pH 9.5 (1 mL) and the mixture was heated at 50 °C. The product was purified using preparative HPLC (RP-HPLC using Äkta pure system; column: Phenomenex Luna 5 µm C18(2) 100Å, 250 x 21.2 mm; mobile phase: Water/0.1% TFA; ACN gradient: 20-40% B over 40 min; flow: 10 mL/min; detection: UV 280/335 nm, tR = 26 min) affording 3.0 mg (37% yield) of the target compound. LC-MS (Method 4, gradient: 10-50% B over 3 min): R_t_ = 1.88 min; MS (ESIpos): m/z = 1773.7 [M+H]^+^.

### *Final ^227^Th-PSMA-617-hydroxyethyl-HOPO*

*(3S,10S,14S)-1-{(1r,4S)-4-[({[4-(3-[bis(2-{[3-(hydroxy-kappaO)-1-(2-hydroxyethyl)-2-(oxo-kappaO)-1,2-dihydropyridine-4-carbonyl]amino}ethyl)amino]-2-{[bis(2-{[3-(hydroxy-kappaO)-1-(2-hydroxyethyl)-2-(oxo-kappaO)-1,2-dihydropyridine-4-carbonyl]amino}ethyl)amino]methyl}propyl)phenyl]carbamothioyl}amino)methyl]cyclohexyl}-3-[(naphthalen-2-yl)methyl]-1,4,12-trioxo-2,5,11,13-tetraazahexadecane-10,14,16-tricarboxylato(4-)(^227^Th)thorium*

(3S,10S,14S)-1-{(1r,4S)-4-[({[4-(3-[bis(2-{[3-hydroxy-1-(2-hydroxyethyl)-2-oxo-1,2-dihydropyridine-4-carbonyl]amino}ethyl)amino]-2-{[bis(2-{[3-hydroxy-1-(2-hydroxyethyl)-2-oxo-1,2-dihydropyridine-4-carbonyl]amino}ethyl)amino]methyl} propyl)phenyl]carbamothioyl}amino)methyl]cyclohexyl}-3-[(naphthalen-2-yl)methyl]-1,4,12-trioxo-2,5,11,13-tetraazahexadecane-10,14,16-tricarboxylic acid (6.9 µg) dissolved in 230 µL of 30 mM citrate buffer (pH 5.5) containing 0.5 mg/mL pABA was mixed with thorium-227 in 0.5 M HCl (2 µL) at 0.3 MBq/nmol specific activity and RAC of 3.7 MBq/mL, and the mixture incubated for 60 min. The labelling efficiency was determined to be 97% by iTLC.

**Synthesis of PSMA SMOL-TTCs**

*Reaction sequence:*

**Synthesis of PSMA SMOL conjugate, monomer**

### *Intermediate 1 for PSMA SMOL conjugate, monomer*

*2,2'-{({3-[(2-{[1-(2-{[(6-{[(7S,11S,18S)-7,11-bis(tert-butoxycarbonyl)-2,2-dimethyl-4,9,17-trioxo-19-(quinolin-2-yl)-3-oxa-8,10,16-triazanonadecan-18-yl]carbamoyl}pyridin-3-yl)methyl]amino}-2-oxoethyl)-3-hydroxy-6-methyl-2-oxo-1,2-dihydropyridine-4-carbonyl]amino}ethyl)(2-{[1-(carboxymethyl)-3-hydroxy-6-methyl-2-oxopyridine-4(2H)-carbonyl]amino}ethyl)amino]propyl}azanediyl)bis[(ethane-2,1-diyl)carbamoyl(3-hydroxy-6-methyl-2-oxopyridine-4,1(2H)-diyl)]}diacetic acid*

2,2',2'',2'''-(propane-1,3-diylbis{nitrilobis[(ethane-2,1-diyl)carbamoyl(3-hydroxy-6-methyl-2-oxopyridine-4,1(2H)-diyl)]})tetraacetic acid (3.50 mg, 3.23 µmol) and tri-tert-butyl (3S,10S,14S)-1-[5-(aminomethyl)pyridin-2-yl]-1,4,12-trioxo-3-[(quinolin-2-yl)methyl]-2,5,11,13-tetraazahexadecane-10,14,16-tricarboxylate (2.65 mg, 3.23 µmol) were dissolved in NMP (610 µL). DIPEA (3.8 µL, 22 µmol) was added. PyAOP (2.1 mg, 4.04 µmol) in NMP 170 µL was added. The reaction mix was diluted with 20% ACN/water/0.1% TFA (8 mL) and the products were purified by preparative HPLC (RP-HPLC using Äkta pure system; column: Phenomenex Luna 5 µm C18(2) 100Å, 250 x 21.2 mm; mobile phase: Water/0.1% TFA; ACN gradient: 20-70% B over 40 min; flow: 10 mL/min; detection: UV 280/335 nm, tR product: 28 min) to give 1.6 mg (26% yield) of the target compound. LC-MS (Method 4, gradient: 10-70% B over 3 min): R_t_ = 2.14 min; MS (ESIpos): m/z = 1885.0 [M+H]^+^. ^1^H-NMR (400 MHz, DMSO-*d*_6_) δ ppm 13.00 - 13.44 (m, 2 H), 10.87 - 11.07 (m, 2 H), 9.03 - 9.14 (m, 1 H), 8.49 - 8.64 (m, 4 H), 8.23 - 8.29 (m, 1 H), 8.14 - 8.21 (m, 1 H), 7.78 - 8.01 (m, 5 H), 7.70 - 7.76 (m, 1 H), 7.55 (br t, *J*=7.48 Hz, 1 H), 7.43 (br d, *J*=7.60 Hz, 1 H), 6.37 - 6.44 (m, 2 H), 6.26 (dd, *J*=19.39, 8.49 Hz, 2 H), 4.89 - 5.00 (m, 1 H), 4.65 - 4.83 (m, 5 H), 4.30 - 4.48 (m, 2 H), 3.98 - 4.06 (m, 2 H), 3.87 - 3.94 (m, 3 H), 3.37 - 3.46 (m, 9 H), 2.94 - 3.05 (m, 4 H), 2.52 - 2.53 (m, 8 H), 2.14 - 2.29 (m, 11 H), 1.81 - 1.90 (m, 2 H), 1.62 - 1.73 (m, 2 H), 1.11 - 1.57 (m, 45 H).

*Final PSMA SMOL conjugate, monomer*

*N^6^-{N-[5-({2-[4-({2-[{3-[bis(2-{[1-(carboxymethyl)-3-hydroxy-6-methyl-2-oxo-1,2-dihydropyridine-4-carbonyl]amino}ethyl)amino]propyl}(2-{[1-(carboxymethyl)-3-hydroxy-6-methyl-2-oxo-1,2-dihydropyridine-4-carbonyl]amino}ethyl)amino]ethyl}carbamoyl)-3-hydroxy-6-methyl-2-oxopyridin-1(2H)-yl]acetamido}methyl)pyridine-2-carbonyl]-3-(quinolin-2-yl)-L-alanyl}-N^2^-{[(1S)-1,3-dicarboxypropyl]carbamoyl}-L-lysine*

2,2'-{({3-[(2-{[1-(2-{[(6-{[(7S,11S,18S)-7,11-bis(tert-butoxycarbonyl)-2,2-dimethyl-4,9,17-trioxo-19-(quinolin-2-yl)-3-oxa-8,10,16-triazanonadecan-18-yl]carbamoyl}pyridin-3-yl)methyl]amino}-2-oxoethyl)-3-hydroxy-6-methyl-2-oxo-1,2-dihydropyridine-4-carbonyl]amino}ethyl)(2-{[1-(carboxymethyl)-3-hydroxy-6-methyl-2-oxopyridine-4(2H)-carbonyl]amino}ethyl)amino]propyl}azanediyl)bis[(ethane-2,1-diyl)carbamoyl(3-hydroxy-6-methyl-2-oxopyridine-4,1(2H)-diyl)]}diacetic acid (1.60 mg, 0.849 µmol) was treated with 90% TFA in water (0.5 mL). Water (18 mL) was added and the solution was lyophilised affording 1.40 mg (95% purity, 91% yield) of the target compound. LC-MS (Method 4, gradient: 10-50% B over 3 min): R_t_ = 1.47 min; MS (ESIpos): m/z = 1716.7 [M+H]^+^. ^1^H-NMR (400 MHz, DMSO-*d*_6_) δ ppm 10.85 - 11.24 (br s, 3 H), 9.48 - 9.72 (br s, 1 H), 9.11 (d, *J*=8.11 Hz, 1 H), 8.89 (br d, *J*=4.82 Hz, 1 H), 8.58 (br s, 5 H), 8.29 (br d, *J*=8.11 Hz, 1 H), 8.20 (br t, *J*=5.32 Hz, 1 H), 7.88 - 7.99 (m, 3 H), 7.83 (dd, *J*=8.36, 1.77 Hz, 1 H), 7.71 - 7.78 (m, 1 H), 7.56 (t, *J*=7.48 Hz, 1 H), 7.44 (d, *J*=8.36 Hz, 1 H), 6.36 - 6.42 (m, 4 H), 6.31 (dd, *J*=15.21, 8.36 Hz, 2 H), 4.89 - 4.98 (m, 1 H), 4.75 (s, 8 H), 4.38 - 4.45 (m, 2 H), 4.10 (br d, *J*=5.32 Hz, 1 H), 3.96 - 4.04 (m, 1 H), 3.56 - 3.82 (m, 14 H), 2.94 - 3.10 (m, 5 H), 2.52 - 2.54 (m, 6 H), 2.19 (s, 17 H), 1.86 - 1.97 (m, 1 H), 1.64 - 1.76 (m, 1 H), 1.52 - 1.63 (m, 1 H), 1.38 - 1.50 (m, 1 H), 1.27 - 1.38 (m, 2 H), 1.15 - 1.27 (m, 3 H).

**Synthesis of PSMA SMOL conjugate, dimer**

### *Intermediate 1 for PSMA SMOL conjugate, dimer (potential mixture of 1,1- and 1,2-isomers)*

*2-[4-[2-[2-[[1-[2-[[6-[[(1S)-2-[[(5S)-6-tert-butoxy-5-[[(1S)-4-tert-butoxy-1-tert-butoxycarbonyl-4-oxo-butyl]carbamoylamino]-6-oxo-hexyl]amino]-2-oxo-1-(2-quinolylmethyl)ethyl]carbamoyl]-3-pyridyl]methylamino]-2-oxo-ethyl]-3-hydroxy-6-methyl-2-oxo-pyridine-4-carbonyl]amino]ethyl-[3-[2-[[1-[2-[[6-[[(1S)-2-[[(5S)-6-tert-butoxy-5-[[(1S)-4-tert-butoxy-1-tert-butoxycarbonyl-4-oxo-butyl]carbamoylamino]-6-oxo-hexyl]amino]-2-oxo-1-(2-quinolylmethyl)ethyl]carbamoyl]-3-pyridyl]methylamino]-2-oxo-ethyl]-3-hydroxy-6-methyl-2-oxo-pyridine-4-carbonyl]amino]ethyl-[2-[[1-(carboxymethyl)-3-hydroxy-6-methyl-2-oxo-pyridine-4-carbonyl]amino]ethyl]amino]propyl]amino]ethylcarbamoyl]-3-hydroxy-6-methyl-2-oxo-1-pyridyl]acetic acid*

2,2',2'',2'''-(propane-1,3-diylbis{nitrilobis[(ethane-2,1-diyl)carbamoyl(3-hydroxy-6-methyl-2-oxopyridine-4,1(2H)-diyl)]})tetraacetic acid (3.50 mg, 3.23 µmol) and tri-tert-butyl (3S,10S,14S)-1-[5-(aminomethyl)pyridin-2-yl]-1,4,12-trioxo-3-[(quinolin-2-yl)methyl]-2,5,11,13-tetraazahexadecane-10,14,16-tricarboxylate (2.65 mg, 3.23 µmol) were dissolved in NMP (610 µL). DIPEA (3.8 µL, 22 µmol) was added. PyAOP (2.1 mg, 4.04 µmol) in NMP 170 µL was added. The reaction mix was diluted with 20% can/water/0.1% TFA (8 mL) and the products were purified by preparative HPLC (RP-HPLC using Äkta pure system; column: Phenomenex Luna 5 µm C18(2) 100Å, 250 x 21.2 mm; mobile phase: Water/0.1% TFA; ACN gradient: 20-70% B over 40 min; flow: 10 mL/min; detection: UV 280/335 nm, tR product: 35 min) to give 1.5 mg (17% yield) of the target compound. LC-MS (Method 4, gradient: 10-70% B over 3 min): R_t_ = 2.35 min; MS (ESIpos): m/z = 1344.0 [M+2H]^2+^. ^1^H-NMR (400 MHz, DMSO-*d*_6_) δ ppm 10.85 - 11.17 (m, 3 H), 9.51 - 9.71 (m, 1 H), 9.09 (br d, *J*=8.62 Hz, 2 H), 8.85 - 8.94 (m, 2 H), 8.57 (br d, *J*=1.01 Hz, 6 H), 8.28 (br d, *J*=7.86 Hz, 2 H), 8.14 - 8.22 (m, 2 H), 7.88 - 7.99 (m, 6 H), 7.83 (dd, *J*=7.98, 1.65 Hz, 2 H), 7.74 (br t, *J*=7.60 Hz, 2 H), 7.52 - 7.58 (m, 2 H), 7.43 (br d, *J*=8.62 Hz, 2 H), 6.38 (d, *J*=3.04 Hz, 4 H), 6.27 (dd, *J*=17.11, 8.24 Hz, 4 H), 4.89 - 4.99 (m, 2 H), 4.74 (br s, 7 H), 4.42 (br d, *J*=4.06 Hz, 4 H), 3.99 - 4.07 (m, 4 H), 3.90 (br dd, *J*=7.98, 5.96 Hz, 7 H), 3.38 - 3.44 (m, 8 H), 2.91 - 3.12 (m, 7 H), 2.19 (br d, *J*=2.28 Hz, 18 H), 1.82 - 1.92 (m, 2 H), 1.61 - 1.72 (m, 2 H), 1.48 - 1.58 (m, 3 H), 1.41 - 1.48 (m, 2 H), 1.27 - 1.41 (m, 63 H), 1.14 - 1.25 (m, 7 H).

*Final PSMA SMOL conjugate, dimer (potential mixture of 1,1- and 1,2-isomers)*

*(3S,10S,14S,3'S,10'S,14'S)-1,1'-(propane-1,3-diylbis{[(2-{[1-(carboxymethyl)-3-hydroxy-6-methyl-2-oxo-1,2-dihydropyridine-4-carbonyl]amino}ethyl) azanediyl]ethane-2,1-diylcarbamoyl(3-hydroxy-6-methyl-2-oxopyridine-4,1(2H)-diyl)(1-oxoethane-2,1-diyl)azanediylmethylenepyridine-5,2-diyl})bis{1,4,12-trioxo-3-[(quinolin-2-yl)methyl]-2,5,11,13-tetraazahexadecane-10,14,16-tricarboxylic acid}*

2-[4-[2-[2-[[1-[2-[[6-[[(1S)-2-[[(5S)-6-tert-butoxy-5-[[(1S)-4-tert-butoxy-1-tert-butoxycarbonyl-4-oxo-butyl]carbamoylamino]-6-oxo-hexyl]amino]-2-oxo-1-(2-quinolylmethyl)ethyl]carbamoyl]-3-pyridyl]methylamino]-2-oxo-ethyl]-3-hydroxy-6-methyl-2-oxo-pyridine-4-carbonyl]amino]ethyl-[3-[2-[[1-[2-[[6-[[(1S)-2-[[(5S)-6-tert-butoxy-5-[[(1S)-4-tert-butoxy-1-tert-butoxycarbonyl-4-oxo-butyl]carbamoylamino]-6-oxo-hexyl]amino]-2-oxo-1-(2-quinolylmethyl)ethyl]carbamoyl]-3-pyridyl]methylamino]-2-oxo-ethyl]-3-hydroxy-6-methyl-2-oxo-pyridine-4-carbonyl]amino]ethyl-[2-[[1-(carboxymethyl)-3-hydroxy-6-methyl-2-oxo-pyridine-4-carbonyl]amino]ethyl]amino]propyl]amino]ethylcarbamoyl]-3-hydroxy-6-methyl-2-oxo-1-pyridyl]acetic acid (1.50 mg, 0.558 µmol) was treated with 90% TFA in water (0.5 mL). Water (8 mL) was added and the solution was subjected to preparative HPLC (RP-HPLC using Äkta pure system; column: Phenomenex Luna 5 µm C18(2) 100Å, 250 x 21.2 mm; mobile phase: Water/0.1% TFA; ACN gradient: 10-50% B over 40 min; flow: 10 mL/min; detection: UV 280/335 nm, tR product: 24 min) affording 1.00 mg (95% purity, 72% yield) of the target compound. LC-MS (Method 4, gradient: 10-50% B over 3 min): R_t_ = 1.65 min; MS (ESIpos): m/z = 1175.5 [M+2H]^2+^. ^1^H-NMR (600 MHz, DMSO-*d*_6_) δ ppm 12.18 - 13.40 (br s, 6 H), 10.83 - 11.09 (m, 3 H), 9.53 - 9.85 (br s, 1 H), 9.11 (d, *J*=8.39 Hz, 2 H), 8.91 (br s, 2 H), 8.53 - 8.65 (m, 6 H), 8.34 (br d, *J*=8.01 Hz, 2 H), 8.21 (br t, *J*=5.53 Hz, 2 H), 7.93 - 8.01 (m, 4 H), 7.89 - 7.93 (m, 2 H), 7.83 (dd, *J*=8.20, 1.72 Hz, 2 H), 7.77 (br t, *J*=7.44 Hz, 2 H), 7.59 (t, *J*=7.44 Hz, 2 H), 7.48 (br d, *J*=8.01 Hz, 2 H), 6.39 (d, *J*=5.34 Hz, 4 H), 6.27 - 6.36 (m, 4 H), 4.92 - 5.00 (m, 2 H), 4.75 (br s, 8 H), 4.43 (br d, *J*=4.96 Hz, 4 H), 4.10 (td, *J*=8.30, 5.15 Hz, 4 H), 4.01 (td, *J*=8.01, 5.34 Hz, 5 H), 3.65 - 3.74 (m, 4 H), 3.24 - 3.48 (m, 16 H), 3.06 (dq, *J*=13.21, 6.53 Hz, 3 H), 2.98 (dq, *J*=12.97, 6.61 Hz, 2 H), 2.52 - 2.53 (m, 2 H), 2.17 - 2.30 (m, 16 H), 1.88 - 1.96 (m, 2 H), 1.67 - 1.75 (m, 2 H), 1.56 - 1.64 (m, 2 H), 1.41 - 1.50 (m, 2 H), 1.33 (quin, *J*=7.34 Hz, 4 H), 1.17 - 1.27 (m, 4 H).

### Synthesis of PSMA SMOL conjugate, trimer

### *Intermediate 1 for PSMA SMOL conjugate, trimer*

*2-[4-[2-[3-[bis[2-[[1-[2-[[6-[[(1S)-2-[[(5S)-6-tert-butoxy-5-[[(1S)-4-tert-butoxy-1-tert-butoxycarbonyl-4-oxo-butyl]carbamoylamino]-6-oxo-hexyl]amino]-2-oxo-1-(2-quinolylmethyl)ethyl]carbamoyl]-3-pyridyl]methylamino]-2-oxo-ethyl]-3-hydroxy-6-methyl-2-oxo-pyridine-4-carbonyl]amino]ethyl]amino]propyl-[2-[[1-[2-[[6-[[(1S)-2-[[(5S)-6-tert-butoxy-5-[[(1S)-4-tert-butoxy-1-tert-butoxycarbonyl-4-oxo-butyl]carbamoylamino]-6-oxo-hexyl]amino]-2-oxo-1-(2-quinolylmethyl)ethyl]carbamoyl]-3-pyridyl]methylamino]-2-oxo-ethyl]-3-hydroxy-6-methyl-2-oxo-pyridine-4-carbonyl]amino]ethyl]amino]ethylcarbamoyl]-3-hydroxy-6-methyl-2-oxo-1-pyridyl]acetic acid*

2,2',2'',2'''-(propane-1,3-diylbis{nitrilobis[(ethane-2,1-diyl)carbamoyl(3-hydroxy-6-methyl-2-oxopyridine-4,1(2H)-diyl)]})tetraacetic acid (3.50 mg, 3.23 µmol) and tri-tert-butyl (3S,10S,14S)-1-[5-(aminomethyl)pyridin-2-yl]-1,4,12-trioxo-3-[(quinolin-2-yl)methyl]-2,5,11,13-tetraazahexadecane-10,14,16-tricarboxylate (2.65 mg, 3.23 µmol) were dissolved in NMP (610 µL). DIPEA (3.8 µL, 22 µmol) was added. PyAOP (2.1 mg, 4.04 µmol) in NMP 170 µL was added. The reaction mix was diluted with 20% ACN/water/0.1% TFA (8 mL) and the products were purified by preparative HPLC (RP-HPLC using Äkta pure system; column: Phenomenex Luna 5 µm C18(2) 100Å, 250 x 21.2 mm; mobile phase: Water/0.1% TFA; ACN gradient: 20-70% B over 40 min;flow: 10 mL/min; detection: UV 280/335 nm, tR product: 40 min) to give 1.0 mg (9% yield) of the target compound. LC-MS (Method 4, gradient: 10-70% B over 3 min): R_t_ = 2.90 min; MS (ESIpos): m/z = 1744.4 [M+2H]^2+^. ^1^H-NMR (400 MHz, DMSO-*d*_6_) δ ppm 10.80 - 11.13 (m, 2 H), 9.45 - 9.70 (m, 1 H), 9.03 - 9.19 (m, 2 H), 8.82 - 8.95 (m, 2 H), 8.50 - 8.67 (m, 5 H), 8.08 - 8.35 (m, 4 H), 7.39 - 8.02 (m, 15 H), 6.34 - 6.42 (m, 3 H), 6.26 (br dd, *J*=19.14, 7.98 Hz, 6 H), 4.89 - 5.02 (m, 2 H), 4.69 - 4.79 (m, 5 H), 4.36 - 4.49 (m, 6 H), 3.85 - 4.07 (m, 10 H), 3.22 - 3.48 (m, 15 H), 2.95 - 3.10 (m, 8 H), 2.18 (br s, 18 H), 1.79 - 1.90 (m, 5 H), 1.59 - 1.73 (m, 5 H), 1.47 - 1.56 (m, 5 H), 1.12 - 1.46 (m, 116 H).

*Final PSMA SMOL conjugate, trimer*

*(3S,10S,14S,3'S,10'S,14'S)-1,1'-{({3-[(2-{[1-(2-{[(6-{[(2S)-1-{[(5S)-5-carboxy-5-({[(1S)-1,3-dicarboxypropyl]carbamoyl}amino)pentyl]amino}-1-oxo-3-(quinolin-2-yl)propan-2-yl]carbamoyl}pyridin-3-yl)methyl]amino}-2-oxoethyl)-3-hydroxy-6-methyl-2-oxopyridine-4(2H)-carbonyl]amino}ethyl)(2-{[1-(carboxymethyl)-3-hydroxy-6-methyl-2-oxo-1,2-dihydropyridine-4-carbonyl]amino}ethyl)amino]propyl} azanediyl)bis[(ethane-2,1-diyl)carbamoyl(3-hydroxy-6-methyl-2-oxopyridine-4,1(2H)-diyl)(1-oxoethane-2,1-diyl)azanediylmethylenepyridine-5,2-diyl]}bis{1,4,12-trioxo-3-[(quinolin-2-yl)methyl]-2,5,11,13-tetraazahexadecane-10,14,16-tricarboxylic acid}*

2-[4-[2-[3-[bis[2-[[1-[2-[[6-[[(1S)-2-[[(5S)-6-tert-butoxy-5-[[(1S)-4-tert-butoxy-1-tert-butoxycarbonyl-4-oxo-butyl]carbamoylamino]-6-oxo-hexyl]amino]-2-oxo-1-(2-quinolylmethyl)ethyl]carbamoyl]-3-pyridyl]methylamino]-2-oxo-ethyl]-3-hydroxy-6-methyl-2-oxo-pyridine-4-carbonyl]amino]ethyl]amino]propyl-[2-[[1-[2-[[6-[[(1S)-2-[[(5S)-6-tert-butoxy-5-[[(1S)-4-tert-butoxy-1-tert-butoxycarbonyl-4-oxo-butyl]carbamoylamino]-6-oxo-hexyl]amino]-2-oxo-1-(2-quinolylmethyl)ethyl]carbamoyl]-3-pyridyl]methylamino]-2-oxo-ethyl]-3-hydroxy-6-methyl-2-oxo-pyridine-4-carbonyl]amino]ethyl]amino]ethylcarbamoyl]-3-hydroxy-6-methyl-2-oxo-1-pyridyl]acetic acid (1.00 mg, 0.287 µmol) was treated with 90% TFA in water (0.5 mL). Water (8 mL) was added and the solution was subjected to preparative HPLC (Äkta pure system; column: Phenomenex Luna 5 µm C18(2) 100Å, 250 x 21.2 mm; mobile phase: Water/0.1% TFA; ACN gradient: 10-50% B over 40 min; flow: 10 mL/min; detection: UV 280/335 nm, tR: 27 min) affording 500 µg (95% purity, 56% yield) of the target compound. LC-MS (Method 4, gradient: 10-50% B over 3 min): R_t_ = 1.78 min; MS (ESIpos): m/z = 1492.4 [M+2H]^2+^. ^1^H-NMR (400 MHz, DMSO-*d*_6_) δ ppm 12.15 - 12.90 (br s, 8 H), 10.95 (br s, 3 H), 9.46 - 9.66 (m, 1 H), 9.11 (br d, *J*=7.60 Hz, 3 H), 8.83 - 8.93 (br s, 3 H), 8.57 (s, 7 H), 8.26 (br d, *J*=8.11 Hz, 3 H), 8.15 - 8.22 (m, 3 H), 7.88 - 7.98 (m, 9 H), 7.79 - 7.85 (m, 3 H), 7.73 (br t, *J*=6.97 Hz, 3 H), 7.54 (t, *J*=7.60 Hz, 3 H), 7.42 (d, *J*=8.11 Hz, 3 H), 6.38 (s, 4 H), 6.30 (dd, *J*=15.46, 8.11 Hz, 6 H), 4.89 - 4.98 (m, 3 H), 4.74 (br s, 7 H), 4.37 - 4.46 (m, 6 H), 4.10 (br d, *J*=5.07 Hz, 3 H), 3.95 - 4.05 (m, 4 H), 3.66 - 3.73 (m, 5 H), 3.36 - 3.42 (m, 15 H), 2.92 - 3.10 (m, 10 H), 2.12 - 2.30 (m, 19 H), 1.86 - 1.97 (m, 3 H), 1.66 - 1.76 (m, 3 H), 1.54 - 1.64 (m, 3 H), 1.41 - 1.51 (m, 3 H), 1.23 (br s, 17 H).

###

### Synthesis of PSMA SMOL conjugate, tetramer

### *Intermediate 1 for PSMA SMOL conjugate, tetramer*

*Ditert-butyl (2S)-2-[[(1S)-5-[[(2S)-2-[[5-[[[2-[4-[2-[3-[bis[2-[[1-[2-[[6-[[(1S)-2-[[(5S)-6-tert-butoxy-5-[[(1S)-4-tert-butoxy-1-tert-butoxycarbonyl-4-oxo-butyl]carbamoylamino]-6-oxo-hexyl]amino]-2-oxo-1-(2-quinolylmethyl)ethyl]carbamoyl]-3-pyridyl]methylamino]-2-oxo-ethyl]-3-hydroxy-6-methyl-2-oxo-pyridine-4-carbonyl]amino]ethyl]amino]propyl-[2-[[1-[2-[[6-[[(1S)-2-[[(5S)-6-tert-butoxy-5-[[(1S)-4-tert-butoxy-1-tert-butoxycarbonyl-4-oxo-butyl]carbamoylamino]-6-oxo-hexyl]amino]-2-oxo-1-(2-quinolylmethyl)ethyl]carbamoyl]-3-pyridyl]methylamino]-2-oxo-ethyl]-3-hydroxy-6-methyl-2-oxo-pyridine-4-carbonyl]amino]ethyl]amino] ethylcarbamoyl]-3-hydroxy-6-methyl-2-oxo-1-pyridyl]acetyl]amino]methyl]pyridine-2-carbonyl]amino]-3-(2-quinolyl)propanoyl]amino]-1-tert-butoxycarbonyl-pentyl]carbamoylamino]pentanedioate*

2,2',2'',2'''-(propane-1,3-diylbis{nitrilobis[(ethane-2,1-diyl)carbamoyl(3-hydroxy-6-methyl-2-oxopyridine-4,1(2H)-diyl)]})tetraacetic acid (3.50 mg, 3.23 µmol) and tri-tert-butyl (3S,10S,14S)-1-[5-(aminomethyl)pyridin-2-yl]-1,4,12-trioxo-3-[(quinolin-2-yl)methyl]-2,5,11,13-tetraazahexadecane-10,14,16-tricarboxylate (2.65 mg, 3.23 µmol) were dissolved in NMP (610 µL). DIPEA (3.8 µL, 22 µmol) was added. PyAOP (2.1 mg, 4.04 µmol) in NMP 170 µL was added. The reaction mix was diluted with 20% ACN/water/0.1% TFA (8 mL) and the products were purified by preparative HPLC (RP-HPLC using Äkta pure system; column: Phenomenex Luna 5 µm C18(2) 100Å, 250 x 21.2 mm; mobile phase: Water/0.1% TFA; ACN gradient: 20-70% B over 40 min; flow: 10 mL/min; detection: UV 280/335 nm, tR product: 43 min) to give 1.1 mg (8% yield) of the target compound.

LC-MS (Method 4, gradient: 10-70% B over 3 min): R_t_ = 3.15 min; MS (ESIpos): m/z = 1430.4 [M+3H]^3+^. ^1^H-NMR (400 MHz, DMSO-*d*_6_) δ ppm 10.96 (br d, *J*=0.76 Hz, 3 H), 9.52 - 9.69 (m, 1 H), 9.08 (d, *J*=8.36 Hz, 4 H), 8.84 - 8.93 (m, 3 H), 8.53 - 8.64 (m, 7 H), 8.31 (br d, *J*=8.36 Hz, 4 H), 8.19 (br t, *J*=5.58 Hz, 4 H), 7.87 - 7.98 (m, 12 H), 7.82 (dd, *J*=8.36, 1.77 Hz, 4 H), 7.75 (br t, *J*=8.11 Hz, 4 H), 7.56 (t, *J*=7.73 Hz, 4 H), 7.46 (br d, *J*=8.62 Hz, 4 H), 6.38 (s, 4 H), 6.27 (dd, *J*=18.76, 8.11 Hz, 8 H), 4.90 - 4.98 (m, 5 H), 4.71 - 4.77 (m, 7 H), 4.39 - 4.45 (m, 10 H), 3.98 - 4.07 (m, 6 H), 3.88 - 3.96 (m, 5 H), 3.60 - 3.78 (m, 8 H), 3.39 - 3.46 (m, 9 H), 2.95 - 3.09 (m, 9 H), 2.11 - 2.29 (m, 20 H), 1.80 - 1.92 (m, 4 H), 1.60 - 1.73 (m, 4 H), 1.49 - 1.59 (m, 5 H), 1.11 - 1.47 (m, 136 H).

*Final PSMA SMOL conjugate, tetramer*

*(2S)-2-[[(1S)-5-[[(2S)-2-[[5-[[[2-[4-[2-[3-[bis[2-[[1-[2-[[6-[[(1S)-2-[[(5S)-5-carboxy-5-[[(1S)-1,3-dicarboxypropyl]carbamoylamino]pentyl]amino]-2-oxo-1-(2-quinolylmethyl)ethyl]carbamoyl]-3-pyridyl]methylamino]-2-oxo-ethyl]-3-hydroxy-6-methyl-2-oxo-pyridine-4-carbonyl]amino]ethyl]amino]propyl-[2-[[1-[2-[[6-[[(1S)-2-[[(5S)-5-carboxy-5-[[(1S)-1,3-dicarboxypropyl]carbamoylamino]pentyl]amino]-2-oxo-1-(2-quinolylmethyl)ethyl]carbamoyl]-3-pyridyl]methylamino]-2-oxo-ethyl]-3-hydroxy-6-methyl-2-oxo-pyridine-4-carbonyl]amino]ethyl]amino]ethylcarbamoyl]-3-hydroxy-6-methyl-2-oxo-1-pyridyl]acetyl]amino]methyl]pyridine-2-carbonyl]amino]-3-(2-quinolyl)propanoyl]amino]-1-carboxy-pentyl]carbamoylamino]pentanedioic acid*

Ditert-butyl (2S)-2-[[(1S)-5-[[(2S)-2-[[5-[[[2-[4-[2-[3-[bis[2-[[1-[2-[[6-[[(1S)-2-[[(5S)-6-tert-butoxy-5-[[(1S)-4-tert-butoxy-1-tert-butoxycarbonyl-4-oxo-butyl]carbamoylamino]-6-oxo-hexyl]amino]-2-oxo-1-(2-quinolylmethyl)ethyl]carbamoyl]-3-pyridyl]methylamino]-2-oxo-ethyl]-3-hydroxy-6-methyl-2-oxo-pyridine-4-carbonyl]amino]ethyl]amino]propyl-[2-[[1-[2-[[6-[[(1S)-2-[[(5S)-6-tert-butoxy-5-[[(1S)-4-tert-butoxy-1-tert-butoxycarbonyl-4-oxo-butyl]carbamoylamino]-6-oxo-hexyl]amino]-2-oxo-1-(2-quinolylmethyl)ethyl]carbamoyl]-3-pyridyl]methylamino]-2-oxo-ethyl]-3-hydroxy-6-methyl-2-oxo-pyridine-4-carbonyl]amino]ethyl]amino]ethylcarbamoyl]-3-hydroxy-6-methyl-2-oxo-1-pyridyl]acetyl]amino]methyl]pyridine-2-carbonyl]amino]-3-(2-quinolyl)propanoyl]amino]-1-tert-butoxycarbonyl-pentyl]carbamoylamino]pentanedioate (1.10 mg, 0.256 µmol) was treated with 90% TFA in water (0.5 mL). Water (18 mL) was added and solution affording 900 µg (95% purity, 92% yield) of the target compound. LC-MS (Method 4, gradient: 10-50% B over 3 min): R_t_ = 1.83 min; MS (ESIpos): m/z = 1206.2 [M+3H]^3+^.

**REFERENCES FOR THE SUPPLEMENTARY INFORMATION**

1. Pailloux SL, Nguyen S, Zhou S, Hom ME, Keyser MN, Smiles D, et al. Synthesis and chemical reactivity of a 6-Me-3,2-hydroxypyridinone dithiazolide with primary amines: a route to new hexadentate chelators for hard metal(III) ions. J Heterocycl Chem. 2016;53:1065-73. doi:10.1002/jhet.2372.

2. Bonge-Hansen HT, Ryan OB. Radio-pharmaceutical complexes. 2013;Patent WO2013167756A1:p. 43.
