# Supplementary material for: Mono- and multimeric PSMA-targeting small molecule-thorium-227 conjugates for optimized efficacy and biodistribution in preclinical models
Source: Eur J Nucl Med Mol Imaging. 2023 Oct 26;51(3):669–80. doi: 10.1007/s00259-023-06474-z (PMC10796422; doi:10.1007/s00259-023-06474-z)
Supplement: Supplementary file 2 — Supplementary file2 (DOCX 9890 KB) [file 259_2023_6474_MOESM2_ESM.docx]

**Supplementary results for:**

**Mono- and multimeric PSMA-targeting small molecule-thorium-227 conjugates for optimized efficacy and biodistribution in preclinical models**

***European Journal of Nuclear Medicine and Molecular Imaging***

Niels Böhnke^1^, Bård Indrevoll^2^, Stefanie Hammer^1^, Alex Papple^2^, Alexander Kristian^2^, Hans Briem^1^, Arif Celik^1^, Dominik Mumberg^1,3^, Alan Cuthbertson^2^, Sabine Zitzmann-Kolbe^1^

^1^Bayer AG, Pharmaceuticals, Berlin, Germany;

^2^Bayer AS, Oslo, Norway

^3^Current address: Adcendo ApS, Copenhagen, Denmark

**Corresponding author:** Sabine Zitzmann-Kolbe, Bayer AG, Research & Early Development, Pharmaceuticals, 13342 Berlin, Germany. E-mail: sabine.zitzmann-kolbe@bayer.com

**SUPPLEMENTAL FIGURES**

**Supplemental Figure 1**

**Supplemental Fig. 1**

**Decay cascade of thorium-227 purified from an actinium-227 source**

d, days; m, minutes; ms, milliseconds; s, seconds; y, years.

**Supplemental Figure 2**

**Supplemental Fig. 2**

**Chemical structures of PSMA-617, the chelators, and the interim compound PSMA-617-hydroxyethyl-HOPO**

**Supplemental Figure 3**

**Supplemental Fig. 3**

**Chemical structures of the (a) dimeric, (b) trimeric, and (c) tetrameric variants of the PSMA SMOL conjugates coupled with carboxy-HOPO chelates via optimized linker motifs**

^a^The dimer should be considered a potential mixture of the 1,1- and 1,2-isomers (please see **Online Resource**, **Suppl. Fig. 5**).

**Supplemental Figure 4**

**
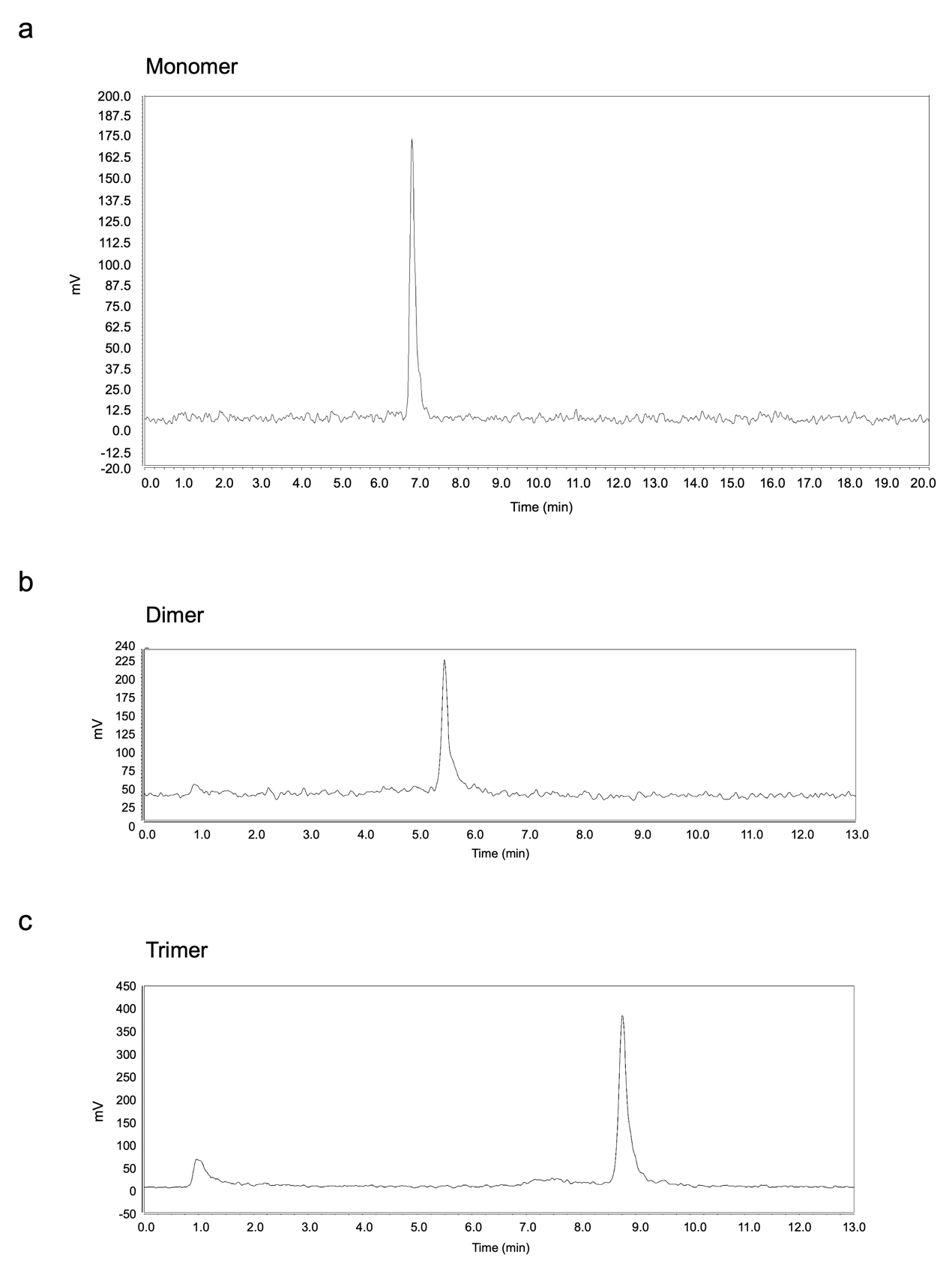
**

**Supplemental Fig. 4**

**The radiochemical purity of the (a) monomeric, (b) dimeric, and (c) trimeric PSMA SMOL-TTCs was determined using radio-HPLC**

mV, millivolt.

**Supplemental Figure 5**

**Supplemental Fig. 5**

**Structures of the two possible constitutional dimer isomers**

**Supplemental Figure 6**

**Supplemental Fig. 6**

**Binding and internalization of PSMA SMOL conjugates on LNCaP PrCa cells after a (a) 30-min and (b) 2-h incubation.** The conjugates were used at 10 nM at a total radioactivity of 375 kBq/nmol.

**Supplemental Figure 7**

**Supplemental Fig. 7**

**Stability of PSMA SMOL-TTC (monomer) in human and mouse serum**

RCP, radiochemical purity

**Supplemental Figure 8**

**Supplemental Fig. 8**

**Biodistribution of PSMA SMOL-TTCs in ST1273 tumor-bearing mice**

Thorium-227 activity was measured in (a) salivary glands, (b) femur, (c) spleen, (d) blood, and (e) heart at 5 min, 2 h, 24 h, 72 h, and 168 h after PSMA SMOL-TTC injection (1.5 MBq/kg, i.v.).

**Supplemental Figure 9**

**Supplemental Fig. 9**

**Biodistribution of ^89^Zr-labeled PSMA SMOL conjugates *in vivo* in LNCaP tumor-bearing mice as determined by PET imaging**

**a.** Representative images of the biodistribution of monomer, dimer, and trimer variants of ^89^Zr-labeled PSMA SMOL conjugates (3 MBq/mouse, i.v.) in the organs of mice 2 h after injection (n=2/group). %ID/g, injected dose per gram.

**b.-c.** Biodistribution of monomer, dimer, and trimer variants of ^89^Zr-labeled PSMA SMOL conjugates (3 MBq/mouse, i.v.) in (B) tumors and (C) kidneys of mice 2 h, 24 h, 72 h, and 144 (monomer and dimer) or 168 h (trimer) after injection (n=2/group). %ID/g, injected dose per gram.

**Supplemental Figure 10**

**Supplemental Fig. 10**

**Efficacy of PSMA SMOL-TTCs in PrCa PDX models shown as tumor growth curves of individual mice**

**a.-e.** Growth curves of ST1273 PDX tumors in mice treated with vehicle (SD, i.v.) or PSMA SMOL-TTC variants (1.5 MBq/kg, SD, i.v.).

**f.-j.** Growth curves of KUCaP-1 PDX tumors in mice treated with vehicle (QW2, i.v.) or PSMA SMOL-TTC monomer or dimer (1 or 2 MBg/kg, Q4Wx2, i.v.).

**Supplemental Figure 11**

**Supplemental Fig. 11**

**Biodistribution of ^227^Th-PSMA-617 (200 kBq/kg, i.v.) in minipigs at 1 h, 24 h, and 72 h post-injection (n=2/time point)**

**SUPPLEMENTARY TABLES**

**Supplementary Table 1. Stability of PSMA SMOL-TTCs in citrate buffer or PBS.**

|  | **RCP (%)** | | | | |
| --- | --- | --- | --- | --- | --- |
|  | **Undiluted** | **Citrate buffer (pH 4.5)** | | **PBS** | |
|  | **0 h** | **6 h** | **48 h** | **6 h** | **48 h** |
| PSMA SMOL-TTC, monomer | 84.1 | 29.3 | 45.9 | 80.9 | 72.4 |
| PSMA SMOL-TTC, dimer | 98.6 | 32.7 | 35.1 | 98.5 | 98.1 |
| PSMA SMOL-TTC, trimer | 100.0 | 68.9 | 71.8 | 100.0 | 100.0 |
| PSMA SMOL-TTC, tetramer | 99.0 | 49.3 | 45.2 | 98.9 | 100.0 |
| RCP, radiochemical purity determined by instant thin-layer chromatography (iTLC) | | | | | |

**Supplementary Table 2. Radiochemical stability of [^89^Zr]PSMA SMOL (monomer).**

| Sample type | **Time**  **point** | **Sample**  **pH** | **Total activity^a^**  **in sample**  (μCi) | **% of total activity^a^** | | **RCP^b^**  **in supernatant**  (%) |
| --- | --- | --- | --- | --- | --- | --- |
|  |  |  |  | Supernatant (%) | Pellet (%) |  |
| Formulation (PBS/HEPES) | 0 |  |  |  |  | 99.1 |
|  | 96 |  |  |  |  | 68.2 |
| Monkey serum | 0 | 7.5 | 94 | 94.7 (89.1) | 10.9 | 80.3 |
|  | 0.5 | 8.5 | 87 | 96.6 (80.4) | 19.6 | 71.2 |
|  | 4 | 8.5 | 90 | 91.1 (90.6) | 9.4 | 58.8 |
|  | 7 | 8.5 | 87 | 97.7 (93.1) | 6.9 | 59.6 |
|  | 24 | 8.5 | 74 | 97.3 (93.2) | 6.8 | 47.0 |
|  | 96 | 8.5 | 40 | 97.5 (90.0) | 10.0 | 33.1 |
|  | 168 | 8.5 | 23 | 95.7 (95.4) | 4.6 | 30.6 |
| ^a^Total activity in the aliquots at each time point was measured using a dose calibrator. After centrifugation, activity was measured in the supernatants and pellets using a dose calibrator and a gamma counter, respectively.  ^b^RCP, radiochemical purity in the supernatant was measured by HPLC. | | | | | | |
